# Evaluation of vector susceptibility in *Aedes aegypti* and *Culex pipiens pallens* to Tibet orbivirus

**DOI:** 10.1128/msphere.00062-24

**Published:** 2024-03-26

**Authors:** Nanjie Ren, Qianqian Jin, Fei Wang, Doudou Huang, Cihan Yang, Wahid Zaman, Ferdinand Villanueva Salazar, Qiyong Liu, Zhiming Yuan, Han Xia

**Affiliations:** 1 Key Laboratory of Virology and Biosafety, Wuhan Institute of Virology, Chinese Academy of Sciences, Wuhan, Hubei, China; 2 University of Chinese Academy of Sciences, Beijing, China; 3 Department of Medical Entomology, Research Institute for Tropical Medicine, Muntinlupa, Philippines; 4 National Key Laboratory of Intelligent Tracking and Forecasting for Infectious Diseases, National Institute for Communicable Disease Control and Prevention, Chinese Center for Disease Control and Prevention, Beijing, China; 5 Hubei Jiangxia Laboratory, Wuhan, China; University of Michigan, Ann Arbor, Michigan, USA

**Keywords:** Tibet orbivirus, mosquito, *Ae. aegypti*, *Cx. p. pallens*, vector susceptibility, immune response

## Abstract

Mosquito-borne viruses cause various infectious diseases in humans and animals. Tibet orbivirus (TIBOV), a newly identified arbovirus, efficiently replicates in different types of vertebrate and mosquito cells, with its neutralizing antibodies detected in cattle and goats. However, despite being isolated from *Culicoides* midges*, Anopheles,* and *Culex* mosquitoes, there has been a notable absence of systematic studies on its vector competence. Thus, in this study, *Aedes aegypti* and *Culex pipiens pallens* were reared in the laboratory to measure vector susceptibility through blood-feeding infection. Furthermore, RNA sequencing was used to examine the overall alterations in the *Ae. aegypti* transcriptome following TIBOV infection. The results revealed that *Ae. aegypti* exhibited a high susceptibility to TIBOV compared to *Cx. p. pallens*. Effective replication of the virus in *Ae. aegypti* midguts occurred when the blood-feeding titer of TIBOV exceeded 10^5^ plaque-forming units mL^−1^. Nevertheless, only a few TIBOV RNA-positive samples were detected in the saliva of *Ae. aegypti* and *Cx. p. pallens*, suggesting that these mosquito species may not be the primary vectors for TIBOV. Moreover, at 2 dpi of TIBOV, numerous antimicrobial peptides downstream of the Toll and Imd signaling pathways were significantly downregulated in *Ae. aegypti*, indicating that TIBOV suppressed mosquitos’ defense to survive in the vector at an early stage. Subsequently, the stress-activated protein kinase JNK, a crucial component of the MAPK signaling pathway, exhibited significant upregulation. Certain genes were also enriched in the MAPK signaling pathway in TIBOV-infected *Ae. aegypti* at 7 dpi.

**IMPORTANCE:**

Tibet orbivirus (TIBOV) is an understudied arbovirus of the genus *Orbivirus*. Our study is the first-ever attempt to assess the vector susceptibility of this virus in two important mosquito vectors, *Aedes aegypti* and *Culex pipiens pallens*. Additionally, we present transcriptome data detailing the interaction between TIBOV and the immune system of *Ae. aegypti*, which expands the knowledge about orbivirus infection and its interaction with mosquitoes.

## INTRODUCTION

The genus *Orbivirus* belongs to the family *Sedoreoviridae,* order *Reovirales* having a genome of 10 linear segments of dsRNA packaged within a triple-layered icosahedral protein capsid (1). Among the genus *Orbivirus*, several viruses have been associated with human diseases, such as tick-associated Kemerovo virus (2) and sand fly-associated Changuinola virus (3). In addition, a considerable number of viruses in this genus are important animal pathogens; for instance, midge-associated bluetongue virus (BTV), African horse sickness virus, and epizootic hemorrhagic disease virus (EHDV) cause acute disease with high mortality in domestic animals, leading to huge economic losses in the livestock industry (4, 5). The emergence of orbiviruses depends on the distribution, activity, and seasonal abundance of competent vectors, such as the adults of certain midge (*Culicoides*) species (6). In addition, orbiviruses have been found in a wide host range including ticks, mosquitoes, midges, ruminants, birds, and humans (7–11).

The Tibet orbivirus (TIBOV) was first isolated from *Anopheles maculatus* mosquitoes collected in 2009 in Motuo county, Tibet, China, by inoculating the mosquito homogenates into cell lines (12). Subsequently, different TIBOV strains were isolated from *Culex quinquefasciatus* (13), *Culex tritaeniorhynchus* (14), *Culicoides* spp. (15–19), and from sentinel cattle (20) in China and Japan (21). So far, several studies have shown that TIBOV can infect various cell lines from humans, animals, and mosquitoes (13, 22) and that it is highly lethal to suckling mice upon intracerebral inoculation (17). Moreover, neutralizing antibodies against TIBOV have been detected in livestock such as cattle and goats (14, 17, 20). These findings indicate that TIBOV could be a potential pathogen for animals.

*Aedes aegypti* and *Culex pipiens pallens* are two important mosquito vectors for arbovirus. In China, *Ae. aegypti* has been observed in limited regions in Yunnan, Hainan, and Guangdong (23), whereas *Cx. p. pallens* is widely distributed in the central, eastern, and northern parts of the country (24). Currently, except for the two Italian mosquito populations of *Culex pipiens* and *Aedes albopictus* showing resistance to BTV through blood-feeding infection (25), most vector competence studies for orbiviruses were conducted on *Culicoides* spp. (26, 27). These therefore highlight the need for more research on the mosquito vector competence for orbiviruses.

In our previous study, TIBOV (YN15-283-01) was isolated from *Culicoides* spp., and its infection characteristics were studied *in vitro* (22). In this study, we established an experimental infection model using *Ae. aegypti* and *Cx. p. pallens* mosquitoes to investigate and compare their susceptibility to TIBOV and further analyze the immune response of *Ae. aegypti* after infection.

## RESULTS

### The susceptibility of two mosquito species to TIBOV

**Fig 1:**
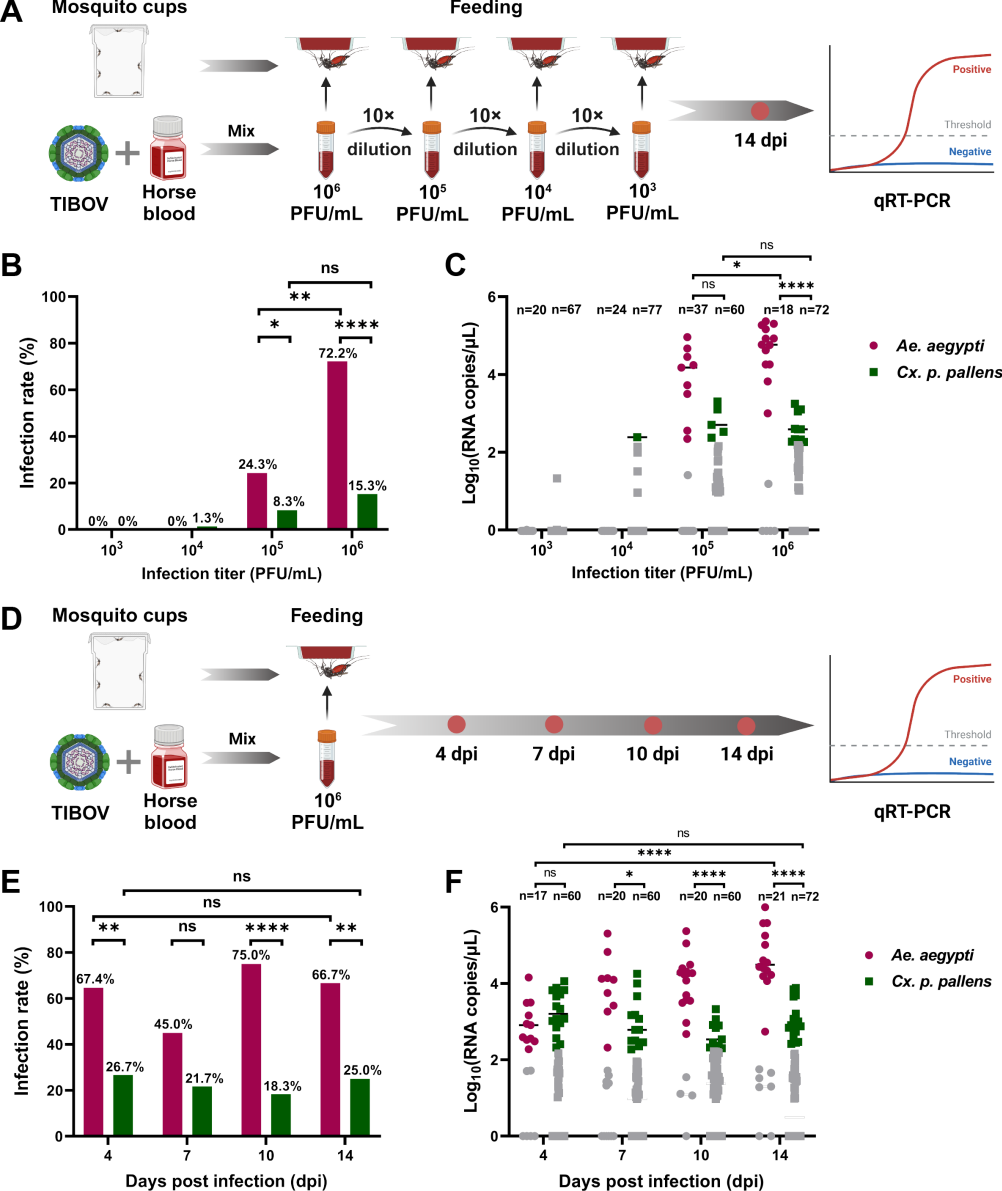
TIBOV infection rates of adult *Ae. aegypti* and *Cx. p. pallens* through blood feeding. (**A**) A continuous dilution of TIBOV from 10^6^ to 10^3^ PFU/mL in blood meal was fed to *Ae. aegypti* and *Cx. p. pallens*, respectively, and then every single mosquito was harvested at 14 dpi for detection. (**B**) Infection rate in *Ae. aegypti* and *Cx. p. pallens* infected with different titers of TIBOV. (**C**) Viral RNA copies/μL in each single mosquito fed with different titers of TIBOV. (**D**) *Ae. aegypti* and *Cx. p. pallens* were fed by blood meal with 10^6^ PFU/mL TIBOV and then mosquitoes were collected and tested at 4, 7, 10, and 14 dpi. (**E**) and (**F**) Infection rate and viral RNA copies/μL of *Ae. aegypti* and *Cx. p. pallens* on different days. Each dot represents an individual mosquito, and the gray dot stands for the negative sample with Ct value > 35. The infection rates were analyzed with Fisher’s exact test, and viral RNA copies/μL were analyzed with non-parametric Mann-Whitney test (non-multiple comparisons) (*P ≤ 0.05, **P ≤ 0.01, ***P ≤ 0.005, and ns: no significant difference).

Adult female *Ae. aegypti* and *Cx. p. pallens* were infected with TIBOV through blood feeding with a viral titer ranging from 10^6^ to 10^3^ plaque-forming units mL^−1^ (PFU/mL) (Fig. 1A). As shown in Fig. 1B, the infection rate of TIBOV at 14 days post-infection (dpi) significantly increased (*P* = 0.0011) from 24.3% to 72.2% when TIBOV titer in the blood meal was changed from 10^5^ to 10^6^ PFU/mL. However, there was no significant difference in the infection rates between infection titers of 10^5^ and 10^6^ PFU/mL as observed in *Cx. p. pallens*. For the blood meal with TIBOV titers of 10^4^ and 10^3^ PFU/mL, both *Cx. p. pallens* and *Ae. aegypti* were nearly free of infection. In contrast, when fed with a blood meal containing 10^6^ and 10^5^ PFU/mL of TIBOV, the infection rates in *Ae. aegypti* were significantly higher than those in *Cx. p. pallens* (10^6^ PFU/mL: *P* < 0.0001; 10^5^ PFU/mL: *P* = 0.039). Moreover, in Fig. 1C, the viral RNA copies in infected *Ae. aegypti* in the group of 10^6^ PFU/mL infection titer were notably higher than those in *Cx. p. pallens* fed with the same titer (Mann-Whitney *U* = 3, *P* = 0.000006) and also higher than the viral copies in *Ae. aegypti* with 10^5^ PFU/mL infection titer (Mann-Whitney *U* = 24, *P* = 0.0208).

Based on these results, the blood meal with 10^6^ PFU/mL TIBOV was selected to examine the viral dynamics *in vivo*. Both *Ae. aegypti* and *Cx. p. pallens* were harvested and tested at 4, 7, 10, and 14 days after infection (dpi) (as shown in Fig. 1D). The infection rates of the two mosquito species remained stable and did not change significantly over time. Notably, the infection rates in *Ae. aegypti* were consistently higher than those in *Cx. p. pallens*, with the exception of 7 dpi (D4: *P* = 0.008; D10: *P* < 0.0001; and D14: *P* = 0.0012) (Fig. 1E). TIBOV RNA copies in *Ae. aegypti* increased over time (Mann-Whitney *U* = 7, *P* < 0.0001), but there was no significant increase observed in RNA copies from *Cx. p. pallens* (Fig. 1F). Additionally, the viral RNA copies in *Ae. aegypti* at 7, 10, and 14 dpi were all higher than those in *Cx. p. pallens* (D7: Mann-Whitney *U* = 23, *P* = 0.0162; D10: Mann-Whitney *U* = 7, *P* = 0.000007; and D14: Mann-Whitney *U* = 12, *P* = 0.000001).

These results indicate that only high titers (≥10^5^ PFU/mL) of TIBOV could establish an effective infection in both *Ae. aegypti* and *Cx. p. pallens*, with *Ae. aegypti* showing greater susceptibility to TIBOV.

### Dissemination and transmission of TIBOV in infected mosquitoes

**Fig 2:**
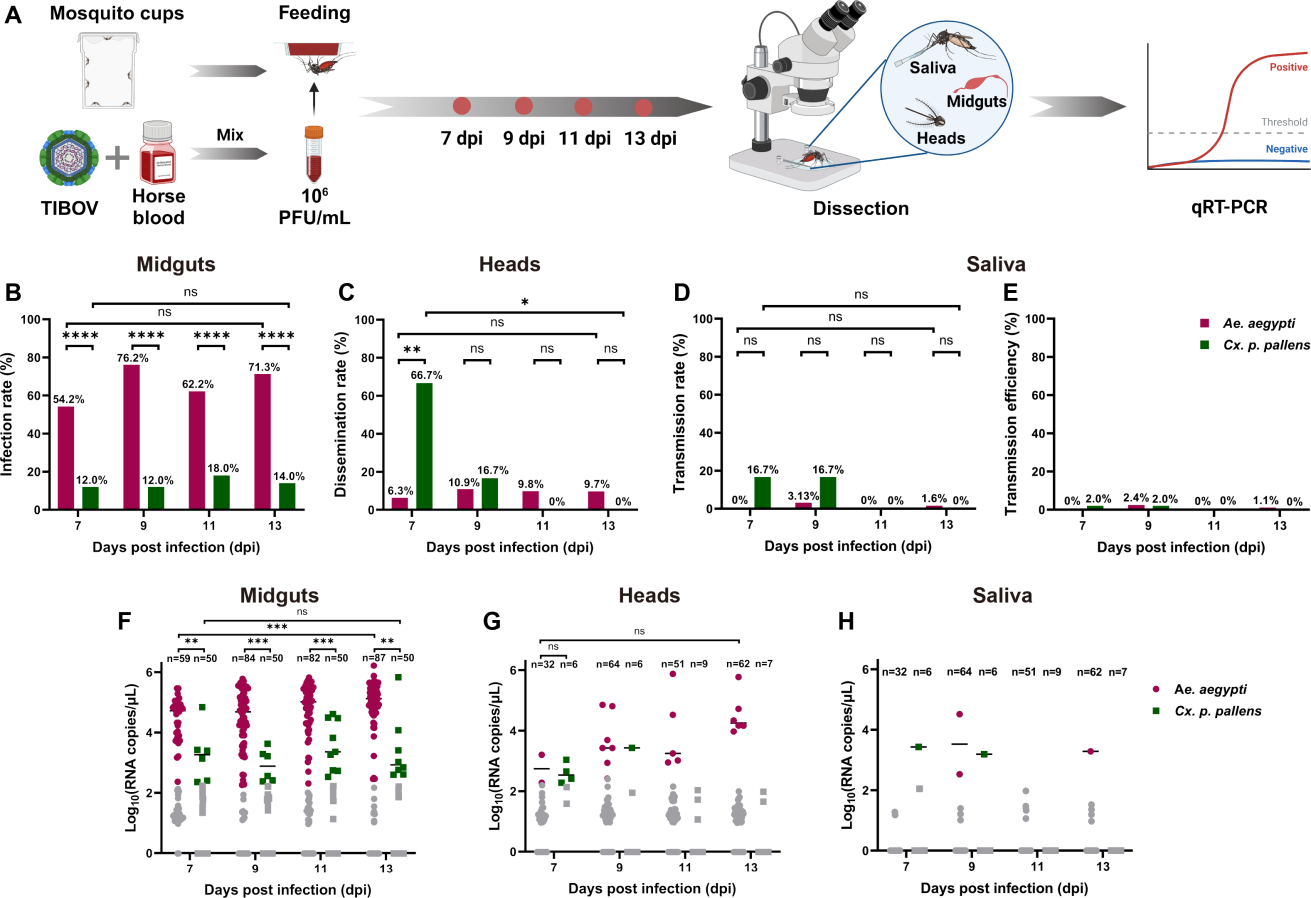
TIBOV dissemination and transmission in *Ae*. *aegypti* and *Cx. p. pallens.* (**A**) Mosquitoes fed with blood meal containing 10^6^ PFU/mL TIBOV were collected at 7, 9, 11, and 13 dpi for dissection and viral RNA detection. (**B**) and (**F**) Infection rates and viral RNA copies in mosquito midguts. (**C**) and (**G**) Dissemination rates and viral RNA copies in mosquito heads. (**D**), (**E**), and (**H**) Transmission rates, transmission efficiency, and viral RNA copies in mosquito saliva samples. Each dot represents an individual mosquito, and the gray dot stands for the negative sample with Ct value > 35. Infection rates were analyzed with Fisher’s exact test, and viral RNA copies were analyzed with non-parametric Mann-Whitney test (non-multiple comparisons, *P ≤ 0.05, **P ≤ 0.01, ***P ≤ 0.005, and ns: no significant difference).

To determine the dissemination and transmission of TIBOV in *Ae. aegypti* and *Cx. p. pallens*, midguts (for infection rate), heads (for dissemination rate), and saliva (for transmission rate and transmission efficiency) were harvested and detected at different time points (Fig. 2A). As shown in Fig. 2B and F, all infection rates and viral RNA copies of *Ae. aegypti* were significantly higher than that of *Cx. p. pallens* at 7 dpi (*P* < 0.0001; Mann-Whitney *U* = 29, *P* = 0.005247), 9 dpi (*P* < 0.0001; Mann-Whitney *U* = 36, *P* = 0.000292), 11 dpi (*P* < 0.0001; Mann-Whitney *U* = 68, *P* = 0.00039), and 13 dpi (*P* < 0.0001; Mann-Whitney *U* = 84, *P* = 0.0015). Similar to the result in “The susceptibility of two mosquito species to TIBOV” section, the infection rates of *Ae. aegypti* and *Cx. p. pallens* did not increase over time (Fig. 2B), while the number of TIBOV RNA copies of *Ae. aegypti* midguts increased significantly (Mann-Whitney *U* = 519, *P* = 0.0001) (Fig. 2F). For dissemination rates, there was no difference between *Ae. aegypti* and *Cx. p. pallens* except on 7 dpi (Fig. 2C), and the number of viral copies did not increase significantly (Fig. 2G). Because few viral-positive midguts and saliva samples were detected in *Cx. p. pallens* (Fig. 2D and H), transmission efficiency assessment was added to show the potential transmission for the population. As shown in Fig. 2E, only 0%–2.4% transmission efficiency was observed in both *Ae. aegypti* and *Cx. p. pallens*.

**Fig 3:**
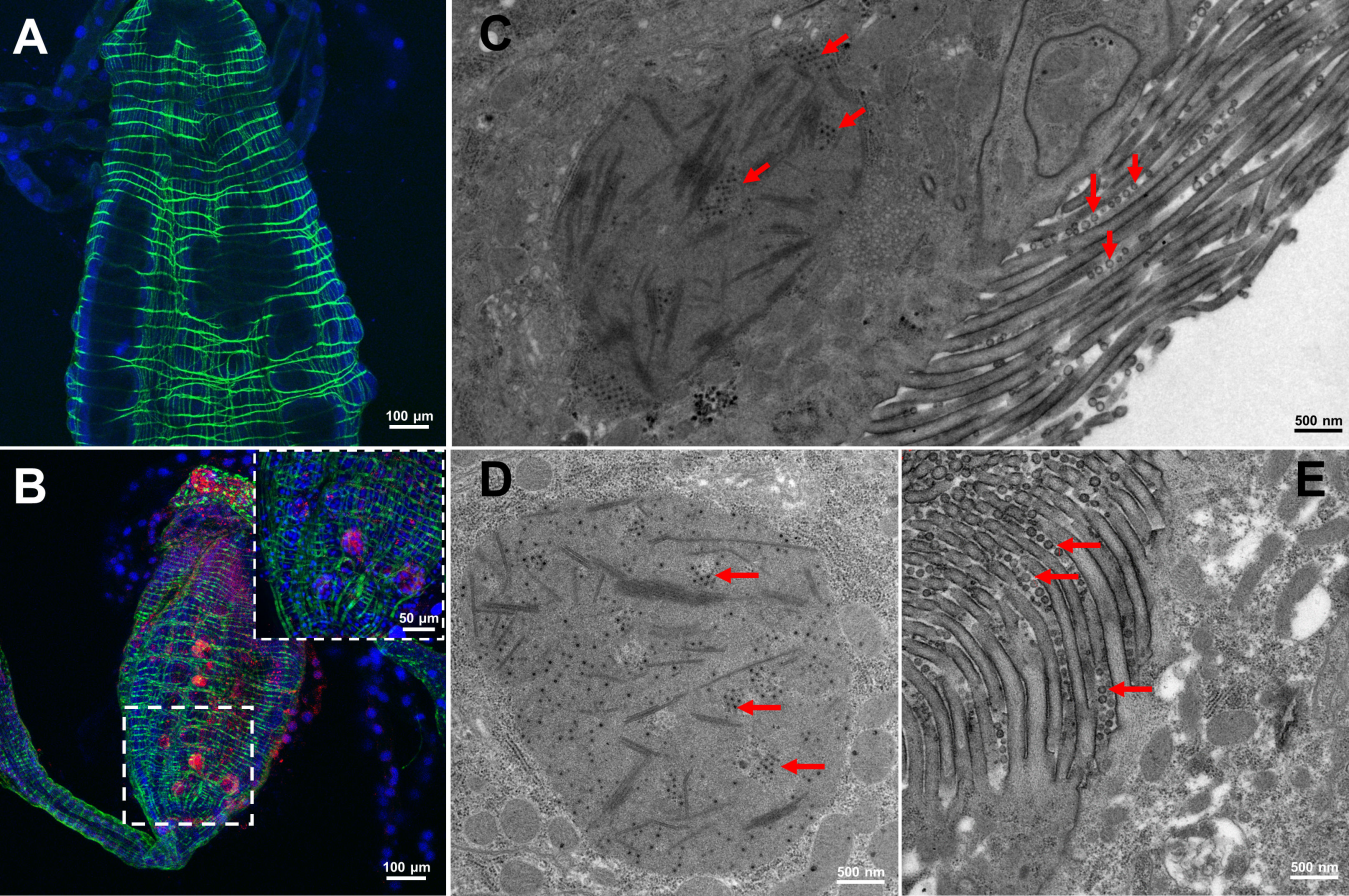
Immunofluorescence visualization and electron micrographs of TIBOV antigen and particles in *Ae. aegypti* midguts. (**A**) and (**B**) Immunolocalization of TIBOV in the midguts of mosquitoes feeding on blood meal without or with TIBOV at 14 dpi. Phalloidin was used to stain F-actin filaments (green). DAPI was used to label the cell’s nucleus (blue). A goat anti-rabbit IgG tagged with a red-fluorescent secondary antibody and a rabbit anti-TIBOV polyclonal antibody were used to identify TIBOV virion clusters. (**C–E**) Viral particles observed in the midguts of mosquitoes feeding on TIBOV blood meal at 14 dpi, as indicated by red arrows on electron micrographs.

As *Ae. aegypti* midguts were more susceptible to TIBOV infection than *Cx. p. pallens*, immunofluorescence assay was performed on *Ae. aegypti* only. In the mosquitoes at 14 dpi, TIBOV were accumulated around the cytoskeleton in midgut endothelial cells of mosquitoes following feeding on a TIBOV blood meal (Fig. 3A and B; Fig. S4). From the ultrasections of the midguts of the viral-infected mosquitoes, granular virus-like particles (VLPs) (20–30 nm in diameter) and long tubular structures were stacked in intracellular vesicles in cells, while spherical shaped VLPs (70–90 nm in diameter) were obvious in the intestinal villus (Fig. 3C through E).

These results indicated that TIBOV could replicate or remain in the midguts of mosquitoes but rarely spread into the salivary glands as detected from saliva samples.

### Several immune-related genes were differentially expressed in TIBOV-infected *Ae. aegypti*

**Fig 4:**
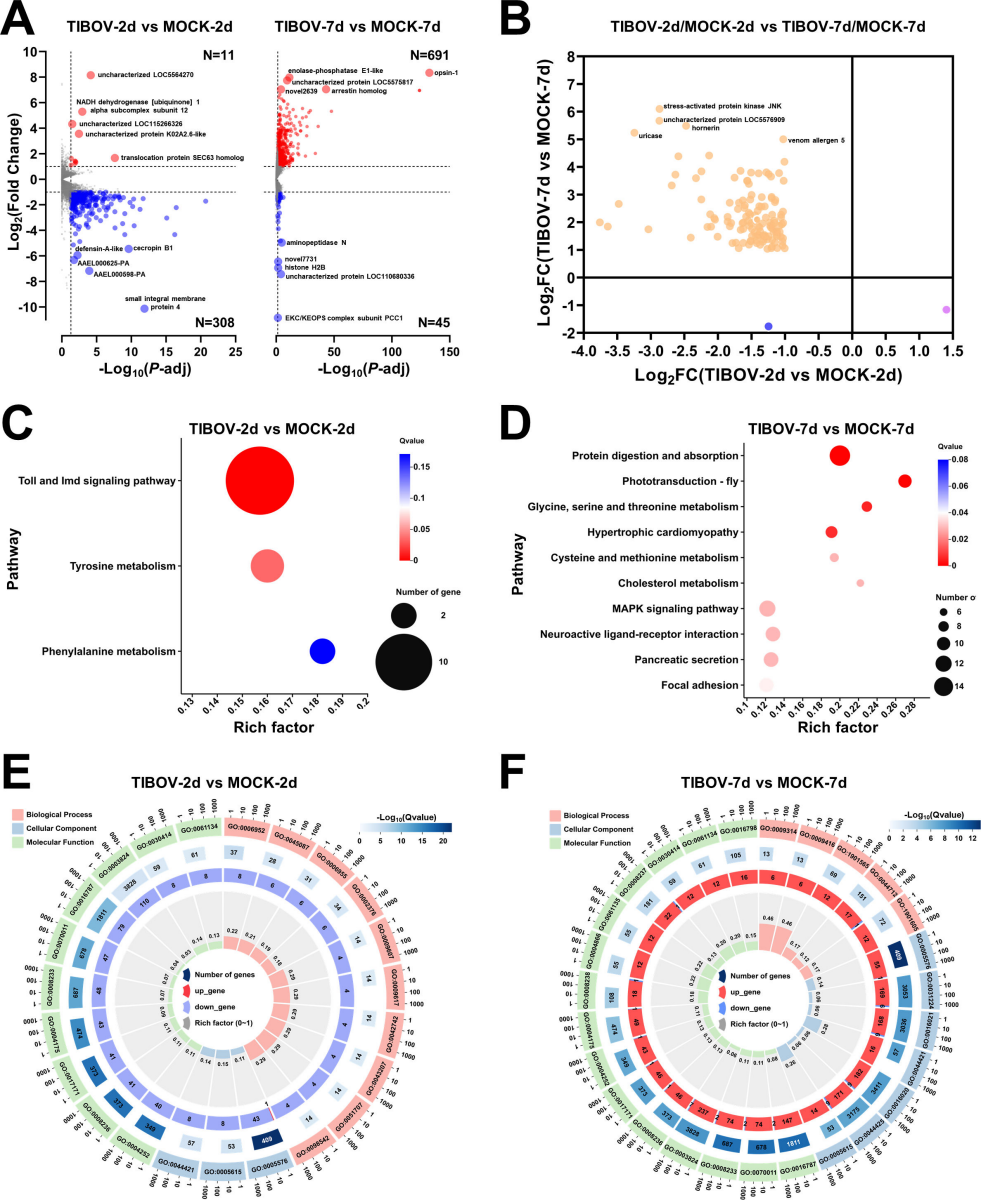
TIBOV impacted gene expression in the oral-infected mosquitoes. (**A**) Significantly upregulated (red) and downregulated (blue) mosquito genes from TIBOV-infected mosquitoes compared with mock-infected mosquitoes at 2 and 7 dpi. (**B**) A scatter plot of expression changes displayed the genes that consistently had differential expression in TIBOV-infected mosquitoes at 2 and 7 dpi. In panels **C** and **D**, respectively, KEGG pathway analysis of DEGs at 2 and 7 dpi was displayed. In panels **E** and **F**, respectively, enriched GO categories linked to DEGs at 2 and 7 dpi were shown when *Q* value ≤ 0.0001.

To figure out the molecular interactions of TIBOV with *Ae. aegypti*, RNA sequencing was used to examine the overall alterations in the *Ae. aegypti* transcriptome after infection. Analysis of mRNA expression profiles of *Ae. aegypti* at the different dpi revealed that the amount of differentially expressed genes (DEGs) at 7 dpi (total DEGs were 736: 691 upregulated genes and 45 downregulated genes) were much greater than that at 2 dpi (total DEGs were 319: 11 upregulated genes and 308 downregulated genes) [*P*-adj ≤ 0.05 and |log_2_(fold change) | ≥ 1] (Fig. 4A).

The number of upregulated genes was significantly lower than the number of downregulated genes in the blood-feeding infected mosquitoes at 2 dpi (Fig. 4A; Table S1 ). Among the top five upregulated genes, only two genes had the functional annotation: NADH dehydrogenase [ubiquinone] 1 alpha subcomplex subunit 12 (*gene-LOC110681452*) and translocation protein SEC63 homolog (*gene-LOC5569901*) (Fig. 4A). Among the downregulated genes, numerous genes were related to cellular immunity, such as peptidoglycan-recognition protein 2 (*gene-LOC5571998*), stress-activated protein kinase JNK (*gene-LOC5570856*), transcription factor AP-1 (*gene-LOC5578266*), serine protease Persephone (*gene-LOC5564988*), serine protease Easter (*gene-LOC23687423*), melanization protease 1 (*gene-LOC5568362*), and seven antimicrobial peptides (AAEL000598-PA, AAEL000625-PA, defensin-A-like, cecropin B1, cecropin N, putative defense protein 1 and attacin-B) (Fig. 4A; Table S1).

The number of upregulated genes was noticeably greater than the number of downregulated genes in the mosquitoes fed with infected blood at 7 dpi (Fig. 4A; Table S2). Three genes of the top five upregulated genes had the functional annotation: Opsin-1 (*gene-LOC5567680*), enolase-phosphatase E1-like (*gene-LOC5567790*), and arrestin homolog (*gene-LOC5577143*) (Fig. 4A). Additionally, the other four opsin genes (*gene-LOC5568060*, *gene-LOC110680887*, *gene-LOC5576882*, and *gene-LOC5572198*) also dramatically increased their transcripts, with the upregulated multiple of *gene-LOC5567680* shown to be the highest (Table S2). Opsins are proteins that bind to light-reactive substances to support circadian cycles, vision, phototaxis, and other light-mediated reactions in living organisms (28). Enolase-phosphatase E1 is a bifunctional enzyme exhibiting both phosphatase and atypical enolase activities, which is involved in the methionine salvage pathway (29). Arrestins are ubiquitous regulators of G-protein-coupled receptors (30). Moreover, β-arrestin 2 has been verified to promote virus-induced production of IFN-β and clearance of viruses in macrophages (31). Among the top five downregulated genes, three genes had the predicted functional annotation: EKC/KEOPS complex subunit PCC1 (*gene-LOC5577749*), histone H2B (*gene-LOC110679788*) and aminopeptidase N (*gene-LOC110680884*) (Fig. 4A).

Statistical comparisons of DEGs between the infected mosquitoes at 2 and 7 dpi revealed that 131 genes continuously exhibited differential expression. Only the transcripts of *gene-LOC5576721* (COX assembly mitochondrial protein 2 homolog) were consistently decreasing. The transcripts of novel5083 were upregulated at 2 dpi and then downregulated at 7 dpi. The remaining genes were first downregulated and then upregulated, especially the genes including stress-activated protein kinase JNK, hornerin, uricase, and venom allergen 5 (Fig. 4B; Table S3).

### Variations in immune-related and metabolism-related processes in TIBOV-infected *Ae. aegypti*

To clarify the biological processes and pathways in the TIBOV-infected *Ae. aegypti* at 2 and 7 dpi, KEGG pathway and GO enrichment analyses were carried out. Genes were particularly enriched in three pathways at 2 dpi, including the Toll and Imd signaling pathways and Tyrosine metabolism, according to KEGG pathway results (Fig. 4C). At 7 dpi, the enrichment pathways focused mostly on metabolism, such as protein digestion and absorption, phototransduction-fly, and glycine, serine, and threonine metabolism (Fig. 4D). Moreover, GO analysis results revealed that processes connected to the immune system and metabolism were considerably enriched at 2 and 7 days, respectively (Fig. 4E and F; Tables S4 and S5).

## DISCUSSION

Studies on vector competence enhance our knowledge of arbovirus transmission, including how the virus infects the vector and the cycles between the arthropod vector and the vertebrate host. This study focused on evaluating the vector susceptibility of two mosquito species (*Ae. aegypti* and *Cx. p. pallens*) to TIBOV and investigating the immune response of *Ae. aegypti* to TIBOV.

While viral titers have not been reported in natural or experimental infected animals for TIBOV, previous reports on BTV-infected IFNAR^−/−^ mice indicated viral titer was about 10^4.8^ PFU/mL concentration in the blood (32). In a similar mice model for EHDV, the viral titer reached 10^5.8^ TCID_50_/mL in the spleens of infected mice (33). In connection with the infection titers used by other orbiviruses to infect *Culicoides*, the titers can range from 10^2.94^ to 10^6.9^ PFUe/mL (26, 27, 34, 35). Based on those data, we chose the dose of 10^3^–10^6^ PFU/mL in this study. Despite effective midgut infection being established when using the infection titer of 10^6^ PFU/mL, we observed low dissemination and transmission rates when compared to the vector competence studies of *Culicoides* to other orbiviruses (34, 36). This suggests that TIBOV is capable of replicating in the midgut but faces a challenge in spreading into or replicating in salivary glands in both *Ae. aegypti* and *Cx. p. pallens*. Since midgut and salivary gland barriers were reported to play crucial roles in limiting the virus dissemination and replication (37, 38), the challenges in TIBOV’s spread may be due to these barriers. When the offsprings (eggs, larvae, pupae, and adults) of TIBOV-positive *Ae. aegypti* were analyzed, only some of the eggs were detected as positive for the viral RNA (Fig. S1). But the sample size for the offspring is very small, so more testing should be done to confirm the results. In addition, only the *Ae. aegypti* Rockefeller strain and the *Cx. p. pallens* Beijing strain were assessed here, and these colonies are lab-adapted, which may not be indicative of wild mosquitoes’ susceptibility. Moreover, laboratory experimental infection data alone are not sufficient to evaluate the risk of TIBOV transmission by mosquitoes, density of mosquitoes and host-vector contact dynamics also play roles in the transmission of mosquito-borne viruses (39).

The vector competence of mosquitoes and *Culicoides* to orbiviruses is different. For example, it has been reported that several *Culicoides* species were confirmed vectors of BTV and EHDV (34, 40). *Cx. pipiens* and *Ae. albopictus* were found not susceptible to BTV infection (41), and this is the only study dealing with mosquito vector capacity on orbiviruses. Further studies on additional species and geographic strains of mosquitoes and other vectors (such as *Culicoides*) should be conducted to identify the primary vectors for TIBOV. So far, the infectivity and pathogenicity of the BTV in IFNAR^−/−^ mice and sheep have been evaluated (42), but there is very limited information on TIBOV infection using animal models. Thus, further animal infection studies of TIBOV need to be carried out, as well as arthropod-TIBOV-vertebrate transmission studies should also be conducted.

The transcriptome investigation of TIBOV-infected *Ae. aegypti* revealed that DEGs at 2 dpi were significantly enriched in the Toll and Imd signaling pathways by KEGG pathway analyses and defense response by GO enrichment analyses (Fig. 4C and E; Table S4). At this time point, the mean viral RNA copies of TIBOV in *Ae. aegypti* was 10^3.95^ viral RNA copies/μL, lower than that at 7 dpi (10^5.21^ viral RNA copies/μL) (Fig. S2). It was indicated that the Toll and Imd signaling pathways may be related to the mosquito’s defense against TIBOV at the early phase. In arbovirus-infected mosquitoes, such as those *Ae. aegypti* infected with dengue virus (DENV), the Toll pathway has been found to have an antiviral function (43, 44). Silencing specific defensins or cecropins has also been found to enhance DENV replication in *Ae. aegypti* mosquitoes (45). In TIBOV-infected *Ae. aegypti* at 2 dpi, many antimicrobial peptides downstream of the Toll and Imd signaling pathways were significantly downregulated (Table S1), suggesting that TIBOV suppressed *Ae. aegypti*’s defense to increase the virus replication in mosquitoes. The majority of DEGs at 7 dpi were upregulated and were significantly enriched in protein digestion and absorption (Fig. 4D). The mosquito likely raised its metabolism to compensate for the loss of defense against virus infection. Additionally, some genes were also enriched in immune-related pathways, such as the MAPK signaling pathway (Fig. 4D). Heat shock protein 70 A1 (*gene-LOC110674150* and *gene-LOC110674151*), transcription factor AP-1 (*gene-LOC5578266*), and stress-activated protein kinase JNK (*gene-LOC5570856*) in this pathway were upregulated in TIBOV-infected *Ae. aegypti* at 7 dpi and downregulated at 2 dpi (Table S3). Stress-activated protein kinase JNK, a crucial component of the c-Jun N-terminal kinase (JNK) pathway, one of the main signaling cassettes of the MAPK signaling pathway, showed the greatest upregulation at 7 dpi (fold change: 6.10) (Fig. 4B; Table S2). Moreover, in the arbovirus infection of *Ae. aegypti*, the JNK pathway showed a broad antiviral function against dengue virus, zika virus, and chikungunya virus in salivary glands and midguts, and this pathway produced consistent responses for each virus (46). For TIBOV-infected *Ae. aegypti*, the JNK pathway might be involved in the infected mosquitoes’ defense against viral dissemination to the other tissues, such as salivary glands. In conclusion, the Toll and Imd signaling pathways, particularly the MAPK signaling pathway, may be crucial in the interaction between TIBOV and *Ae. aegypti.*

In conclusion, both *Ae. aegypti* and *Cx. p. pallens* exhibited weak vector competence for TIBOV. Further studies on the competence of different vector species in transmitting this novel and neglected arbovirus are crucial for enhancing public health preparedness.

## MATERIALS AND METHODS

### Mosquito strains and rearing

*Ae. aegypti* (Rockefeller strain) were acquired from the Laboratory of Tropical Veterinary Medicine and Vector Biology at Hainan University, and *Culex pipiens pallens* (Beijing strain) were from the Department of Vector Biology and Control, National Institute for Communicable Disease Control and Prevention, China CDC. The eggs and larvae were reared under optimum conditions that were set at 28°C, with a relative humidity of 70%–80% and a light-dark cycle of 12:12 h each to ensure consistent adult size. Adult mosquitoes were fed with 8% glucose solution and maintained in mesh cages (30 × 30 × 30 cm) within incubators set at 28°C, under a relative humidity of 80% and light-dark cycle of 12:12 h. Mosquitoes were reared in an arthropod containment level 1 laboratory as described previously (47).

### Viral stock

TIBOV (strain YN15-283-01) was isolated from *Culicoides* previously collected in Xishuangbanna, Yunnan Province, China, in 2015 (22). The working stock of TIBOV used was taken from the sixth passage in BHK-21 cell lines, and the titer read 1.6 × 10^7^ plaque-forming units mL^−1^ was determined through the plaque assay.

### Mosquito infection through blood-feeding

Before infection, adult female mosquitoes (5-day-old) were starved for 12 h. TIBOV mixed with defibrated horse blood (ChunduBio) with the final viral concentrations of 10^6^, 10^5^, 10^4^, and 10^3^ PFU/mL were provided to the starved mosquitoes through an artificial mosquito feeding system (Hemotek) with parafilm serving as a membrane to cover the blood. After 1 h of blood feeding, fully engorged mosquitoes were transferred to new containers and reared in an incubator at 28°C, under 80% RH with a 12/12-h L/D cycle. An 8% glucose solution on cotton pads was supplied to the mosquitoes until the time of use. Mosquito infection was conducted in the arthropod containment level 2 laboratory aiming at the succeeding targets.

1. To investigate the minimum infection concentration of TIBOV in *Ae. aegypti* and *Cx. p. pallens*, the infected mosquitoes with infection titers 10^6^–10^3^ PFU/mL were subsequently harvested at 14 dpi and were subjected to viral RNA determination.
2. To evaluate whether TIBOV could effectively infect *Ae. aegypti* and *Cx. p. pallens*, a high concentration (10^6^ PFU/mL) was chosen. Infected mosquitoes were subsequently collected at 4, 7, 10, and 14 dpi for viral RNA determination.
3. To determine the distribution of TIBOV in infected mosquitoes, 10^6^ PFU/mL TIBOV titer of blood was fed, and the presence of viral RNA in midguts, heads, and saliva of mosquitoes at 7, 9, 11, and 13 dpi was examined. To collect saliva, the legs and wings of mosquitoes were cut away, and the proboscises were inserted into 10 µL pipette tips containing 2 µL of Immersion Oil Type B (Cargille) for 1 h as previously described (47). After collecting saliva, different mosquito tissues were examined under a dissecting microscope. All mosquito tissues and saliva were put into tubes with 200 µL RPMI 1640 supplemented with 2% penicillin/streptomycin/gentamicin Solution and stored at −80°C until further processing.

Vector competence of the mosquitoes was evaluated by calculating the infection rate (number of positive midguts/the total number of mosquitoes tested), dissemination rate (number of infected heads/the number of infected midgut), transmission rate (number of infected saliva/the number of infected midgut) (47), and transmission efficiency (number of infected saliva/the total number of mosquitoes tested).

The figure introductory panels (Fig. 1A, D, and 2A; Fig. S1) were created with BioRender.com (https://www.biorender.com/), and the corresponding authorization for publication had been granted.

### Evaluation of viral replication by qRT-PCR

All samples were initially homogenized using a Low-Temperature Tissue Homogenizer Grinding Machine (Servicebio) (operating frequency = 60 Hz, operation time = 15 s, pause time = 10 s, cycles = 2, and setting temperature = 4°C), followed by centrifugation for 10 min at 12,000 × *g* min and 4°C. The total RNA (80 µL) of each sample was extracted using an automated nucleic acid extraction system following the manufacturer’s instructions (NanoMagBio).

The viral RNA copies in each sample were tested by absolute quantification through one-step qRT-PCR with a standard curve. CFX96 Touch Real-Time PCR Detection System (Bio-Rad) and Luna Universal Probe One-Step RT-qPCR Kit (NEB) were used. The primers for qRT-PCR targeted the VP1 (Segment 1) of TIBOV, including TBV-VP1-F (5′-CTCTCTCCGAAGTAAGATATTCCG-3′), TBV-VP1-R (5′-TGTGCTTGACCAACTAGGG-3′), and TBV-VP1-Probe (5′FAM-AGTCAAATCTGAGGCCGTGTGACT-BHQ13′). The cutoff for TIBOV-positive samples was set at Ct < 35. The positive cutoff value was evaluated by comparing serial 10-fold dilutions either inoculated on cells or assayed via qRT-PCR (Table S6). The equation for the standard curve [*y* = −3.8894*x* + 43.767, *x* = lg (TIBOV RNA copies/μL), *y* = Ct value, *R*^2^ = 0.9978] was used to calculate the TIBOV RNA copies/μL in each sample, which was generated using 10-fold serial dilutions of transcribed RNA *in vitro* (10^11.7^ copies/μL) (Fig. S3).

### TIBOV detection in mosquitoes by immunofluorescence assay

Midguts from infected mosquitoes were dissected (*n* = 30) at 14 dpi. The tissues were fixed using 4% paraformaldehyde for 1 h and washed with PBS containing 0.3% Triton X-100 (PBST) five times. Next, tissues were placed in blocking solution (PBS containing 5% goat serum and 0.3% Triton X-100) for 1 h and then incubated with primary rabbit anti-TIBOV-VP7 antibody (derived from rabbit serum, diluted 1:200 in PBST containing 5% goat serum) for 24 h, followed by secondary Cy3-conjugated goat anti-rabbit IgG (diluted 1:250 in PBST containing 5% goat serum; Abcam) for 12 h. The actin cytoskeleton was stained with Alexa Fluor 488 Phalloidin (Invitrogen) for 1 h. After each step, tissues were washed at least five times in 0.3% PBST to prevent the effects of reagents from affecting subsequent operations. Finally, tissues were mounted onto slides using SlowFade Diamond Antifade Mountant (Invitrogen), and images were recorded through a Leica SP8 confocal microscope (filter information TD 458/514/561, Leica, Germany). Using LAS X software (Leica), z-stack images were merged, and scale bars were added. PowerPoint 2019 was utilized for image grouping. All samples were analyzed under the same microscope and software settings.

### TIBOV visualization in mosquitoes by transmission electron microscopy

Midguts from TIBOV-infected mosquitoes (7 dpi) were dissected and then fixed in 2.5% glutaraldehyde. Ultrathin sections for fixed midgut were cut in an ultramicrotome and were stained using 2% uranium acetate saturated solution and lead citrate. Examinations were made in Tecnai G20 TWIN transmission electron microscope (FEI, United States) at 200 kV. Sample handling and observation were done at the Center for Instrumental Analysis and Metrology (Wuhan Institute of Virology, China) as described previously (47).

### Transcriptome profile after *Ae. aegypti* infected with TIBOV

*Ae. aegypti* were collected at 2 and 7 dpi after being fed with TIBOV-blood or mock-blood. RNAs for 10 TIBOV-positive mosquitoes were mixed together as one sample for transcriptome sequencing. Three independent biological replicates were performed. The samples were sent to Wuhan Benagen Tech Solutions Company for data processing and commercial RNA-seq services. RNA degradation and contamination were monitored on 1% agarose gels. The NanoPhotometer spectrophotometer (Implen, CA, USA) was used to determine the purity of RNA. RNA integrity was evaluated using the RNA Nano 6000 Assay Kit of the Bioanalyzer 2100 system (Agilent Technologies, CA, USA). Using the NEBNext UltraTM RNA Library Prep Kit for Illumina (NEB, USA), sequencing libraries were created in accordance with the manufacturer’s instructions, and index codes were added to attribute sequences to each sample. According to the manufacturer’s instructions, the TruSeq PE Cluster Kit v3-cBot-HS (Illumina) was used to cluster the index-coded sample data on a cBot Cluster Generation System. The library preparations were sequenced on an Illumina Novaseq platform after cluster creation, producing 150-bp paired-end reads. Clean reads were produced by eliminating reads containing adapter, reads containing ploy-N, and low-quality reads from raw data. With the help of the Trinity software (http://trinityrnaseq.sourceforge.net/), *de novo* assembly of the clean reads was performed. Clean reads were mapped at the *Ae. aegypti* genome database (RefSeq: GCF_002204515.2). To find protein functional annotations based on sequence similarity, the unigene sequences of the samples were searched using BLASTX against the Nr, KEGG, and GO databases (*E*-value ≤ 1E-5). The changes in gene expression between several samples were directly compared using the Fragments Per Kilobase of exon model per Million mapped fragments (FPKM) values. For finding differentially expressed genes, the “base mean” value was calculated using the DESeq software (48). The absolute value of log_2_ ratio ≥ 1 and *P*-adj ≤ 0.05 were set as the thresholds for the significance of the gene expression difference between the two samples. Volcano plots were drawn by GraphPad Prism statistical software 9.5.0. Heatmaps, bubble diagrams, and concentric circle diagrams were drawn using the online software ChiPlot (https://www.chiplot.online/).

### Statistical analysis

GraphPad Prism software was used to analyze the collected experimental data. Significant differences among variables obtained from mosquito infection, dissemination, and transmission were analyzed using the non-parametric Mann-Whitney test for multiple comparisons and Fisher’s exact test where appropriate, as specified in the figure legends. *P* ≤ 0.05 was considered statistically significant.

## SUPPLEMENTAL MATERIAL

Supplemental figures and tables msphere.00062-24-s0001.pdf Fig. S1 to S4 and Table S1 to S6.

ASM does not own the copyrights to Supplemental Material that may be linked to, or accessed through, an article. The authors have granted ASM a non-exclusive, world-wide license to publish the Supplemental Material files. Please contact the corresponding author directly for reuse.
