# Supplementary material for: Evaluation of vector susceptibility in Aedes aegypti and Culex pipiens pallens to Tibet orbivirus
Source: mSphere. 2024 Mar 26;9(4):e00062-24. doi: 10.1128/msphere.00062-24 (PMC11036799; doi:10.1128/msphere.00062-24)
Supplement: Supplemental figures and tables — Fig. S1 to S4 and Table S1 to S6. [file msphere.00062-24-s0001.pdf]

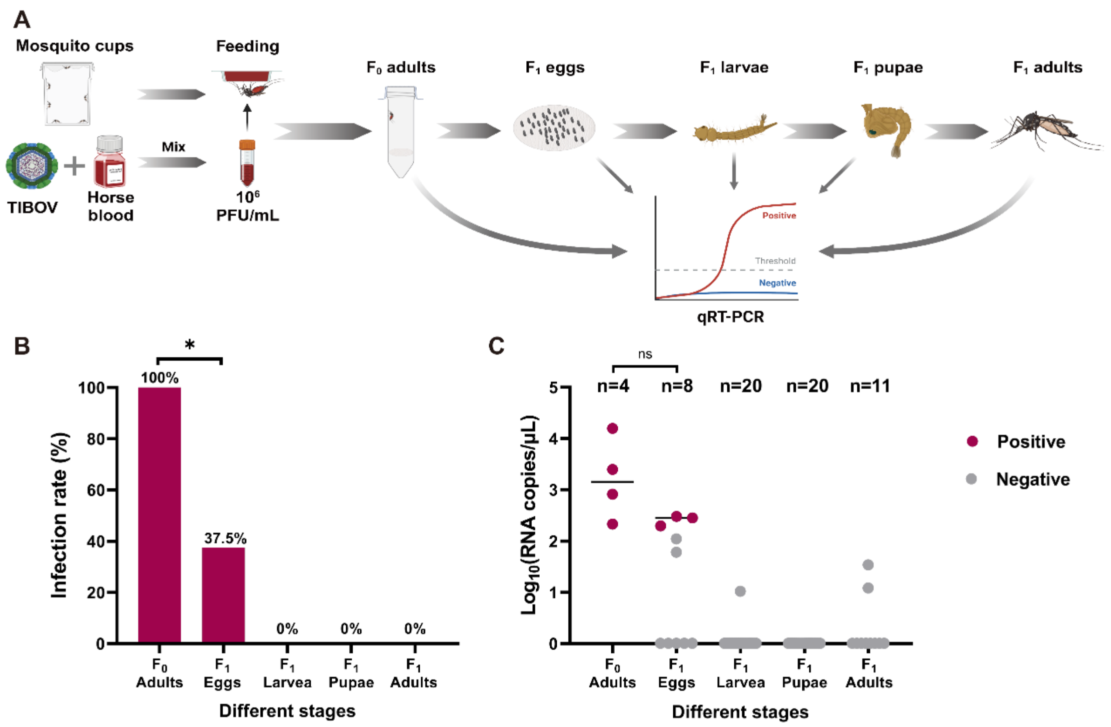


**Figure S1. Vertical transmission of TIBOV in *Ae. aegypti*.**

(A) After feeding with blood meal containing $10^6$ PFU/mL TIBOV, fifty fully engorged female *Ae. aegypti* ($F_0$ adults) were collected in a new container with a wet paper towel for spawning. After about 7 days, four $F_0$ adults that were still alive were tested, and the paper towel with eggs were cut into several pieces, eight pieces (50-100 eggs/piece) used for test ($F_1$ eggs) and the remaining were hatched. Then at different stages, including $F_1$ larvae, $F_1$ pupae and $F_1$ adults, some of them were tested. (B) and (C) infection rates and viral RNA copies in different stages. Each dot represents an individual mosquito, and the gray dot stands for the negative sample with Ct value > 35

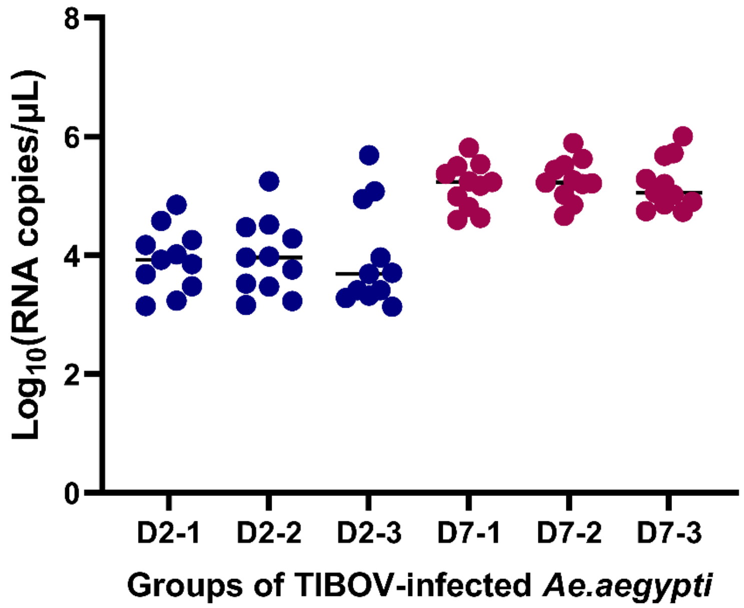


**Figure S2. Viral RNA copies of TIBOV in *Ae. aegypti* at 2 dpi and 7 dpi**

Each dot represents an individual mosquito, and the blue dot stands for the sample from 2 dpi, and the purple dot stands for the sample from 7 dpi.

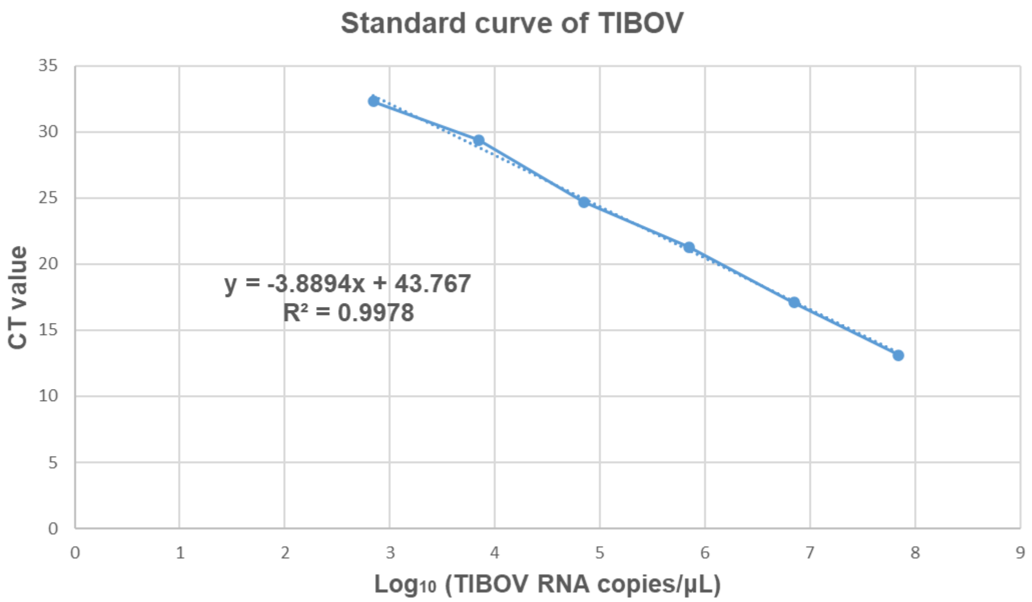


**Figure S3. The standard curve of TIBOV RNA copies/μL and Ct values.**

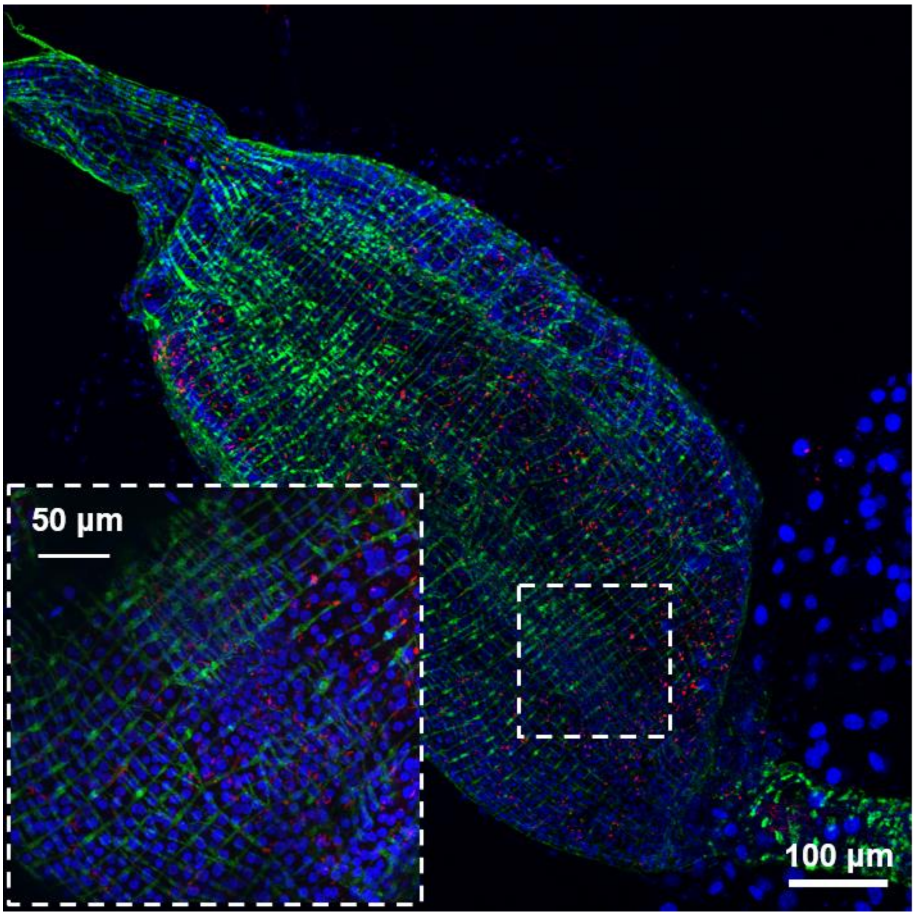


**Figure S4. Additional picture of immunohistochemical visualization of TIBOV antigen in *Ae. aegypti* midguts.**

**Table S1. Differentially expressed genes in the mosquitoes following feeding on TIBOV-blood comparing to those feeding on the mock-blood at 7 dpi. The downregulated genes were highlighted in blue, whereas the upregulated genes were highlighted orange. Aae-BV-2d-1, 2 and 3 mean TIBOV-infected *Ae. aegypti* at 2 dpi, and Aae-BC-2d-1, 2 and 3 mean Mock-infected *Ae. aegypti* at 2 dpi.**

| Gene_name | NR annotation | $Log_2$(FoldChange) | *P*- adj | FPKM of gene | | | | | |
|---|---|---|---|---|---|---|---|---|---|
| | | | | Aae-BV-2d-1 | Aae-BV-2d-2 | Aae-BV-2d-3 | Aae-BC-2d-1 | Aae-BC-2d-2 | Aae-BC-2d-3 |
| gene-LOC5564270 | uncharacterized LOC5564270 | 8.15 | 0.00 | 0.27 | 1.91 | 6.06 | 0.00 | 0.00 | 0.00 |
| gene-LOC11068145[illegible] | NADH dehydrogenase [ubiquinone] 1 alpha subcomplex subunit 12 | 5.30 | 0.00 | 16.26 | 49.82 | 32.44 | 0.00 | 0.00 | 2.64 |
| novel124 | uncharacterized LOC115266326 | 4.33 | 0.03 | 20.49 | 1.70 | 111.24 | 1.80 | 1.40 | 3.58 |
| novel552 | uncharacterized protein K02A2.6-like | 3.56 | 0.00 | 1.31 | 0.36 | 0.46 | 0.01 | 0.04 | 0.13 |
| gene-LOC5569901 | translocation protein SEC63 homolog | 1.66 | 0.00 | 5.11 | 6.31 | 7.84 | 2.34 | 1.64 | 2.16 |
| gene-LOC5569051 | cyclic nucleotide-gated cation channel subunit A | 1.64 | 0.04 | 2.65 | 1.78 | 2.13 | 1.31 | 0.17 | 0.64 |
| gene-LOC5568759 | LIM/homeobox protein Lhx3-like | 1.43 | 0.01 | 3.14 | 5.89 | 4.99 | 1.24 | 2.37 | 1.43 |
| novel5083 | - | 1.40 | 0.01 | 56.89 | 82.56 | 47.77 | 21.07 | 29.92 | 13.09 |
| gene-LOC5572170 | protein obstructor-E | 1.34 | 0.01 | 10.60 | 17.09 | 10.73 | 4.48 | 7.23 | 3.48 |
| gene-LOC11067561[illegible] | chromobox protein homolog 1-like | 1.24 | 0.01 | 23.00 | 18.57 | 33.37 | 12.53 | 8.62 | 11.87 |
| gene-LOC5572337 | zinc finger protein Xfin | 1.11 | 0.05 | 19.35 | 6.75 | 17.97 | 5.85 | 7.94 | 6.67 |
| gene-LOC11068081[illegible] | small integral membrane protein 4 | -10.14 | 0.00 | 0.00 | 0.00 | 0.00 | 52.37 | 24.53 | 28.73 |
| novel364 | AAEL000598-PA | -7.17 | 0.00 | 0.00 | 0.37 | 0.00 | 23.85 | 23.92 | 3.94 |
| novel365 | AAEL000625-PA | -6.32 | 0.02 | 1.10 | 0.00 | 0.77 | 133.53 | 21.82 | 1.03 |
| gene-LOC5579095 | defensin-A-like | -5.94 | 0.01 | 0.82 | 0.99 | 2.61 | 273.30 | 14.59 | 4.31 |
| novel67 | cecropin B1 | -5.45 | 0.00 | 0.31 | 2.12 | 0.23 | 40.02 | 50.08 | 20.41 |

| | | | | | | | | | |
|---|---|---|---|---|---|---|---|---|---|
| novel5352 | uncharacterized protein K02A2.6-like | -5.14 | 0.02 | 0.00 | 0.00 | 0.11 | 0.83 | 3.61 | 0.03 |
| gene-LOC5566785 | perlucin | -4.87 | 0.00 | 0.53 | 0.90 | 0.53 | 38.84 | 18.29 | 1.03 |
| novel363 | cecropin N; hypothetical protein DI617_09265 | -4.34 | 0.00 | 2.29 | 5.84 | 1.64 | 129.47 | 163.09 | 36.16 |
| gene-LOC11067700[illegible] | microfibril-associated glycoprotein 4-like | -4.34 | 0.00 | 0.65 | 0.84 | 0.59 | 29.12 | 8.62 | 4.89 |
| gene-LOC11067862[illegible] | uncharacterized protein LOC110678629 | -4.28 | 0.01 | 0.04 | 0.04 | 0.15 | 1.28 | 3.07 | 0.16 |
| gene-LOC11067833[illegible] | mucin-5AC isoform X1/X2 | -3.90 | 0.00 | 0.08 | 0.01 | 0.01 | 0.66 | 0.17 | 0.66 |
| gene-LOC5566508 | melanization protease 1 | -3.81 | 0.00 | 1.11 | 0.57 | 0.42 | 12.82 | 12.78 | 3.82 |
| gene-LOC5568623 | uncharacterized protein LOC5568623 | -3.76 | 0.00 | 0.75 | 0.12 | 0.68 | 14.18 | 5.14 | 2.08 |
| gene-LOC5575674 | testisin | -3.70 | 0.00 | 0.20 | 0.10 | 0.08 | 2.55 | 1.33 | 0.81 |
| gene-LOC5572428 | AAEL001794-PA/PB, partial; CD109 antigen | -3.64 | 0.00 | 5.64 | 4.30 | 3.30 | 108.19 | 82.90 | 46.24 |
| gene-LOC5568069 | DNA-binding protein D-ETS-6 isoform X1; protein FEV isoform X2 | -3.61 | 0.01 | 0.12 | 0.12 | 0.56 | 0.70 | 5.74 | 1.38 |
| gene-LOC5569955 | uncharacterized protein | -3.59 | 0.00 | 0.15 | 0.57 | 0.27 | 5.35 | 5.55 | 1.10 |
| gene-LOC5576463 | uncharacterized protein | -3.49 | 0.02 | 0.17 | 0.80 | 1.62 | 25.80 | 3.23 | 2.25 |
| gene-LOC5573649 | uncharacterized protein | -3.48 | 0.00 | 3.63 | 1.08 | 1.00 | 28.35 | 23.25 | 12.43 |
| gene-LOC11067947[illegible] | uncharacterized protein | -3.48 | 0.00 | 0.47 | 0.28 | 0.23 | 6.89 | 0.72 | 2.74 |
| gene-LOC5570298 | pancreatic lipase-related protein 2 | -3.29 | 0.04 | 0.56 | 0.22 | 0.05 | 6.54 | 1.15 | 0.55 |
| gene-LOC5573896 | uricase | -3.25 | 0.00 | 3.03 | 1.61 | 1.34 | 36.90 | 14.90 | 5.49 |
| gene-LOC5572918 | putative defense protein 1 | -3.17 | 0.00 | 1.06 | 0.98 | 0.44 | 13.75 | 6.85 | 2.67 |
| gene-LOC5578028 | attacin-B | -3.10 | 0.04 | 1.26 | 0.40 | 0.22 | 12.06 | 3.59 | 0.49 |
| gene-LOC11067982[illegible] | keratin, type II cytoskeletal 68 kDa, | -3.06 | 0.04 | 7.03 | 1.87 | 5.57 | 43.59 | 76.09 | 5.24 |
| gene-LOC23687443 | AAEL017023-PA, partial; CD109 | -3.04 | 0.00 | 2.15 | 1.08 | 1.14 | 16.90 | 12.50 | 7.29 |
| gene-LOC23687559 | uncharacterized protein | -3.00 | 0.00 | 2.36 | 1.28 | 0.54 | 9.79 | 15.55 | 7.73 |
| gene-LOC5564288 | collectin-11 | -2.94 | 0.02 | 4.31 | 2.73 | 0.54 | 44.25 | 12.71 | 2.92 |
| gene-LOC5576909 | uncharacterized protein | -2.87 | 0.00 | 1.33 | 0.77 | 1.69 | 14.79 | 8.18 | 5.51 |
| gene-LOC5570856 | stress-activated protein kinase JNK | -2.87 | 0.00 | 0.60 | 0.65 | 1.05 | 4.24 | 8.85 | 3.81 |
| gene-LOC5569732 | ornithine decarboxylase | -2.87 | 0.01 | 0.22 | 0.51 | 0.12 | 2.10 | 3.04 | 1.07 |
| gene-LOC5569108 | uncharacterized protein | -2.81 | 0.04 | 2.47 | 1.01 | 0.55 | 14.16 | 16.79 | 2.25 |
| gene-LOC5569890 | AAEL007993-PA; serine protease | -2.78 | 0.00 | 0.36 | 0.11 | 0.39 | 2.29 | 3.50 | 1.07 |
| gene-LOC5564201 | serine protease easter | -2.73 | 0.00 | 1.80 | 0.88 | 0.75 | 7.62 | 11.73 | 3.36 |
| gene-LOC5579410 | serine protease easter | -2.69 | 0.00 | 1.47 | 1.09 | 1.59 | 10.25 | 9.13 | 4.80 |
| gene-LOC5565977 | AAEL005093-PA; serine protease | -2.67 | 0.00 | 2.22 | 1.08 | 1.29 | 16.00 | 8.46 | 4.94 |

| | | | | | | | | | |
|---|---|---|---|---|---|---|---|---|---|
| gene-LOC5569107 | uncharacterized protein | -2.63 | 0.00 | 6.70 | 5.70 | 2.23 | 34.87 | 41.32 | 9.55 |
| gene-LOC11067596 | uncharacterized protein | -2.61 | 0.01 | 0.19 | 0.16 | 0.20 | 0.63 | 1.92 | 0.76 |
| gene-LOC23687564 | glycine-rich protein 5 | -2.59 | 0.00 | 22.75 | 8.52 | 9.01 | 124.70 | 80.52 | 37.98 |
| gene-LOC5575350 | tryptase | -2.56 | 0.01 | 3.12 | 2.16 | 0.33 | 20.63 | 8.59 | 3.93 |
| gene-LOC11068028 | LOW QUALITY PROTEIN: uncharacterized protein | -2.54 | 0.00 | 0.12 | 0.09 | 0.10 | 0.63 | 0.62 | 0.52 |
| gene-LOC5577595 | putative protein TPRXL isoform | -2.51 | 0.01 | 0.27 | 0.21 | 0.52 | 1.89 | 1.64 | 2.19 |
| gene-LOC5571242 | hornerin | -2.47 | 0.00 | 6.90 | 2.47 | 2.08 | 30.14 | 25.68 | 7.49 |
| gene-LOC5576723 | LOW QUALITY PROTEIN: | -2.43 | 0.00 | 4.04 | 1.96 | 1.72 | 12.43 | 18.93 | 10.11 |
| gene-LOC5578691 | serine protease easter | -2.41 | 0.01 | 0.46 | 0.52 | 0.89 | 4.82 | 3.94 | 1.19 |
| gene-LOC5572108 | uncharacterized protein | -2.40 | 0.00 | 5.09 | 3.65 | 2.67 | 24.25 | 28.87 | 7.57 |
| gene-LOC5572814 | uncharacterized protein | -2.36 | 0.03 | 0.73 | 0.63 | 0.58 | 3.62 | 4.96 | 1.34 |
| gene-LOC5566672 | synaptic vesicle glycoprotein 2B | -2.35 | 0.00 | 1.82 | 1.14 | 1.70 | 8.66 | 6.50 | 8.84 |
| gene-LOC5575814 | uncharacterized protein | -2.33 | 0.00 | 5.36 | 3.40 | 1.40 | 22.39 | 19.73 | 9.09 |
| gene-LOC5576908 | uncharacterized protein | -2.30 | 0.01 | 3.73 | 1.23 | 1.34 | 12.59 | 14.79 | 3.47 |
| gene-LOC5563808 | serine protease inhibitor 28Dc | -2.29 | 0.00 | 2.18 | 0.78 | 0.94 | 5.25 | 10.47 | 3.34 |
| gene-LOC5579424 | uncharacterized protein | -2.28 | 0.03 | 1.60 | 1.13 | 4.87 | 23.72 | 6.10 | 8.42 |
| gene-LOC11067982 | glycine-rich protein DOT1-like | -2.27 | 0.03 | 4.80 | 5.17 | 3.91 | 15.42 | 34.49 | 9.83 |
| gene-LOC5563617 | serine protease easter | -2.25 | 0.00 | 3.52 | 2.96 | 2.60 | 17.25 | 17.55 | 8.55 |
| gene-LOC5578510 | trypsin 5G1 | -2.25 | 0.05 | 0.96 | 2.04 | 0.88 | 12.33 | 4.41 | 1.87 |
| novel80 | - | -2.24 | 0.01 | 1.24 | 1.61 | 0.76 | 2.49 | 3.20 | 11.73 |
| gene-LOC5576724 | endochitinase | -2.22 | 0.02 | 0.91 | 1.16 | 0.22 | 3.03 | 2.34 | 8.18 |
| gene-LOC5572149 | apolipoprotein D | -2.22 | 0.00 | 7.17 | 4.93 | 4.41 | 22.51 | 25.57 | 29.12 |
| gene-LOC5567045 | general odorant-binding protein | -2.20 | 0.00 | 0.99 | 0.69 | 0.89 | 3.54 | 4.62 | 3.73 |
| gene-LOC11067802 | uncharacterized protein | -2.19 | 0.00 | 7.53 | 10.14 | 8.95 | 49.43 | 54.02 | 18.99 |
| gene-LOC5571998 | peptidoglycan-recognition protein 2 | -2.17 | 0.01 | 1.92 | 2.73 | 2.29 | 19.40 | 7.80 | 4.58 |
| gene-LOC5569637 | leucine-rich repeat-containing G- | -2.16 | 0.00 | 12.85 | 6.57 | 5.38 | 47.74 | 42.04 | 21.37 |
| gene-LOC5563570 | L-dopachrome tautomerase yellow- | -2.14 | 0.00 | 4.73 | 3.12 | 1.96 | 23.14 | 10.22 | 8.95 |
| gene-LOC5575351 | tryptase | -2.13 | 0.00 | 21.77 | 13.46 | 14.38 | 100.87 | 72.66 | 45.54 |
| gene-LOC5575325 | transmembrane protease serine 2 | -2.12 | 0.00 | 2.78 | 1.81 | 1.46 | 13.60 | 9.09 | 3.53 |
| gene-LOC5578871 | uncharacterized protein | -2.10 | 0.00 | 1.09 | 1.41 | 0.79 | 6.43 | 5.88 | 1.93 |
| gene-LOC5579366 | polyserase-2 | -2.09 | 0.00 | 2.04 | 2.12 | 1.16 | 10.89 | 8.28 | 3.79 |
| gene-LOC5572635 | carboxypeptidase B | -2.08 | 0.00 | 1.68 | 1.12 | 1.58 | 4.06 | 7.67 | 6.91 |
| gene-LOC5578646 | cytochrome P450 4d1 | -2.08 | 0.03 | 0.39 | 0.21 | 0.68 | 1.68 | 2.17 | 1.61 |
| gene-LOC11067696 | annulin-like | -2.06 | 0.02 | 1.04 | 0.53 | 0.61 | 2.59 | 5.40 | 1.00 |

| | | | | | | | | | |
|---|---|---|---|---|---|---|---|---|---|
| gene-LOC11068105[illegible] | annulin-like | -2.06 | 0.02 | 1.04 | 0.53 | 0.61 | 2.59 | 5.40 | 1.00 |
| gene-LOC5576459 | uncharacterized protein | -2.06 | 0.00 | 26.98 | 7.30 | 18.05 | 86.74 | 70.22 | 63.36 |
| gene-LOC5578672 | sodium-dependent nutrient amino acid transporter 1 isoform X1/X2 | -2.05 | 0.00 | 2.60 | 1.72 | 1.76 | 8.20 | 12.02 | 4.86 |
| gene-LOC5576526 | putative transporter SVOPL | -2.02 | 0.00 | 1.36 | 0.88 | 0.67 | 3.90 | 3.27 | 4.62 |
| gene-LOC5572129 | transient receptor potential cation channel protein painless isoform | -2.01 | 0.00 | 1.84 | 1.20 | 0.97 | 5.43 | 7.49 | 3.29 |
| gene-LOC5570871 | angiopoietin-related protein 1 | -2.00 | 0.02 | 1.99 | 1.23 | 2.18 | 13.53 | 6.21 | 2.18 |
| novel2627 | AAEL011675-PA | -1.97 | 0.01 | 0.19 | 0.36 | 0.70 | 1.17 | 2.01 | 1.75 |
| gene-LOC23687800 | glycine-rich protein 23 | -1.96 | 0.03 | 5.31 | 4.57 | 5.76 | 24.01 | 31.89 | 6.89 |
| gene-LOC5565630 | dimethyladenosine transferase 2, mitochondrial; uncharacterized | -1.94 | 0.00 | 9.30 | 6.24 | 4.93 | 27.50 | 33.33 | 20.00 |
| gene-LOC5578909 | fatty acyl-CoA reductase wat | -1.92 | 0.01 | 0.96 | 0.41 | 0.62 | 2.54 | 2.45 | 2.53 |
| gene-LOC5567079 | uncharacterized protein | -1.88 | 0.01 | 0.52 | 0.50 | 0.72 | 2.52 | 2.73 | 1.01 |
| novel519 | - | -1.87 | 0.03 | 1.04 | 3.57 | 3.87 | 6.13 | 17.71 | 7.60 |
| gene-LOC5575326 | suppressor of tumorigenicity 14 protein isoform X1; proclotting | -1.86 | 0.00 | 4.31 | 3.25 | 2.88 | 22.07 | 9.51 | 6.49 |
| gene-LOC5563663 | serine protease easter | -1.86 | 0.00 | 9.71 | 7.74 | 3.40 | 37.84 | 26.47 | 13.86 |
| gene-LOC5571325 | tyrosine aminotransferase | -1.86 | 0.00 | 10.97 | 4.14 | 2.29 | 22.40 | 19.17 | 21.82 |
| gene-LOC5575876 | synaptic vesicle glycoprotein 2B | -1.85 | 0.00 | 1.52 | 1.39 | 1.86 | 7.34 | 8.45 | 3.40 |
| gene-LOC5563856 | uncharacterized protein LOC110676873; AAEL014181-PA, partial; receptor-type tyrosine- | -1.84 | 0.00 | 1.17 | 1.18 | 0.75 | 3.13 | 4.43 | 2.00 |
| gene-LOC5567544 | proclotting enzyme; AAEL006136- | -1.84 | 0.00 | 4.39 | 2.73 | 2.06 | 12.72 | 10.32 | 9.33 |
| gene-LOC5569606 | uncharacterized protein | -1.83 | 0.01 | 3.69 | 1.89 | 1.29 | 10.00 | 9.25 | 5.12 |
| gene-LOC5578629 | uncharacterized protein | -1.82 | 0.03 | 4.54 | 1.95 | 3.08 | 14.45 | 9.00 | 11.00 |
| gene-LOC5564988 | serine protease persephone | -1.82 | 0.02 | 1.71 | 0.57 | 0.76 | 2.94 | 3.01 | 4.62 |
| gene-LOC5571053 | serine protease SP24D | -1.82 | 0.00 | 15.22 | 17.56 | 9.32 | 55.97 | 45.10 | 48.59 |
| gene-LOC11067617[illegible] | uncharacterized protein | -1.80 | 0.03 | 12.88 | 5.16 | 1.98 | 30.66 | 26.80 | 11.81 |
| gene-LOC5578455 | uncharacterized protein | -1.78 | 0.00 | 3.20 | 5.24 | 6.72 | 19.73 | 19.44 | 13.91 |
| gene-LOC5564745 | 4-hydroxyphenylpyruvate | -1.76 | 0.00 | 6.13 | 3.82 | 2.67 | 18.09 | 9.81 | 15.03 |
| gene-LOC5565434 | leucine-rich repeat-containing | -1.75 | 0.01 | 5.05 | 2.59 | 1.70 | 10.88 | 14.16 | 6.35 |
| gene-LOC5569806 | uncharacterized protein | -1.74 | 0.00 | 3.05 | 2.80 | 2.36 | 9.69 | 7.12 | 10.90 |
| gene-LOC23687431 | mucin-5AC isoform X1-X4 | -1.74 | 0.01 | 0.13 | 0.10 | 0.12 | 0.38 | 0.55 | 0.27 |
| gene-LOC5572513 | uncharacterized protein | -1.73 | 0.03 | 2.83 | 2.54 | 1.97 | 7.34 | 11.96 | 3.39 |

| | | | | | | | | | |
|---|---|---|---|---|---|---|---|---|---|
| gene-LOC11067415( | heat shock protein 70 A1 | -1.73 | 0.00 | 1.19 | 0.67 | 1.42 | 3.01 | 5.48 | 2.53 |
| gene-LOC11067652 | putative RNA-dependent RNA | -1.73 | 0.00 | 2.57 | 1.77 | 1.49 | 3.44 | 9.27 | 6.86 |
| gene-LOC5569553 | transmembrane protein 205 | -1.71 | 0.00 | 11.41 | 10.06 | 11.62 | 36.12 | 31.69 | 41.60 |
| gene-LOC5571641 | laccase-2 | -1.70 | 0.03 | 14.02 | 8.33 | 14.01 | 69.15 | 28.46 | 22.03 |
| gene-LOC11067415 | heat shock protein 70 A1-like | -1.69 | 0.01 | 1.03 | 0.87 | 2.24 | 3.84 | 6.46 | 2.98 |
| gene-LOC5564274 | uncharacterized protein | -1.69 | 0.05 | 0.81 | 1.47 | 0.49 | 3.28 | 3.15 | 2.59 |
| gene-LOC5574170 | tryptase isoform X1/X2 | -1.69 | 0.00 | 8.17 | 4.23 | 4.72 | 24.58 | 20.49 | 10.65 |
| gene-LOC5574116 | solute carrier organic anion | -1.68 | 0.00 | 1.09 | 0.72 | 0.63 | 2.45 | 2.73 | 2.68 |
| gene-LOC5576617 | uncharacterized protein | -1.67 | 0.00 | 42.50 | 15.17 | 19.47 | 112.99 | 77.77 | 54.84 |
| gene-LOC5575349 | tryptase | -1.66 | 0.00 | 7.38 | 5.40 | 3.65 | 27.84 | 17.79 | 8.41 |
| novel2571 | AAEL003593-PA | -1.66 | 0.01 | 3.61 | 2.92 | 2.42 | 14.42 | 10.26 | 4.49 |
| gene-LOC5574916 | solute carrier family 45 member 4 | -1.66 | 0.04 | 0.81 | 0.66 | 0.25 | 2.14 | 1.39 | 1.96 |
| gene-LOC5577040 | serine protease 55 isoform X1; limulus clotting factor C isoform X2 | -1.66 | 0.00 | 3.70 | 4.32 | 2.26 | 11.23 | 10.19 | 11.99 |
| gene-LOC5572116 | Niemann-Pick type protein | -1.64 | 0.00 | 1.20 | 0.83 | 1.05 | 2.50 | 3.94 | 3.15 |
| gene-LOC5570479 | leucine-rich repeat transmembrane protein FLRT3 isoform X2; leucine-rich repeat transmembrane | -1.63 | 0.00 | 18.46 | 7.96 | 6.26 | 34.24 | 39.90 | 26.95 |
| gene-LOC5570596 | serine protease 42 | -1.63 | 0.02 | 1.84 | 0.74 | 1.23 | 4.12 | 5.38 | 2.31 |
| gene-LOC5578664 | AAEL003626-PA; sodium-dependent nutrient amino acid | -1.62 | 0.00 | 20.05 | 15.14 | 12.85 | 36.58 | 62.16 | 49.84 |
| gene-LOC5577601 | larval cuticle protein LCP-30 | -1.62 | 0.00 | 27.70 | 13.62 | 10.99 | 73.36 | 30.09 | 58.80 |
| gene-LOC23687754 | flocculation protein FLO11 | -1.62 | 0.01 | 57.92 | 38.29 | 50.52 | 244.34 | 116.27 | 95.60 |
| novel5205 | - | -1.61 | 0.05 | 1.11 | 1.24 | 1.16 | 4.23 | 4.64 | 1.87 |
| gene-LOC5567194 | surface antigen CRP170 | -1.61 | 0.00 | 17.80 | 11.27 | 9.64 | 55.91 | 36.22 | 26.68 |
| gene-LOC5564764 | cytochrome P450 9e2 | -1.60 | 0.00 | 1.27 | 1.72 | 1.24 | 2.96 | 5.36 | 4.56 |
| gene-LOC11068065 | limulus clotting factor C-like, partial; serine protease persephone-like | -1.60 | 0.02 | 1.43 | 3.09 | 0.58 | 4.68 | 4.61 | 5.17 |
| gene-LOC5569163 | cysteine dioxygenase type 1 | -1.58 | 0.02 | 2.26 | 2.34 | 1.09 | 8.37 | 6.05 | 3.16 |
| gene-LOC5568004 | polyserase-2 isoform X1; serine | -1.58 | 0.00 | 2.06 | 1.87 | 2.30 | 8.83 | 5.67 | 3.97 |
| gene-LOC5575552 | arginase, hepatic | -1.57 | 0.04 | 5.09 | 4.21 | 0.92 | 8.65 | 11.15 | 10.53 |
| gene-LOC5578315 | homogentisate 1,2-dioxygenase | -1.56 | 0.00 | 2.70 | 1.99 | 1.94 | 6.97 | 5.57 | 7.16 |
| gene-LOC11067473 | serine protease snake-like isoform | -1.56 | 0.01 | 6.48 | 6.00 | 3.55 | 21.77 | 15.93 | 8.70 |
| gene-LOC5564054 | UNC93-like protein | -1.55 | 0.00 | 5.61 | 4.28 | 4.66 | 12.67 | 13.86 | 16.27 |
| gene-LOC5568131 | troponin C isoform X1/X2/X3/X4 | -1.55 | 0.04 | 6.00 | 2.21 | 2.68 | 7.29 | 18.95 | 5.10 |

| | | | | | | | | | |
|---|---|---|---|---|---|---|---|---|---|
| gene-LOC11067560[illegible] | uncharacterized protein | -1.54 | 0.00 | 50.04 | 27.03 | 60.43 | 151.49 | 144.04 | 108.17 |
| gene-LOC5563550 | serine protease easter | -1.54 | 0.01 | 2.88 | 1.58 | 1.28 | 7.21 | 5.64 | 3.91 |
| gene-LOC5568638 | sodium-coupled monocarboxylate | -1.54 | 0.00 | 1.83 | 1.85 | 2.18 | 6.99 | 4.41 | 5.76 |
| gene-LOC5565699 | beta-hexosaminidase subunit beta | -1.54 | 0.03 | 4.95 | 4.14 | 3.69 | 27.75 | 8.77 | 5.79 |
| gene-LOC5573598 | venom serine protease Bi-VSP | -1.54 | 0.00 | 11.31 | 8.09 | 7.52 | 32.65 | 25.81 | 20.78 |
| gene-LOC5575399 | venom allergen 5 | -1.53 | 0.00 | 4.53 | 4.17 | 2.34 | 9.49 | 10.76 | 11.75 |
| gene-LOC5575401 | venom allergen 5 | -1.53 | 0.00 | 12.12 | 8.76 | 7.45 | 29.78 | 28.46 | 24.39 |
| gene-LOC5571297 | 2-hydroxyacylsphingosine 1-beta- | -1.52 | 0.04 | 43.67 | 26.74 | 19.48 | 65.21 | 57.96 | 133.97 |
| gene-LOC5572684 | uncharacterized protein | -1.52 | 0.02 | 0.63 | 0.42 | 0.31 | 1.27 | 1.60 | 1.05 |
| gene-LOC5578506 | trypsin 5G1-like | -1.52 | 0.02 | 15.38 | 19.14 | 6.60 | 24.29 | 65.57 | 26.89 |
| gene-LOC5573855 | zinc carboxypeptidase A 1 | -1.52 | 0.00 | 14.88 | 13.16 | 17.91 | 39.28 | 44.11 | 49.27 |
| gene-LOC5579505 | protein NPC2 homolog | -1.51 | 0.00 | 8.95 | 9.38 | 8.57 | 34.04 | 24.44 | 19.42 |
| gene-LOC5563676 | cholinesterase 2 | -1.51 | 0.02 | 1.17 | 0.79 | 1.33 | 4.00 | 3.26 | 2.21 |
| gene-LOC5570819 | CD109 antigen isoform X1-X12 | -1.51 | 0.00 | 22.23 | 15.07 | 13.69 | 49.25 | 54.64 | 32.05 |
| gene-LOC5578210 | uncharacterized protein | -1.51 | 0.00 | 17.92 | 12.71 | 5.77 | 41.27 | 27.25 | 35.35 |
| gene-LOC5567033 | glucose dehydrogenase | -1.50 | 0.03 | 4.24 | 2.59 | 1.21 | 12.98 | 5.14 | 5.15 |
| gene-LOC23687423 | serine protease easter isoform | -1.50 | 0.00 | 6.63 | 3.24 | 4.00 | 10.41 | 15.60 | 11.77 |
| gene-LOC5565301 | protein BTG1 | -1.50 | 0.00 | 0.87 | 0.49 | 0.51 | 1.58 | 1.68 | 2.05 |
| gene-LOC5577270 | guanine nucleotide-binding protein | -1.49 | 0.00 | 4.94 | 4.64 | 5.31 | 15.79 | 15.83 | 10.88 |
| gene-LOC5568175 | uncharacterized protein | -1.49 | 0.00 | 2.08 | 3.33 | 2.29 | 6.24 | 7.58 | 7.99 |
| gene-LOC11067724[illegible] | uncharacterized protein | -1.49 | 0.01 | 2.37 | 1.20 | 1.81 | 4.74 | 5.72 | 4.69 |
| novel5467 | protein midgut expression 1 | -1.48 | 0.00 | 2.54 | 2.90 | 3.62 | 13.68 | 9.29 | 5.14 |
| gene-LOC5575400 | venom allergen 5 | -1.48 | 0.00 | 5.93 | 4.55 | 3.23 | 14.21 | 12.91 | 11.44 |
| gene-LOC5566467 | annexin B10 | -1.46 | 0.00 | 8.71 | 5.47 | 4.63 | 13.64 | 20.66 | 18.03 |
| gene-LOC11067461[illegible] | uncharacterized protein | -1.46 | 0.01 | 0.59 | 1.02 | 1.18 | 1.57 | 2.62 | 2.07 |
| gene-LOC5570911 | acylphosphatase-2 | -1.45 | 0.02 | 9.28 | 6.74 | 7.31 | 19.39 | 35.32 | 9.18 |
| gene-LOC5572848 | putative uncharacterized protein | -1.45 | 0.00 | 6.43 | 3.43 | 3.09 | 11.88 | 14.12 | 9.53 |
| gene-LOC11068080[illegible] | lateral signaling target protein 2 | -1.45 | 0.00 | 7.99 | 4.21 | 6.49 | 23.52 | 15.32 | 12.54 |
| gene-LOC5564245 | uncharacterized protein | -1.45 | 0.01 | 0.28 | 0.19 | 0.24 | 0.65 | 0.84 | 0.46 |
| gene-LOC5575393 | venom allergen 5 | -1.44 | 0.01 | 3.37 | 3.37 | 4.00 | 10.84 | 7.39 | 11.37 |
| gene-LOC5575560 | elongation of very long chain fatty | -1.44 | 0.00 | 24.99 | 1.99 | 4.55 | 4.06 | 4.04 | 4.50 |
| gene-LOC5571127 | uncharacterized protein | -1.43 | 0.00 | 8.29 | 7.31 | 8.28 | 22.85 | 25.59 | 16.40 |
| gene-LOC5565306 | general odorant-binding protein 66 | -1.43 | 0.04 | 3.55 | 3.03 | 3.10 | 11.01 | 5.82 | 9.72 |
| gene-LOC5578738 | glia-derived nexin | -1.43 | 0.00 | 7.54 | 5.48 | 4.21 | 16.71 | 18.59 | 13.16 |
| novel2199 | AAEL005098-PA | -1.42 | 0.00 | 919.79 | 511.01 | 527.22 | 1482.27 | 1873.79 | 1930.22 |

| | | | | | | | | | |
|---|---|---|---|---|---|---|---|---|---|
| gene-LOC5570851 | ATP-binding cassette sub-family G | -1.42 | 0.05 | 0.67 | 0.55 | 0.75 | 1.83 | 1.90 | 1.57 |
| gene-LOC5574469 | esterase B1 | -1.42 | 0.02 | 2.22 | 0.89 | 1.19 | 4.85 | 4.03 | 3.25 |
| gene-LOC5565763 | uncharacterized protein | -1.41 | 0.02 | 1.12 | 0.53 | 0.31 | 1.41 | 2.18 | 1.63 |
| gene-LOC5578131 | flexible cuticle protein 12 | -1.41 | 0.03 | 67.41 | 25.54 | 21.96 | 145.08 | 46.88 | 111.98 |
| gene-LOC5571916 | putative helicase MOV-10 | -1.40 | 0.00 | 7.05 | 5.79 | 5.20 | 17.26 | 14.72 | 18.90 |
| gene-LOC5579662 | elongation of very long chain fatty | -1.40 | 0.00 | 2.36 | 2.03 | 1.79 | 6.45 | 4.21 | 5.78 |
| gene-LOC5570482 | leucine-rich repeat transmembrane | -1.40 | 0.01 | 13.91 | 6.79 | 5.02 | 26.78 | 24.71 | 16.38 |
| gene-LOC5569608 | uncharacterized protein | -1.39 | 0.05 | 12.42 | 5.40 | 4.72 | 30.87 | 17.86 | 12.10 |
| gene-LOC11067920 | uncharacterized protein | -1.39 | 0.02 | 9.23 | 6.99 | 6.16 | 27.33 | 15.35 | 11.40 |
| gene-LOC5575342 | general odorant-binding protein | -1.39 | 0.00 | 29.23 | 17.09 | 17.98 | 78.64 | 43.93 | 45.94 |
| gene-LOC11067807 | glycine-rich protein DOT1-like; glycine-rich protein 23-like | -1.39 | 0.03 | 13.89 | 6.88 | 6.86 | 25.83 | 29.63 | 16.81 |
| gene-LOC5570847 | 37 kDa salivary gland allergen Aed | -1.38 | 0.03 | 6.51 | 2.93 | 1.80 | 9.84 | 11.02 | 8.53 |
| gene-LOC5564497 | uncharacterized protein | -1.38 | 0.00 | 12.25 | 14.06 | 12.75 | 33.45 | 35.42 | 33.98 |
| gene-LOC5578105 | regulator of microtubule dynamics | -1.37 | 0.01 | 15.70 | 6.09 | 13.97 | 47.39 | 25.64 | 20.67 |
| gene-LOC5573843 | uncharacterized protein | -1.37 | 0.00 | 3.72 | 3.70 | 2.28 | 7.05 | 8.25 | 10.02 |
| gene-LOC5567883 | sulfotransferase family cytosolic 1B | -1.37 | 0.00 | 5.63 | 4.45 | 5.25 | 16.80 | 8.21 | 15.21 |
| gene-LOC11068145 | putative nuclease HARBI1 | -1.36 | 0.00 | 3.24 | 2.00 | 2.62 | 7.53 | 6.23 | 6.52 |
| gene-LOC11067958 | myb-like protein V | -1.35 | 0.00 | 11.36 | 8.13 | 8.00 | 23.39 | 29.39 | 17.68 |
| gene-LOC5579042 | uncharacterized protein | -1.35 | 0.03 | 11.60 | 6.87 | 5.40 | 24.89 | 27.29 | 8.94 |
| novel8131 | hypothetical protein | -1.34 | 0.01 | 0.37 | 0.43 | 0.38 | 0.96 | 1.16 | 0.88 |
| gene-LOC5576417 | uncharacterized protein | -1.34 | 0.00 | 1.78 | 1.29 | 1.37 | 3.83 | 5.11 | 2.33 |
| gene-LOC5568779 | facilitated trehalose transporter Tret1-2 homolog isoform X1/X2 | -1.33 | 0.00 | 8.45 | 4.17 | 6.44 | 16.38 | 11.42 | 20.53 |
| gene-LOC5578124 | uncharacterized protein | -1.32 | 0.01 | 1.25 | 0.78 | 0.87 | 2.64 | 1.95 | 2.73 |
| gene-LOC5563689 | pacifastin-like protease inhibitor | -1.32 | 0.00 | 22.01 | 12.64 | 11.93 | 61.65 | 31.17 | 24.40 |
| gene-LOC5573277 | multidrug resistance protein | -1.32 | 0.00 | 4.48 | 3.68 | 3.83 | 13.32 | 11.73 | 5.18 |
| gene-LOC5573074 | venom allergen 5 isoform X1/X2 | -1.32 | 0.00 | 5.37 | 4.05 | 2.57 | 10.52 | 9.63 | 9.89 |
| gene-LOC5573693 | AAEL010686-PA; ras-related and estrogen-regulated growth inhibitor | -1.31 | 0.00 | 1.12 | 1.57 | 1.08 | 3.57 | 4.12 | 2.01 |
| gene-LOC5567253 | ER lumen protein-retaining | -1.31 | 0.00 | 14.88 | 11.78 | 9.10 | 33.53 | 35.38 | 20.21 |
| gene-LOC11067561 | uncharacterized protein | -1.31 | 0.00 | 106.86 | 78.31 | 98.04 | 271.39 | 263.93 | 173.15 |
| gene-LOC5567682 | serine protease easter isoform X2; transmembrane protease serine 9 | -1.31 | 0.03 | 2.39 | 0.99 | 1.85 | 5.70 | 3.77 | 3.32 |
| gene-LOC5570229 | fatty acid synthase | -1.30 | 0.01 | 4.27 | 1.97 | 1.23 | 6.58 | 5.60 | 6.26 |
| gene-LOC5572622 | uncharacterized protein | -1.30 | 0.00 | 9.13 | 5.71 | 4.25 | 15.60 | 12.34 | 19.24 |

| | | | | | | | | |
|---|---|---|---|---|---|---|---|---|
| gene-LOC5573776 | gamma-glutamyl hydrolase isoform | -1.29 | 0.00 | 16.18 | 15.40 | 12.22 | 42.87 | 37.45 | 29.65 |
| gene-LOC5565631 | synaptic vesicle glycoprotein 2B | -1.29 | 0.03 | 2.70 | 1.01 | 2.64 | 5.33 | 4.90 | 5.40 |
| gene-LOC5578154 | AAEL013532-PB; uncharacterized protein LOC5578154 isoform | -1.29 | 0.03 | 8.37 | 1.80 | 5.88 | 13.19 | 13.74 | 12.31 |
| gene-LOC5574270 | integrin alpha-PS3 | -1.29 | 0.00 | 9.08 | 7.47 | 7.20 | 17.04 | 23.90 | 18.02 |
| gene-LOC5568364 | uncharacterized protein | -1.29 | 0.01 | 9.42 | 6.91 | 7.41 | 17.07 | 29.60 | 11.42 |
| gene-LOC5571667 | uncharacterized protein | -1.29 | 0.01 | 13.57 | 7.60 | 5.39 | 26.32 | 22.58 | 15.48 |
| gene-LOC5570331 | uncharacterized protein | -1.29 | 0.00 | 47.80 | 35.25 | 34.56 | 124.02 | 70.07 | 96.82 |
| gene-LOC5578122 | uncharacterized protein | -1.28 | 0.00 | 3.37 | 1.63 | 2.17 | 6.73 | 4.93 | 5.89 |
| gene-LOC5570039 | uncharacterized protein | -1.28 | 0.03 | 13.65 | 12.21 | 6.39 | 29.55 | 29.75 | 17.88 |
| gene-LOC5568782 | facilitated trehalose transporter | -1.28 | 0.01 | 7.84 | 3.33 | 6.47 | 16.03 | 16.28 | 10.68 |
| gene-LOC5575868 | cysteine-rich secretory protein | -1.27 | 0.00 | 2.62 | 1.69 | 1.34 | 5.40 | 4.20 | 4.17 |
| gene-LOC5564892 | organic cation transporter protein | -1.27 | 0.04 | 8.35 | 9.68 | 4.38 | 9.18 | 6.61 | 21.12 |
| novel5266 | pol-like protein | -1.26 | 0.04 | 0.28 | 0.65 | 0.48 | 1.00 | 0.90 | 1.52 |
| gene-LOC5567410 | shootin-1 | -1.26 | 0.00 | 5.14 | 4.58 | 3.69 | 8.99 | 9.72 | 13.80 |
| gene-LOC5575634 | protein takeout | -1.26 | 0.00 | 5.03 | 4.30 | 4.22 | 11.67 | 8.41 | 12.67 |
| gene-LOC110681162 | protein takeout | -1.26 | 0.00 | 5.03 | 4.30 | 4.22 | 11.67 | 8.41 | 12.67 |
| gene-LOC110676188 | uncharacterized protein | -1.26 | 0.00 | 62.10 | 38.37 | 34.75 | 120.30 | 117.51 | 83.06 |
| gene-LOC5576180 | general odorant-binding protein 72 | -1.25 | 0.00 | 12.91 | 9.86 | 15.32 | 31.68 | 31.38 | 30.16 |
| gene-LOC5576721 | COX assembly mitochondrial | -1.25 | 0.02 | 10.11 | 6.94 | 4.99 | 32.24 | 19.15 | 11.30 |
| gene-LOC5570483 | acidic leucine-rich nuclear phosphoprotein 32 family member | -1.24 | 0.03 | 19.64 | 8.23 | 6.08 | 25.71 | 34.04 | 20.62 |
| gene-LOC110678504 | uncharacterized protein | -1.23 | 0.03 | 38.98 | 40.71 | 20.82 | 106.41 | 96.53 | 33.39 |
| novel275 | - | -1.23 | 0.00 | 0.90 | 1.02 | 1.09 | 2.77 | 1.70 | 2.77 |
| gene-LOC110679707 | serine protease 7-like isoform | -1.23 | 0.02 | 9.49 | 5.87 | 4.51 | 23.84 | 13.65 | 10.49 |
| gene-LOC5570134 | AAEL008099-PA; procollagen-lysine,2-oxoglutarate 5- | -1.23 | 0.00 | 5.98 | 4.95 | 3.68 | 14.61 | 10.27 | 9.12 |
| gene-LOC5575338 | leucine-rich repeat neuronal protein | -1.22 | 0.02 | 8.27 | 5.02 | 4.88 | 18.00 | 17.82 | 6.83 |
| gene-LOC5571253 | tolloid-like protein 1 | -1.21 | 0.03 | 1.41 | 0.76 | 0.77 | 2.89 | 1.92 | 2.03 |
| gene-LOC110674832 | uncharacterized protein | -1.21 | 0.00 | 37.48 | 25.39 | 15.95 | 64.33 | 51.99 | 64.30 |
| gene-LOC5575074 | WAP, Kazal, immunoglobulin, Kunitz and NTR domain-containing | -1.21 | 0.00 | 18.93 | 14.78 | 12.51 | 39.48 | 28.75 | 39.56 |
| gene-LOC5568783 | facilitated trehalose transporter | -1.21 | 0.04 | 4.01 | 1.56 | 2.39 | 6.14 | 6.85 | 5.39 |
| gene-LOC5579888 | mucin-6 isoform X1/X2 | -1.20 | 0.00 | 5.47 | 2.63 | 2.82 | 7.84 | 6.12 | 11.37 |
| gene-LOC5577037 | ovochymase-2 isoform X1; | -1.20 | 0.02 | 1.01 | 0.83 | 0.94 | 2.33 | 2.17 | 1.81 |

| | | | | | | | | | |
|---|---|---|---|---|---|---|---|---|---|
| gene-LOC5571531 | probable cytochrome P450 6a20 | -1.20 | 0.01 | 1.87 | 2.53 | 2.21 | 4.51 | 6.29 | 4.52 |
| gene-LOC5564510 | cytochrome b5-related protein | -1.20 | 0.00 | 26.56 | 16.51 | 10.57 | 46.43 | 48.15 | 29.23 |
| gene-LOC5571025 | dynein assembly factor 1, | -1.20 | 0.00 | 5.54 | 4.57 | 4.19 | 10.32 | 11.42 | 11.31 |
| gene-LOC5571279 | peptidyl-alpha-hydroxyglycine | -1.19 | 0.00 | 2.33 | 2.34 | 2.48 | 4.71 | 6.55 | 5.23 |
| gene-LOC5567003 | serine protease snake isoform | -1.19 | 0.01 | 4.64 | 3.40 | 2.78 | 9.41 | 9.08 | 6.44 |
| gene-LOC5573853 | serine protease inhibitor 28Dc | -1.19 | 0.00 | 21.05 | 18.43 | 14.19 | 37.34 | 55.28 | 29.88 |
| novel2418 | - | -1.19 | 0.00 | 0.67 | 0.61 | 0.79 | 1.90 | 1.61 | 1.27 |
| novel384 | - | -1.19 | 0.01 | 1.87 | 1.06 | 1.29 | 3.40 | 3.34 | 2.50 |
| gene-LOC5576084 | 2-amino-3-ketobutyrate coenzyme | -1.18 | 0.04 | 4.68 | 2.34 | 2.52 | 8.32 | 4.95 | 8.60 |
| novel187 | - | -1.18 | 0.01 | 0.63 | 0.93 | 0.44 | 1.36 | 1.69 | 1.52 |
| gene-LOC5570040 | Vago protein; uncharacterized | -1.17 | 0.03 | 30.20 | 26.57 | 10.24 | 49.33 | 43.94 | 53.19 |
| gene-LOC5577357 | monocarboxylate transporter 5 | -1.17 | 0.02 | 2.10 | 1.26 | 1.47 | 4.58 | 4.18 | 2.15 |
| gene-LOC5567584 | general odorant-binding protein | -1.16 | 0.01 | 10.04 | 5.87 | 9.72 | 16.52 | 20.23 | 21.40 |
| gene-LOC5576245 | tubulin beta-3 chain | -1.15 | 0.03 | 4.04 | 2.89 | 4.83 | 13.75 | 7.46 | 5.24 |
| gene-LOC5578266 | transcription factor AP-1 isoform | -1.15 | 0.00 | 23.48 | 16.70 | 24.50 | 48.31 | 54.07 | 38.13 |
| gene-LOC5573760 | mini-chromosome maintenance complex-binding protein; lysozyme | -1.15 | 0.05 | 12.38 | 7.29 | 8.08 | 31.37 | 17.30 | 13.52 |
| gene-LOC5574523 | cholesterol 7-desaturase | -1.15 | 0.02 | 4.97 | 3.99 | 4.85 | 12.69 | 12.65 | 5.45 |
| gene-LOC5564164 | hemicentin-1 | -1.14 | 0.01 | 3.11 | 1.89 | 1.35 | 3.98 | 4.77 | 5.32 |
| gene-LOC5569577 | uncharacterized protein | -1.14 | 0.04 | 7.97 | 6.48 | 5.72 | 21.08 | 12.64 | 9.93 |
| gene-LOC5578682 | acyl-CoA Delta(11) desaturase | -1.13 | 0.01 | 5.02 | 2.83 | 2.84 | 7.17 | 8.11 | 8.35 |
| gene-LOC5578728 | uncharacterized protein | -1.13 | 0.00 | 8.50 | 6.22 | 5.07 | 15.21 | 16.97 | 11.63 |
| gene-LOC5567431 | uncharacterized protein | -1.13 | 0.00 | 12.31 | 9.58 | 11.27 | 27.76 | 26.63 | 18.97 |
| gene-LOC5572583 | AAEL009910-PA; uncharacterized protein LOC5572583 isoform X5/X7; PDZ and LIM domain | -1.13 | 0.03 | 5.07 | 2.47 | 4.88 | 8.68 | 14.22 | 6.51 |
| gene-LOC5572453 | sodium-coupled monocarboxylate transporter 2-like; sodium-coupled | -1.12 | 0.05 | 3.22 | 1.75 | 2.25 | 5.72 | 5.90 | 3.83 |
| gene-LOC5578692 | serine protease easter | -1.12 | 0.00 | 10.21 | 9.96 | 7.68 | 25.87 | 17.08 | 18.07 |
| gene-LOC5574136 | alpha-tocopherol transfer protein- | -1.12 | 0.00 | 3.67 | 3.87 | 3.36 | 7.73 | 8.39 | 7.02 |
| gene-LOC5568744 | pyridoxal phosphate phosphatase | -1.12 | 0.03 | 2.70 | 3.08 | 5.05 | 8.22 | 6.70 | 8.76 |
| gene-LOC5565708 | protein spaetzle 3; AAEL014950- | -1.12 | 0.00 | 8.03 | 4.59 | 5.86 | 15.05 | 16.07 | 9.74 |
| gene-LOC5568362 | melanization protease 1 | -1.12 | 0.02 | 8.30 | 8.00 | 4.94 | 21.47 | 12.80 | 12.16 |
| gene-LOC5565208 | uncharacterized protein | -1.12 | 0.00 | 16.51 | 13.41 | 11.49 | 26.44 | 30.84 | 34.49 |
| gene-LOC5567063 | uncharacterized protein | -1.11 | 0.03 | 1.99 | 1.77 | 1.57 | 5.59 | 3.73 | 2.32 |

| | | | | | | | | | |
|---|---|---|---|---|---|---|---|---|---|
| novel8219 | - | -1.11 | 0.00 | 10.38 | 13.95 | 14.60 | 30.86 | 24.70 | 29.45 |
| novel189 | - | -1.10 | 0.00 | 1.14 | 1.35 | 0.79 | 3.05 | 1.95 | 2.13 |
| gene-LOC5566992 | protein yellow | -1.10 | 0.05 | 2.30 | 1.47 | 1.16 | 4.27 | 3.92 | 2.47 |
| gene-LOC5564753 | probable cytochrome P450 9f2 | -1.10 | 0.02 | 10.69 | 8.05 | 8.09 | 33.62 | 10.49 | 18.11 |
| gene-LOC5566826 | adenosine deaminase 2 | -1.09 | 0.04 | 22.20 | 7.88 | 8.53 | 32.41 | 22.88 | 26.19 |
| gene-LOC5571540 | probable cytochrome P450 6a14 | -1.09 | 0.00 | 5.69 | 4.84 | 4.71 | 13.89 | 7.65 | 11.25 |
| gene-LOC5575179 | AAEL000304-PB; RNA-binding | -1.09 | 0.00 | 26.22 | 17.80 | 18.50 | 33.49 | 35.11 | 51.37 |
| gene_gene-CFI06_m | NADH dehydrogenase subunit 5 | -1.09 | 0.00 | 33.76 | 32.78 | 41.67 | 104.46 | 84.77 | 42.53 |
| gene-LOC5575341 | general odorant-binding protein | -1.08 | 0.03 | 8.04 | 10.05 | 13.76 | 31.82 | 19.11 | 18.02 |
| novel5021 | putative salivary secreted peptide | -1.08 | 0.00 | 32.13 | 23.46 | 17.86 | 51.90 | 64.97 | 38.72 |
| gene-LOC5563618 | uncharacterized protein | -1.08 | 0.02 | 5.67 | 4.19 | 5.89 | 13.97 | 10.99 | 8.39 |
| gene-LOC5566203 | ABC transporter G family member | -1.07 | 0.01 | 12.21 | 7.29 | 10.70 | 30.79 | 23.13 | 15.03 |
| gene-LOC5566878 | uncharacterized protein | -1.07 | 0.00 | 4.55 | 4.01 | 3.45 | 9.39 | 8.76 | 6.54 |
| gene-LOC5574166 | ras-associated and pleckstrin homology domains-containing | -1.06 | 0.02 | 3.72 | 2.21 | 2.81 | 6.75 | 7.98 | 3.68 |
| gene-LOC5571433 | LOW QUALITY PROTEIN: | -1.06 | 0.00 | 3.52 | 2.58 | 2.90 | 6.63 | 7.39 | 4.89 |
| gene-LOC5569758 | uncharacterized protein LOC5569758; spermatogenesis- | -1.06 | 0.04 | 1.32 | 0.89 | 0.88 | 3.57 | 2.84 | 2.17 |
| gene-LOC5567327 | disintegrin and metalloproteinase domain-containing protein 12 | -1.06 | 0.00 | 8.40 | 3.97 | 5.22 | 11.72 | 12.82 | 12.28 |
| gene-LOC5573927 | fatty acid synthase | -1.06 | 0.02 | 10.34 | 4.49 | 3.73 | 12.63 | 13.10 | 13.19 |
| gene-LOC5569978 | sushi, von Willebrand factor type A, EGF and pentraxin domain- | -1.06 | 0.05 | 0.85 | 0.46 | 0.45 | 1.32 | 1.47 | 0.90 |
| novel2139 | - | -1.05 | 0.04 | 2.36 | 2.77 | 2.99 | 6.53 | 7.30 | 3.07 |
| gene-LOC5569000 | acyl-CoA synthetase short-chain family member 3, mitochondrial | -1.05 | 0.03 | 4.20 | 2.41 | 3.13 | 8.03 | 7.91 | 4.82 |
| gene-LOC5565973 | myrosinase 1 | -1.05 | 0.03 | 3.89 | 2.54 | 2.02 | 6.85 | 4.70 | 6.04 |
| gene-LOC5564692 | uncharacterized protein | -1.04 | 0.02 | 2.70 | 3.50 | 2.96 | 8.33 | 5.13 | 5.65 |
| gene-LOC5573368 | uncharacterized protein | -1.04 | 0.01 | 5.34 | 5.32 | 4.94 | 12.83 | 12.48 | 7.09 |
| gene-LOC5567097 | uridine phosphorylase 1 isoform | -1.04 | 0.00 | 11.13 | 9.19 | 8.12 | 25.15 | 19.01 | 14.84 |
| novel300 | - | -1.04 | 0.04 | 1.18 | 0.75 | 0.78 | 1.64 | 1.86 | 2.09 |
| gene-LOC5568649 | UDP-glucose 4-epimerase | -1.04 | 0.03 | 2.35 | 2.66 | 2.35 | 5.51 | 4.40 | 5.21 |
| gene-LOC5566877 | probable G-protein coupled | -1.04 | 0.00 | 7.25 | 7.85 | 7.18 | 15.55 | 12.70 | 17.82 |
| gene-LOC5574133 | cysteine sulfinic acid | -1.04 | 0.00 | 7.46 | 7.20 | 5.42 | 14.09 | 13.79 | 13.55 |
| gene-LOC5571779 | lachesin | -1.03 | 0.00 | 25.64 | 15.72 | 11.80 | 33.45 | 37.33 | 38.48 |

| | | | | | | | | | |
|---|---|---|---|---|---|---|---|---|---|
| gene-LOC5577716 | protein lethal(2)essential for life | -1.03 | 0.00 | 21.51 | 16.44 | 15.31 | 38.20 | 43.49 | 27.61 |
| gene-LOC5566102 | membrane-bound alkaline | -1.03 | 0.01 | 4.02 | 4.13 | 3.22 | 8.93 | 6.09 | 8.46 |
| gene-LOC5576475 | uncharacterized protein | -1.03 | 0.01 | 32.76 | 17.61 | 15.36 | 47.63 | 37.63 | 49.76 |
| gene-LOC5576981 | venom allergen 5; AAEL003053-PF | -1.03 | 0.03 | 76.16 | 33.00 | 26.09 | 98.05 | 81.99 | 96.12 |
| gene-LOC23687933 | glycine-rich cell wall structural | -1.03 | 0.02 | 14.17 | 6.51 | 10.05 | 27.89 | 18.31 | 18.26 |
| gene-LOC5571722 | coronin-7 isoform X1-X4 | -1.02 | 0.02 | 3.19 | 1.39 | 1.85 | 4.50 | 4.53 | 4.07 |
| gene-LOC5574171 | AAEL002288-PA; trypsin-3 | -1.02 | 0.04 | 3.94 | 3.12 | 2.56 | 8.61 | 6.19 | 4.72 |
| gene-LOC11067701 | uncharacterized protein | -1.02 | 0.01 | 104.61 | 45.65 | 53.03 | 177.20 | 122.33 | 114.74 |
| gene-LOC5564068 | uncharacterized protein | -1.02 | 0.02 | 1.25 | 1.67 | 1.53 | 3.03 | 2.21 | 3.81 |
| gene-LOC5574499 | Krueppel homolog 1 isoform X1/X2 | -1.02 | 0.03 | 4.17 | 2.20 | 2.66 | 8.92 | 5.10 | 4.58 |
| novel2568 | AAEL000363-PA | -1.02 | 0.00 | 353.80 | 334.86 | 354.02 | 400.26 | 862.28 | 863.05 |
| gene-LOC23687745 | serine protease easter isoform | -1.02 | 0.02 | 11.15 | 10.09 | 5.58 | 18.78 | 21.86 | 13.93 |
| gene-LOC5576615 | uncharacterized protein | -1.01 | 0.03 | 8.72 | 4.33 | 6.48 | 17.19 | 12.61 | 9.94 |
| gene-LOC5564989 | uncharacterized protein | -1.01 | 0.00 | 37.59 | 25.37 | 30.09 | 53.47 | 64.80 | 59.20 |
| gene-LOC5570845 | kallikrein 1-related peptidase b3 | -1.01 | 0.05 | 58.28 | 20.61 | 22.62 | 71.76 | 54.19 | 79.11 |
| novel4956 | glycoprotein | -1.01 | 0.00 | 2.29 | 1.64 | 1.97 | 4.52 | 3.72 | 3.69 |
| gene-LOC5575324 | AAEL002600-PA; serine protease | -1.00 | 0.00 | 30.99 | 21.59 | 20.05 | 52.86 | 40.27 | 35.14 |
| gene-LOC5565836 | uncharacterized protein | -1.00 | 0.00 | 12.93 | 10.23 | 12.82 | 36.32 | 31.86 | 14.77 |
| gene-LOC5574548 | scavenger receptor class B | -1.00 | 0.01 | 9.51 | 6.70 | 8.30 | 16.01 | 13.04 | 11.27 |

**Table S2. Differentially expressed genes in the mosquitoes following feeding on TIBOV-blood comparing to those feeding on the mock-blood at 7 dpi. The downregulated genes were highlighted in blue, whereas the upregulated genes were highlighted orange. Aae-BV-7d-1, 2 and 3 mean TIBOV-infected *Ae. aegypti* at 7 dpi, and Aae-BC-7d-1, 2 and 3 mean Mock-infected *Ae. aegypti* at 7 dpi.**

| Gene_name | NR annotation | $Log_2$(FoldChange) | *P-adj* | FPKM of gene | | | | | |
|---|---|---|---|---|---|---|---|---|---|
| | | | | Aae-BV-7d-1 | Aae-BV-7d-2 | Aae-BV-7d-3 | Aae-BC-7d-1 | Aae-BC-7d-2 | Aae-BC-7d-3 |
| gene-LOC5567680 | opsin-1 | 8.34 | 0.00 | 2797.09 | 3833.92 | 1886.68 | 6.64 | 10.16 | 9.03 |
| gene-LOC5567790 | enolase-phosphatase E1-like | 7.97 | 0.00 | 10.27 | 25.45 | 10.60 | 0.06 | 0.00 | 0.11 |
| gene-LOC5575817 | uncharacterized protein LOC5575817 | 7.74 | 0.00 | 5.86 | 5.30 | 3.12 | 0.00 | 0.07 | 0.00 |
| gene-LOC5577143 | arrestin homolog | 7.06 | 0.00 | 118.84 | 146.51 | 66.26 | 0.55 | 1.26 | 0.65 |
| novel2639 | - | 7.04 | 0.00 | 3.22 | 4.02 | 3.54 | 0.00 | 0.00 | 0.00 |
| gene-LOC5568060 | opsin-1 | 6.95 | 0.00 | 158.14 | 196.93 | 95.90 | 1.04 | 1.21 | 1.31 |
| gene-LOC11068088[illegible] | opsin-1 | 6.95 | 0.00 | 158.14 | 196.93 | 95.90 | 1.04 | 1.21 | 1.31 |
| gene-LOC5568137 | serine protease easter | 6.62 | 0.00 | 1.30 | 1.78 | 0.65 | 0.00 | 0.00 | 0.00 |
| gene-LOC5571503 | mpv17-like protein | 6.39 | 0.00 | 0.33 | 1.99 | 2.55 | 0.00 | 0.00 | 0.00 |
| gene-LOC5576907 | uncharacterized protein LOC5576907 | 6.29 | 0.00 | 1.52 | 3.31 | 1.70 | 0.00 | 0.07 | 0.00 |
| gene-LOC5565425 | uncharacterized protein LOC5565425 | 6.15 | 0.00 | 2.64 | 2.47 | 0.97 | 0.00 | 0.00 | 0.10 |
| gene-LOC5570856 | stress-activated protein kinase JNK | 6.10 | 0.00 | 1.60 | 5.30 | 1.61 | 0.00 | 0.00 | 0.12 |
| gene-LOC11068021[illegible] | uncharacterized protein LOC5566275 | 5.88 | 0.00 | 3.30 | 5.03 | 1.56 | 0.10 | 0.00 | 0.06 |
| gene-LOC5576909 | uncharacterized protein LOC5576909 | 5.67 | 0.00 | 2.94 | 6.54 | 2.94 | 0.23 | 0.00 | 0.00 |
| gene-LOC5568416 | cytochrome P450 4g15 | 5.57 | 0.00 | 11.06 | 34.76 | 12.84 | 0.11 | 0.64 | 0.46 |
| gene-LOC5580038 | 30 kDa salivary gland allergen Aed a 3-like | 5.55 | 0.00 | 35.16 | 71.32 | 20.85 | 0.52 | 1.19 | 0.97 |
| gene-LOC5571242 | hornerin | 5.49 | 0.00 | 3.19 | 6.67 | 1.49 | 0.00 | 0.17 | 0.08 |
| gene-LOC5577495 | uncharacterized protein LOC5577495 | 5.46 | 0.00 | 6.95 | 11.38 | 4.14 | 0.25 | 0.21 | 0.04 |
| gene-LOC5576882 | opsin-3 | 5.38 | 0.00 | 13.47 | 16.07 | 10.03 | 0.26 | 0.50 | 0.18 |
| gene-LOC5574806 | probable chitinase 10 | 5.32 | 0.00 | 22.82 | 55.89 | 47.24 | 0.99 | 1.04 | 0.92 |
| gene-LOC5573896 | uricase | 5.24 | 0.00 | 2.88 | 8.47 | 7.07 | 0.14 | 0.10 | 0.24 |
| gene-LOC5578159 | arrestin homolog | 5.22 | 0.00 | 88.18 | 130.31 | 53.10 | 3.03 | 2.15 | 1.86 |

| | | | | | | | | | |
|---|---|---|---|---|---|---|---|---|---|
| gene-LOC5577775 | uncharacterized protein LOC5577775 | 5.12 | 0.00 | 2.43 | 6.34 | 2.22 | 0.30 | 0.00 | 0.00 |
| gene-LOC5578161 | elongation of very long chain fatty acids protein AAEL008004-like | 5.06 | 0.00 | 0.38 | 4.30 | 1.29 | 0.00 | 0.18 | 0.00 |
| gene-LOC11067400 | protein G12 | 5.03 | 0.00 | 1.07 | 2.89 | 6.90 | 0.00 | 0.05 | 0.33 |
| gene-LOC5578244 | protein G12 | 5.03 | 0.00 | 1.07 | 2.89 | 6.90 | 0.00 | 0.05 | 0.33 |
| gene-LOC5576981 | venom allergen 5; AAEL003053-PF | 5.00 | 0.00 | 8.25 | 21.45 | 4.85 | 0.00 | 0.93 | 0.15 |
| gene-LOC11067569 | uncharacterized protein LOC110675699 | 4.99 | 0.00 | 4.98 | 9.75 | 4.74 | 0.00 | 0.40 | 0.20 |
| gene-LOC23687682 | protein snwA | 4.98 | 0.00 | 9.30 | 9.92 | 5.23 | 0.07 | 0.31 | 0.38 |
| gene-LOC5567953 | uncharacterized protein LOC5567953 | 4.91 | 0.00 | 25.79 | 44.08 | 26.11 | 1.66 | 0.87 | 0.52 |
| gene-LOC11067477 | struthiocalcin-2-like | 4.90 | 0.00 | 6.59 | 13.04 | 8.14 | 0.22 | 0.35 | 0.34 |
| gene-LOC5567956 | 37 kDa salivary gland allergen Aed a 2-like | 4.89 | 0.00 | 25.32 | 50.56 | 14.47 | 1.10 | 1.10 | 0.79 |
| gene-LOC5580040 | 30 kDa salivary gland allergen Aed a 3 | 4.81 | 0.00 | 33.50 | 67.29 | 22.78 | 1.52 | 1.87 | 0.92 |
| gene-LOC5574754 | AAEL011400-PA; uncharacterized protein LOC5574754 | 4.75 | 0.00 | 0.81 | 3.74 | 1.84 | 0.00 | 0.04 | 0.00 |
| gene-LOC5566287 | fibrinogen-like protein A | 4.71 | 0.00 | 8.03 | 11.60 | 5.92 | 0.29 | 0.22 | 0.44 |
| gene-LOC5577335 | MORN repeat-containing protein 4 | 4.66 | 0.00 | 2.39 | 3.16 | 1.62 | 0.14 | 0.14 | 0.00 |
| gene-LOC5580165 | pickpocket protein 28 isoform X2; sodium channel protein Nach isoform X1 | 4.60 | 0.00 | 0.07 | 0.69 | 1.04 | 0.00 | 0.04 | 0.01 |
| novel2269 | hypothetical protein RP20_CCG011225 | 4.55 | 0.00 | 4.30 | 0.19 | 1.84 | 0.14 | 0.00 | 0.12 |
| gene-LOC5575765 | antichymotrypsin-2 | 4.47 | 0.00 | 17.87 | 22.05 | 7.15 | 0.57 | 0.68 | 0.84 |
| gene-LOC5575351 | tryptase | 4.42 | 0.00 | 7.71 | 5.97 | 3.10 | 0.38 | 0.29 | 0.10 |
| gene-LOC23687564 | glycine-rich protein 5 | 4.39 | 0.00 | 9.20 | 13.15 | 7.45 | 0.29 | 0.74 | 0.37 |
| gene-LOC11067421 | venom protease-like | 4.34 | 0.00 | 0.64 | 3.02 | 2.31 | 0.07 | 0.00 | 0.22 |
| gene-LOC5580039 | uncharacterized protein LOC5580039 | 4.32 | 0.00 | 52.10 | 94.00 | 37.31 | 3.98 | 2.31 | 2.33 |
| gene-LOC5577556 | uncharacterized protein LOC5577556 isoform X1 | 4.32 | 0.00 | 1.69 | 2.13 | 0.93 | 0.09 | 0.00 | 0.14 |

| | | | | | | | | | |
|---|---|---|---|---|---|---|---|---|---|
| gene-LOC5570133 | hypothetical protein RP20_CCG021742; guanine nucleotide-binding protein subunit beta-2 | 4.32 | 0.00 | 4.29 | 7.57 | 3.60 | 0.41 | 0.82 | 0.15 |
| gene-LOC5578868 | uncharacterized protein LOC5578868 | 4.30 | 0.01 | 0.24 | 0.32 | 0.46 | 0.05 | 0.00 | 0.00 |
| gene-LOC11068142 | 37 kDa salivary gland allergen Aed a 2 | 4.23 | 0.00 | 38.55 | 62.51 | 19.24 | 1.98 | 2.83 | 1.50 |
| gene-LOC5567871 | uncharacterized protein LOC5567871 isoform X1 | 4.20 | 0.00 | 4.16 | 4.87 | 2.61 | 0.08 | 0.49 | 0.08 |
| gene-LOC5570845 | kallikrein 1-related peptidase b3 | 4.19 | 0.00 | 15.45 | 25.83 | 8.68 | 0.64 | 1.07 | 0.99 |
| gene-LOC5566624 | venom allergen 3 | 4.14 | 0.00 | 42.31 | 53.98 | 29.62 | 2.41 | 2.43 | 2.16 |
| gene-LOC5567877 | apyrase | 4.13 | 0.00 | 15.76 | 24.18 | 10.32 | 0.85 | 0.92 | 1.06 |
| gene-LOC5572523 | uncharacterized protein LOC5572523 | 4.09 | 0.00 | 6.20 | 8.85 | 4.35 | 0.21 | 0.14 | 0.43 |
| gene-LOC5569171 | serpin B5 | 4.03 | 0.00 | 2.22 | 4.33 | 1.16 | 0.23 | 0.15 | 0.08 |
| gene-LOC5564141 | MD-2-related lipid-recognition protein | 3.98 | 0.00 | 1.89 | 7.17 | 2.49 | 0.09 | 0.18 | 0.45 |
| gene-LOC11068088 | uncharacterized protein LOC110678083 | 3.97 | 0.00 | 3.49 | 6.05 | 2.96 | 0.13 | 0.30 | 0.35 |
| gene-LOC5578630 | uncharacterized protein LOC5578630 | 3.94 | 0.00 | 22.54 | 37.69 | 11.68 | 1.85 | 1.48 | 1.27 |
| gene-LOC5575673 | glutamyl aminopeptidase | 3.93 | 0.02 | 0.51 | 0.75 | 0.17 | 0.07 | 0.00 | 0.02 |
| gene-LOC5568044 | probable uridine nucleosidase 1 | 3.92 | 0.00 | 21.33 | 39.13 | 11.69 | 1.07 | 2.64 | 0.98 |
| gene-LOC5575349 | tryptase | 3.86 | 0.00 | 1.97 | 3.35 | 0.98 | 0.14 | 0.07 | 0.14 |
| gene-LOC5564852 | neither inactivation nor afterpotential protein C | 3.85 | 0.00 | 2.16 | 3.18 | 1.50 | 0.13 | 0.29 | 0.06 |
| gene-LOC5578510 | trypsin 5G1 | 3.84 | 0.00 | 3.18 | 11.43 | 14.69 | 0.41 | 0.17 | 1.40 |
| gene-LOC11067712 | drosulfakinins | 3.84 | 0.00 | 5.80 | 6.59 | 2.96 | 0.19 | 0.36 | 0.51 |
| gene-LOC5566276 | ficolin-3 | 3.83 | 0.00 | 9.02 | 11.57 | 4.50 | 0.69 | 0.40 | 0.63 |
| gene-LOC5568133 | AAEL006576-PB; venom protease | 3.83 | 0.00 | 6.85 | 13.13 | 4.91 | 0.62 | 0.65 | 0.45 |
| gene-LOC5568251 | melanization protease 1 | 3.83 | 0.00 | 4.66 | 12.22 | 2.82 | 0.41 | 0.48 | 0.47 |
| gene-LOC5579999 | fatty acyl-CoA reductase wat | 3.82 | 0.00 | 0.72 | 2.47 | 1.00 | 0.07 | 0.07 | 0.15 |
| gene-LOC11068117 | uncharacterized protein LOC110681177 | 3.81 | 0.00 | 2.02 | 5.72 | 4.75 | 0.46 | 0.14 | 0.24 |

| | | | | | | | | | |
|---|---|---|---|---|---|---|---|---|---|
| gene-LOC5570039 | uncharacterized protein LOC109400458 isoform X2; uncharacterized protein LOC5570039 isoform X2 | 3.81 | 0.00 | 5.14 | 19.68 | 10.42 | 0.54 | 1.33 | 0.36 |
| gene-LOC5566497 | transient receptor potential protein isoform X2 | 3.80 | 0.00 | 0.84 | 0.77 | 0.49 | 0.11 | 0.04 | 0.00 |
| gene-LOC5578210 | uncharacterized protein LOC5578210 | 3.80 | 0.00 | 5.26 | 19.19 | 7.84 | 1.12 | 0.66 | 0.51 |
| gene-LOC5578208 | 27 kDa hemolymph protein | 3.80 | 0.01 | 2.76 | 5.29 | 2.04 | 0.00 | 0.71 | 0.00 |
| gene-LOC5572198 | opsin, ultraviolet-sensitive | 3.79 | 0.00 | 23.39 | 26.00 | 11.87 | 0.91 | 1.63 | 1.83 |
| gene-LOC5566698 | transient-receptor-potential-like protein | 3.78 | 0.00 | 11.16 | 12.08 | 5.06 | 0.81 | 0.37 | 0.86 |
| gene-LOC5566826 | adenosine deaminase 2 | 3.78 | 0.00 | 4.23 | 8.01 | 3.15 | 0.46 | 0.27 | 0.47 |
| gene-LOC5570482 | leucine-rich repeat transmembrane neuronal protein 3 | 3.77 | 0.00 | 4.63 | 6.90 | 2.99 | 0.34 | 0.36 | 0.35 |
| gene-LOC5570664 | 27 kDa hemolymph protein | 3.75 | 0.00 | 8.55 | 16.90 | 10.50 | 0.72 | 1.23 | 0.67 |
| gene-LOC5570483 | acidic leucine-rich nuclear phosphoprotein 32 family member | 3.74 | 0.00 | 5.90 | 13.88 | 4.36 | 0.45 | 0.90 | 0.42 |
| gene-LOC5577897 | argininosuccinate lyase | 3.73 | 0.00 | 11.49 | 43.56 | 12.41 | 0.91 | 2.77 | 1.33 |
| gene-LOC5569107 | uncharacterized protein LOC5569107 | 3.73 | 0.00 | 3.53 | 10.92 | 5.57 | 0.32 | 0.51 | 0.63 |
| gene-LOC5572020 | prosialokinin | 3.68 | 0.00 | 22.95 | 35.09 | 27.68 | 1.26 | 4.50 | 0.45 |
| gene-LOC5578507 | trypsin 5G1-like | 3.68 | 0.00 | 4.79 | 13.05 | 13.86 | 0.00 | 1.02 | 1.43 |
| gene-LOC11067483 | uncharacterized protein LOC110674832 | 3.66 | 0.00 | 13.61 | 17.18 | 4.73 | 1.09 | 1.15 | 0.46 |
| gene-LOC5575678 | venom protease isoform X1/X2 | 3.65 | 0.01 | 1.85 | 1.98 | 1.15 | 0.16 | 0.00 | 0.25 |
| gene-LOC11068125 | transient receptor potential protein-like isoform X1 | 3.65 | 0.00 | 1.99 | 1.96 | 0.74 | 0.10 | 0.18 | 0.11 |
| gene-LOC5566274 | uncharacterized protein LOC5566274 | 3.64 | 0.00 | 2.50 | 3.14 | 1.03 | 0.13 | 0.37 | 0.03 |
| gene-LOC5566409 | uncharacterized protein LOC5566409 | 3.62 | 0.05 | 0.02 | 0.93 | 0.64 | 0.04 | 0.00 | 0.08 |
| gene-LOC5565434 | leucine-rich repeat-containing protein 15 | 3.61 | 0.00 | 1.55 | 4.72 | 1.69 | 0.12 | 0.20 | 0.32 |

| | | | | | | | | | |
|---|---|---|---|---|---|---|---|---|---|
| gene-LOC5570480 | uncharacterized protein LOC5570480 | 3.61 | 0.00 | 15.86 | 35.41 | 15.87 | 2.10 | 2.44 | 1.06 |
| gene-LOC5563674 | perlucin | 3.60 | 0.00 | 9.15 | 20.28 | 6.51 | 0.47 | 1.28 | 1.17 |
| gene-LOC5575814 | uncharacterized protein LOC5575814 | 3.60 | 0.00 | 3.67 | 3.81 | 1.96 | 0.21 | 0.30 | 0.26 |
| gene-LOC5565674 | AAEL014937-PA; uncharacterized protein LOC5565674 | 3.57 | 0.00 | 5.57 | 48.66 | 23.97 | 2.40 | 1.15 | 1.77 |
| gene-LOC5579094 | defensin-C | 3.57 | 0.01 | 5.63 | 12.17 | 2.01 | 0.20 | 0.41 | 1.03 |
| gene-LOC5576017 | uncharacterized protein LOC5576017 | 3.55 | 0.01 | 0.81 | 0.98 | 1.77 | 0.19 | 0.10 | 0.00 |
| gene-LOC5569140 | uncharacterized protein LOC5569140 | 3.55 | 0.00 | 9.63 | 17.68 | 7.30 | 0.75 | 1.47 | 0.68 |
| gene-LOC11067473 | serine protease snake-like isoform X1-X4 | 3.50 | 0.00 | 4.11 | 6.22 | 3.10 | 0.38 | 0.62 | 0.18 |
| gene-LOC11067701 | uncharacterized protein LOC110677017 | 3.50 | 0.00 | 21.66 | 36.61 | 13.82 | 2.49 | 3.15 | 0.54 |
| gene-LOC5575552 | arginase, hepatic | 3.50 | 0.00 | 7.03 | 20.35 | 6.63 | 0.59 | 1.13 | 1.25 |
| gene-LOC5564669 | pancreatic lipase-related protein 2 | 3.49 | 0.00 | 0.75 | 2.46 | 1.21 | 0.11 | 0.22 | 0.06 |
| gene-LOC5573204 | chitinase-like protein Idgf4 | 3.49 | 0.00 | 9.06 | 21.76 | 16.32 | 1.34 | 1.22 | 1.55 |
| gene-LOC11068142 | ferrochelatase, mitochondrial | 3.47 | 0.00 | 0.22 | 11.20 | 10.17 | 0.20 | 0.86 | 0.20 |
| gene-LOC5566275 | uncharacterized protein LOC5566275 | 3.47 | 0.00 | 0.93 | 4.23 | 0.48 | 0.10 | 0.28 | 0.11 |
| novel4951 | hypothetical protein RP20_CCG025079; uncharacterized protein | 3.45 | 0.01 | 0.32 | 0.16 | 0.11 | 0.03 | 0.00 | 0.02 |
| gene-LOC5566582 | basement membrane-specific heparan sulfate proteoglycan core protein | 3.44 | 0.00 | 3.02 | 6.24 | 2.67 | 0.27 | 0.80 | 0.00 |
| gene-LOC11067808 | CUE domain-containing protein 2 | 3.43 | 0.02 | 0.93 | 2.01 | 2.73 | 1.49 | 0.44 | 0.00 |
| gene-LOC5577639 | carotenoid isomerooxygenase isoform X1/X2 | 3.42 | 0.00 | 2.32 | 3.02 | 1.36 | 0.15 | 0.25 | 0.25 |
| gene-LOC11067398 | mitochondrial basic amino acids transporter-like | 3.41 | 0.00 | 4.39 | 8.07 | 4.47 | 0.34 | 0.64 | 0.63 |
| gene-LOC5572681 | apolipophorins | 3.40 | 0.00 | 5.18 | 8.80 | 4.80 | 0.67 | 0.72 | 0.36 |
| gene-LOC5576151 | lipase 3 isoform X1/X2 | 3.39 | 0.00 | 3.24 | 4.77 | 4.30 | 0.33 | 0.37 | 0.83 |

| | | | | | | | | | |
|---|---|---|---|---|---|---|---|---|---|
| gene-LOC5563550 | serine protease easter | 3.39 | 0.00 | 1.08 | 2.54 | 1.22 | 0.12 | 0.13 | 0.21 |
| gene-LOC5568135 | AAEL014004-PA, partial; serine protease easter | 3.38 | 0.02 | 1.47 | 1.03 | 1.23 | 0.00 | 0.24 | 0.12 |
| gene-LOC11067529[illegible] | protein THEM6-like | 3.36 | 0.00 | 2.24 | 2.79 | 1.03 | 0.20 | 0.16 | 0.26 |
| gene-LOC5579410 | serine protease easter | 3.34 | 0.00 | 1.15 | 2.36 | 0.98 | 0.14 | 0.05 | 0.30 |
| gene-LOC5565037 | bombyxin B-1 homolog isoform X1/X2 | 3.33 | 0.00 | 4.20 | 7.25 | 3.52 | 0.49 | 0.86 | 0.09 |
| gene-LOC5576784 | glycine N-methyltransferase | 3.33 | 0.03 | 1.62 | 3.08 | 3.43 | 0.79 | 0.00 | 0.00 |
| gene-LOC5576659 | neither inactivation nor afterpotential protein G | 3.30 | 0.00 | 0.86 | 0.70 | 0.77 | 0.09 | 0.03 | 0.12 |
| gene-LOC5565479 | uncharacterized protein LOC5569108 | 3.29 | 0.00 | 2.95 | 5.83 | 2.22 | 0.00 | 0.60 | 0.51 |
| gene-LOC5577063 | uncharacterized protein LOC5577063 isoform X1-X3 | 3.27 | 0.00 | 3.03 | 3.61 | 1.57 | 0.07 | 0.42 | 0.45 |
| gene-LOC5569335 | AAEL007542-PA; cysteine sulfinic acid decarboxylase | 3.25 | 0.00 | 4.50 | 6.31 | 4.06 | 0.23 | 0.92 | 0.91 |
| gene-LOC5570678 | Golgi-associated plant pathogenesis-related protein 1 | 3.24 | 0.00 | 8.93 | 25.28 | 9.57 | 1.43 | 1.65 | 1.47 |
| novel2591 | - | 3.24 | 0.00 | 1.01 | 1.15 | 0.70 | 0.16 | 0.14 | 0.00 |
| gene-LOC5569396 | trypsin 5G1-like | 3.24 | 0.01 | 1.52 | 5.86 | 4.19 | 0.00 | 0.61 | 0.60 |
| novel379 | uncharacterized protein K02A2.6-like | 3.22 | 0.00 | 1.76 | 0.51 | 1.02 | 0.19 | 0.02 | 0.14 |
| gene-LOC5575174 | A-agglutinin anchorage subunit isoform X1-X3; AAEL000319-PA | 3.18 | 0.00 | 3.71 | 6.87 | 2.85 | 0.69 | 0.31 | 0.50 |
| gene-LOC5573025 | trypsin 5G1-like | 3.13 | 0.01 | 7.33 | 29.73 | 43.61 | 0.72 | 2.75 | 5.80 |
| gene-LOC5573677 | MAP kinase phosphatase with leucine-rich repeats protein 1 | 3.13 | 0.00 | 0.60 | 1.18 | 0.55 | 0.13 | 0.05 | 0.08 |
| gene-LOC5577059 | uncharacterized protein LOC5577059 | 3.07 | 0.00 | 4.11 | 3.57 | 1.66 | 0.29 | 0.74 | 0.06 |
| gene-LOC5572580 | uncharacterized protein LOC5572580 | 3.06 | 0.00 | 4.95 | 10.06 | 1.95 | 0.53 | 0.99 | 0.48 |
| gene-LOC11067777[illegible] | uncharacterized protein LOC110677777 | 3.04 | 0.01 | 5.84 | 17.14 | 4.92 | 1.32 | 0.20 | 1.79 |
| gene-LOC5580270 | glutamate-gated chloride channel isoform X1-X15 | 3.03 | 0.01 | 0.62 | 1.07 | 0.32 | 0.12 | 0.03 | 0.10 |

| | | | | | | | | | |
|---|---|---|---|---|---|---|---|---|---|
| gene-LOC5570404 | uncharacterized protein LOC5570404 | 3.01 | 0.00 | 28.31 | 44.32 | 18.72 | 4.13 | 2.89 | 4.17 |
| gene-LOC5565301 | protein BTG1 | 2.99 | 0.00 | 0.21 | 0.24 | 0.15 | 0.03 | 0.04 | 0.01 |
| gene-LOC5576429 | low density lipoprotein receptor adapter protein 1-A | 2.96 | 0.00 | 0.70 | 3.53 | 1.93 | 0.29 | 0.39 | 0.15 |
| gene-LOC5572603 | uncharacterized protein LOC5572603 | 2.95 | 0.00 | 1.64 | 3.92 | 1.17 | 0.13 | 0.35 | 0.39 |
| gene-LOC5578631 | uncharacterized protein LOC5578631 | 2.95 | 0.00 | 9.49 | 15.46 | 4.05 | 0.74 | 1.55 | 1.43 |
| gene-LOC5564575 | esterase B1 | 2.94 | 0.00 | 1.16 | 3.24 | 1.35 | 0.09 | 0.43 | 0.21 |
| gene-LOC5565436 | LOW QUALITY PROTEIN: alanine--glyoxylate aminotransferase 2, mitochondrial | 2.93 | 0.02 | 0.84 | 2.10 | 1.14 | 0.00 | 0.21 | 0.38 |
| gene-LOC5571025 | dynein assembly factor 1, axonemal homolog | 2.92 | 0.00 | 2.09 | 9.77 | 3.94 | 0.75 | 1.14 | 0.14 |
| gene-LOC5578211 | uncharacterized protein LOC5578211 | 2.91 | 0.01 | 1.41 | 3.03 | 1.42 | 0.11 | 0.53 | 0.12 |
| gene-LOC5579042 | uncharacterized protein LOC5579042 | 2.91 | 0.00 | 3.64 | 5.81 | 2.62 | 0.56 | 0.58 | 0.47 |
| gene-LOC5569168 | brachyurin | 2.91 | 0.00 | 2.64 | 6.61 | 3.47 | 0.16 | 0.32 | 1.20 |
| gene-LOC5573484 | uncharacterized protein LOC5573484 | 2.89 | 0.02 | 1.08 | 2.04 | 0.54 | 0.06 | 0.37 | 0.06 |
| gene-LOC5564510 | cytochrome b5-related protein | 2.87 | 0.00 | 9.50 | 19.79 | 10.05 | 1.84 | 1.83 | 1.52 |
| gene-LOC5569911 | clavesin-1 | 2.86 | 0.00 | 2.46 | 5.82 | 2.18 | 0.36 | 0.51 | 0.55 |
| gene-LOC5573084 | aminomethyltransferase, mitochondrial | 2.85 | 0.00 | 1.18 | 4.81 | 1.64 | 0.46 | 0.40 | 0.17 |
| gene-LOC5578506 | trypsin 5G1-like | 2.83 | 0.00 | 15.90 | 45.02 | 35.64 | 2.15 | 4.32 | 6.91 |
| gene-LOC5563617 | serine protease easter | 2.83 | 0.00 | 2.76 | 4.49 | 2.71 | 0.21 | 0.94 | 0.22 |
| gene-LOC11067613[illegible] | heat shock 70 kDa protein cognate 4-like | 2.82 | 0.00 | 2.09 | 3.79 | 2.58 | 0.42 | 0.43 | 0.36 |
| gene-LOC5574523 | cholesterol 7-desaturase | 2.81 | 0.00 | 2.02 | 2.90 | 1.92 | 0.25 | 0.36 | 0.35 |
| gene-LOC5570479 | leucine-rich repeat transmembrane protein FLRT3 isoform X2; leucine-rich repeat transmembrane neuronal protein 4 isoform X1 | 2.79 | 0.00 | 5.86 | 12.62 | 9.41 | 1.43 | 2.04 | 0.46 |

| | | | | | | | | | |
|---|---|---|---|---|---|---|---|---|---|
| gene-LOC5567872 | apyrase | 2.78 | 0.00 | 2.71 | 4.55 | 1.47 | 0.48 | 0.28 | 0.49 |
| gene-LOC5572585 | PDZ and LIM domain protein 7 isoform X2; uncharacterized protein LOC5572585 isoform X1 | 2.77 | 0.00 | 3.38 | 11.77 | 6.73 | 0.44 | 1.82 | 0.90 |
| gene-LOC5567310 | actin-1-like | 2.77 | 0.00 | 34.79 | 79.56 | 41.90 | 6.59 | 8.92 | 7.07 |
| gene-LOC5569443 | uncharacterized peptidase C1-like protein F26E4.3 | 2.75 | 0.00 | 3.60 | 6.18 | 3.08 | 0.56 | 0.38 | 0.93 |
| novel457 | - | 2.75 | 0.00 | 7.98 | 16.01 | 16.45 | 0.87 | 3.05 | 2.01 |
| gene-LOC5578050 | uncharacterized protein LOC5578050 | 2.74 | 0.00 | 9.37 | 12.09 | 5.76 | 1.11 | 1.81 | 1.09 |
| gene-LOC5568052 | calcitonin gene-related peptide type 1 receptor | 2.74 | 0.04 | 0.58 | 0.77 | 0.61 | 0.16 | 0.00 | 0.13 |
| gene-LOC5578266 | transcription factor AP-1 isoform X1/X2 | 2.73 | 0.00 | 8.55 | 17.09 | 11.44 | 2.25 | 2.17 | 1.06 |
| gene-LOC5569048 | lipopolysaccharide-induced tumor necrosis factor-alpha factor | 2.72 | 0.00 | 5.82 | 9.80 | 10.61 | 0.69 | 1.80 | 1.37 |
| gene-LOC5575764 | AAEL002720-PA; leukocyte elastase inhibitor | 2.72 | 0.00 | 3.60 | 8.57 | 3.91 | 0.72 | 0.84 | 0.83 |
| gene-LOC5563734 | uncharacterized protein LOC119766225; superoxide dismutase | 2.72 | 0.00 | 0.99 | 1.89 | 0.89 | 0.73 | 0.72 | 0.10 |
| gene-LOC5574170 | tryptase isoform X1/X2 | 2.71 | 0.02 | 2.01 | 5.74 | 2.63 | 0.00 | 0.92 | 0.63 |
| gene-LOC5569606 | uncharacterized protein LOC5569606 | 2.71 | 0.00 | 2.73 | 6.41 | 3.13 | 0.41 | 0.96 | 0.48 |
| gene-LOC5570086 | AAEL001343-PA; uncharacterized protein LOC5570086 | 2.68 | 0.00 | 1.86 | 3.36 | 1.81 | 0.44 | 0.42 | 0.25 |
| gene-LOC5571163 | uncharacterized protein LOC5571163 | 2.68 | 0.01 | 1.19 | 0.95 | 1.44 | 0.09 | 0.23 | 0.23 |
| gene-LOC5567040 | uncharacterized protein LOC5567040 | 2.68 | 0.00 | 9.40 | 6.44 | 3.95 | 0.67 | 1.29 | 1.05 |
| gene-LOC5573649 | uncharacterized protein LOC5573649 | 2.67 | 0.00 | 2.33 | 6.44 | 4.68 | 1.46 | 0.28 | 0.33 |
| gene-LOC5570982 | inositol oxygenase | 2.64 | 0.00 | 11.82 | 13.12 | 6.67 | 1.24 | 1.71 | 2.04 |
| gene-LOC5564764 | cytochrome P450 9e2 | 2.61 | 0.00 | 1.45 | 1.42 | 1.05 | 0.13 | 0.14 | 0.37 |
| gene-LOC5573929 | fatty acid synthase | 2.59 | 0.00 | 2.38 | 5.02 | 1.45 | 0.86 | 0.60 | 0.19 |

| | | | | | | | | | |
|---|---|---|---|---|---|---|---|---|---|
| gene-LOC5566581 | basement membrane proteoglycan isoform X1/X2 | 2.59 | 0.00 | 1.33 | 3.30 | 1.70 | 0.35 | 0.61 | 0.09 |
| gene-LOC5568426 | delta-1-pyrroline-5-carboxylate synthase | 2.58 | 0.00 | 8.55 | 16.57 | 9.06 | 1.68 | 2.22 | 1.79 |
| gene-LOC5570847 | 37 kDa salivary gland allergen Aed a 2-like | 2.58 | 0.00 | 2.75 | 4.75 | 3.35 | 0.41 | 0.54 | 0.85 |
| gene-LOC5570229 | fatty acid synthase | 2.57 | 0.00 | 1.77 | 3.31 | 1.46 | 0.39 | 0.34 | 0.35 |
| gene-LOC5574396 | sorbitol dehydrogenase | 2.57 | 0.03 | 1.37 | 3.62 | 1.15 | 0.08 | 0.81 | 0.13 |
| gene-LOC11067958[illegible] | myb-like protein V | 2.56 | 0.00 | 5.38 | 10.81 | 7.42 | 1.71 | 1.35 | 0.87 |
| gene-LOC5567194 | surface antigen CRP170 | 2.52 | 0.00 | 10.28 | 14.63 | 12.50 | 1.00 | 2.12 | 3.33 |
| gene-LOC5565231 | uncharacterized protein LOC5565231 | 2.52 | 0.02 | 2.27 | 4.51 | 0.91 | 0.61 | 0.22 | 0.51 |
| gene-LOC5571641 | laccase-2 | 2.48 | 0.00 | 9.22 | 9.77 | 6.98 | 0.84 | 2.58 | 1.78 |
| novel33 | - | 2.47 | 0.02 | 0.72 | 0.91 | 0.92 | 0.22 | 0.00 | 0.22 |
| gene-LOC5564219 | transient receptor potential channel pyrexia | 2.47 | 0.00 | 1.08 | 0.82 | 0.91 | 0.08 | 0.17 | 0.27 |
| gene-LOC5563675 | C-type lectin 37Db | 2.47 | 0.00 | 23.07 | 37.91 | 11.66 | 1.58 | 6.85 | 4.36 |
| novel2755 | - | 2.44 | 0.00 | 2.17 | 2.31 | 1.92 | 0.40 | 0.44 | 0.32 |
| gene-LOC5565763 | uncharacterized protein LOC5565763 | 2.42 | 0.00 | 0.95 | 0.80 | 0.75 | 0.20 | 0.16 | 0.10 |
| gene-LOC5574096 | purine nucleoside phosphorylase isoform X1/X2 | 2.40 | 0.00 | 5.38 | 10.54 | 6.15 | 0.79 | 1.30 | 1.69 |
| gene-LOC23687956 | holotricin-3 isoform X1-X3 | 2.40 | 0.00 | 27.42 | 71.68 | 40.65 | 6.14 | 8.97 | 11.05 |
| gene-LOC5577716 | protein lethal(2)essential for life | 2.39 | 0.00 | 6.52 | 12.80 | 8.31 | 1.75 | 2.31 | 1.11 |
| gene-LOC5564315 | facilitated trehalose transporter Tret1 | 2.38 | 0.00 | 8.61 | 7.93 | 1.26 | 1.17 | 1.80 | 0.43 |
| gene-LOC5576660 | uncharacterized protein LOC5576660 | 2.38 | 0.01 | 1.49 | 2.49 | 1.78 | 0.27 | 0.33 | 0.49 |
| novel555 | hypothetical protein RP20_CCG019945 | 2.37 | 0.02 | 0.15 | 0.29 | 0.24 | 0.02 | 0.07 | 0.04 |
| gene-LOC11067560[illegible] | uncharacterized protein LOC110675609 | 2.35 | 0.00 | 16.86 | 44.91 | 33.40 | 3.10 | 4.56 | 10.82 |
| gene-LOC5564668 | pancreatic lipase-related protein 2 | 2.33 | 0.02 | 0.90 | 3.87 | 2.04 | 0.09 | 0.55 | 0.70 |
| gene-LOC5570557 | leucine-rich repeat-containing protein 23 | 2.30 | 0.00 | 1.22 | 2.19 | 1.53 | 0.17 | 0.45 | 0.37 |

| | | | | | | | | | |
|---|---|---|---|---|---|---|---|---|---|
| gene-LOC5569282 | uncharacterized protein Dwil_GK26988, partial; dendritic arbor reduction protein 1 | 2.30 | 0.00 | 23.33 | 11.48 | 9.21 | 3.92 | 2.27 | 2.02 |
| gene-LOC5564672 | 2-oxo-4-hydroxy-4-carboxy-5-ureidoimidazoline decarboxylase | 2.30 | 0.00 | 3.28 | 8.20 | 4.42 | 0.58 | 1.01 | 1.60 |
| gene-LOC5571297 | 2-hydroxyacylsphingosine 1-beta-galactosyltransferase | 2.29 | 0.00 | 3.16 | 4.01 | 1.54 | 0.58 | 0.62 | 0.55 |
| gene-LOC5575325 | transmembrane protease serine 2 | 2.29 | 0.01 | 1.19 | 1.84 | 0.63 | 0.32 | 0.15 | 0.31 |
| gene-LOC5566558 | macrophage mannose receptor 1 | 2.28 | 0.02 | 3.43 | 13.38 | 6.50 | 1.20 | 3.21 | 0.69 |
| gene-LOC5573927 | fatty acid synthase | 2.28 | 0.00 | 1.99 | 4.97 | 1.56 | 0.41 | 0.95 | 0.37 |
| gene-LOC5567003 | serine protease snake isoform X1/X2 | 2.27 | 0.02 | 2.25 | 3.97 | 1.18 | 0.46 | 0.91 | 0.16 |
| gene-LOC5572111 | probable maltase | 2.27 | 0.00 | 124.68 | 228.66 | 97.76 | 36.50 | 34.84 | 22.06 |
| gene-LOC5575021 | uncharacterized protein LOC5575021 isoform X1-X4; AAEL011574-PA | 2.27 | 0.00 | 4.23 | 5.00 | 2.62 | 0.73 | 0.44 | 0.89 |
| gene-LOC5573312 | uncharacterized protein LOC5573312 | 2.26 | 0.00 | 8.66 | 10.75 | 10.78 | 1.15 | 2.12 | 2.87 |
| gene-LOC5572848 | putative uncharacterized protein DDB_G0277255 | 2.26 | 0.00 | 1.48 | 2.68 | 1.44 | 0.42 | 0.56 | 0.17 |
| gene-LOC5567955 | putative 18.2 kDa secreted protein; uncharacterized protein LOC5567955 | 2.25 | 0.00 | 10.96 | 27.83 | 11.70 | 3.02 | 4.67 | 2.98 |
| gene-LOC5575017 | hemicentin-1 | 2.25 | 0.01 | 0.29 | 0.55 | 0.32 | 0.04 | 0.15 | 0.05 |
| gene-LOC5568681 | pyrroline-5-carboxylate reductase | 2.25 | 0.02 | 1.20 | 3.10 | 2.44 | 0.40 | 1.20 | 0.21 |
| gene-LOC5564209 | fatty-acid amide hydrolase 2 | 2.24 | 0.00 | 1.53 | 3.01 | 1.89 | 0.29 | 0.60 | 0.45 |
| gene-LOC5568624 | uncharacterized protein LOC5568624 | 2.24 | 0.00 | 6.79 | 14.27 | 6.16 | 1.59 | 2.45 | 1.64 |
| gene-LOC5570893 | AAEL001504-PA; dual specificity tyrosine-phosphorylation-regulated kinase 2 isoform X3/X4/X5/X6/X7; dual specificity tyrosine-phosphorylation-regulated kinase 4 isoform X1/X2 | 2.22 | 0.00 | 7.48 | 4.42 | 4.35 | 1.18 | 0.98 | 1.39 |
| gene-LOC5572380 | cytochrome P450 307a1 | 2.22 | 0.00 | 1.76 | 3.91 | 1.86 | 0.41 | 0.38 | 1.10 |

| | | | | | | | | | |
|---|---|---|---|---|---|---|---|---|---|
| gene-LOC5567779 | luciferin 4-monooxygenase | 2.22 | 0.00 | 0.92 | 1.69 | 1.05 | 0.17 | 0.36 | 0.25 |
| gene-LOC5574712 | dnaJ homolog subfamily C member 22 | 2.21 | 0.03 | 1.73 | 1.96 | 0.90 | 0.55 | 0.31 | 0.13 |
| gene-LOC5569163 | cysteine dioxygenase type 1 | 2.21 | 0.00 | 2.89 | 3.92 | 1.58 | 0.34 | 0.79 | 0.68 |
| gene-LOC5566353 | D-3-phosphoglycerate dehydrogenase | 2.20 | 0.00 | 4.41 | 9.58 | 5.61 | 0.80 | 1.93 | 1.49 |
| gene-LOC5571325 | tyrosine aminotransferase | 2.19 | 0.00 | 4.08 | 5.48 | 3.83 | 0.90 | 1.15 | 0.82 |
| gene-LOC5580054 | uncharacterized protein LOC5580054 isoform X1-X3 | 2.19 | 0.00 | 0.57 | 0.47 | 0.46 | 0.15 | 0.13 | 0.06 |
| gene-LOC5571667 | uncharacterized protein LOC5571667 isoform X1-X2 | 2.17 | 0.00 | 4.39 | 10.85 | 5.35 | 1.54 | 1.44 | 1.54 |
| gene-LOC5567327 | disintegrin and metalloproteinase domain-containing protein 12 | 2.17 | 0.00 | 1.67 | 2.35 | 1.48 | 0.19 | 0.50 | 0.52 |
| gene-LOC5571141 | probable cytochrome P450 9f2 isoform X2 | 2.17 | 0.00 | 3.54 | 4.22 | 4.19 | 1.16 | 0.68 | 0.70 |
| gene-LOC5564618 | protein-glucosylgalactosylhydroxylysine glucosidase | 2.17 | 0.00 | 2.18 | 4.12 | 1.56 | 0.42 | 0.73 | 0.58 |
| gene-LOC5579359 | PAX-interacting protein 1 | 2.16 | 0.02 | 0.74 | 1.31 | 0.47 | 0.25 | 0.14 | 0.16 |
| gene-LOC5567672 | uncharacterized protein LOC5567672 | 2.16 | 0.00 | 1.37 | 2.90 | 1.86 | 0.41 | 0.49 | 0.45 |
| gene-LOC5571316 | LOW QUALITY PROTEIN: | 2.15 | 0.01 | 0.87 | 0.89 | 0.97 | 0.09 | 0.14 | 0.43 |
| gene-LOC5576459 | uncharacterized protein LOC5576459 | 2.15 | 0.01 | 5.39 | 19.30 | 8.69 | 1.17 | 3.69 | 2.45 |
| gene-LOC5571390 | putative serine protease F56F10.1 | 2.15 | 0.00 | 3.87 | 4.61 | 4.84 | 1.00 | 0.72 | 1.24 |
| gene-LOC5579192 | beta-1,3-glucan-binding protein | 2.15 | 0.01 | 1.22 | 1.88 | 1.62 | 0.30 | 0.55 | 0.19 |
| gene-LOC5572722 | uncharacterized protein LOC5572722 | 2.15 | 0.00 | 2.79 | 3.16 | 1.31 | 0.36 | 0.84 | 0.42 |
| novel5027 | - | 2.15 | 0.00 | 17.08 | 29.56 | 30.32 | 4.08 | 4.86 | 8.10 |
| gene-LOC5567899 | hybrid signal transduction histidine kinase B | 2.15 | 0.00 | 1.70 | 1.84 | 1.03 | 0.28 | 0.50 | 0.43 |
| gene-LOC5573537 | polypeptide N-acetylgalactosaminyltransferase 16 | 2.14 | 0.00 | 2.46 | 2.95 | 1.69 | 0.64 | 0.41 | 0.55 |
| gene-LOC5575917 | ATP-binding cassette sub-family G member 5 | 2.13 | 0.01 | 0.98 | 1.30 | 0.94 | 0.14 | 0.26 | 0.32 |

| | | | | | | | | | |
|---|---|---|---|---|---|---|---|---|---|
| gene-LOC5573254 | alpha-tocopherol transfer protein isoform X1/X2 | 2.10 | 0.00 | 2.40 | 4.24 | 3.33 | 0.87 | 0.33 | 1.10 |
| gene-LOC5578720 | probable multidrug resistance-associated protein lethal(2)03659 | 2.10 | 0.02 | 0.27 | 0.60 | 0.32 | 0.10 | 0.06 | 0.11 |
| gene-LOC5574469 | esterase B1 | 2.10 | 0.04 | 0.52 | 1.05 | 0.88 | 0.13 | 0.30 | 0.20 |
| gene-LOC5578672 | sodium-dependent nutrient amino acid transporter 1 isoform X1/X2 | 2.09 | 0.00 | 2.49 | 4.02 | 1.75 | 0.96 | 0.42 | 0.52 |
| gene-LOC5579241 | transmembrane protein 35A | 2.08 | 0.00 | 2.71 | 5.18 | 3.70 | 1.37 | 0.58 | 0.70 |
| gene-LOC5567015 | lysosomal alpha-mannosidase | 2.07 | 0.00 | 1.71 | 3.05 | 2.27 | 0.53 | 0.35 | 0.77 |
| gene-LOC5575393 | venom allergen 5 | 2.06 | 0.02 | 2.87 | 6.32 | 4.73 | 0.54 | 1.78 | 0.93 |
| gene-LOC5572218 | estradiol 17-beta-dehydrogenase | 2.06 | 0.00 | 3.38 | 11.17 | 6.57 | 1.29 | 1.60 | 2.09 |
| gene-LOC5568295 | alpha-amylase I | 2.06 | 0.00 | 12.80 | 24.84 | 13.29 | 4.65 | 3.67 | 3.69 |
| gene-LOC5567584 | general odorant-binding protein | 2.06 | 0.00 | 9.97 | 15.15 | 7.81 | 2.20 | 2.51 | 3.01 |
| gene-LOC5564054 | UNC93-like protein | 2.06 | 0.00 | 3.04 | 7.21 | 3.04 | 0.87 | 1.11 | 1.17 |
| gene-LOC5568132 | troponin C isoform X1-X3 | 2.04 | 0.00 | 39.18 | 92.27 | 58.94 | 14.45 | 19.34 | 15.09 |
| gene-LOC5577569 | collagenase | 2.03 | 0.00 | 13.09 | 9.24 | 16.04 | 3.61 | 5.28 | 8.76 |
| gene-LOC5572986 | actin, cytoplasmic 2 | 2.03 | 0.00 | 11.08 | 20.59 | 10.59 | 4.33 | 2.97 | 2.84 |
| gene-LOC5576672 | ATP-binding cassette sub-family A member 3 | 2.03 | 0.00 | 8.63 | 12.42 | 10.25 | 1.92 | 2.91 | 2.83 |
| gene-LOC5569815 | clavesin-1 | 2.03 | 0.02 | 1.42 | 2.09 | 1.01 | 0.30 | 0.55 | 0.27 |
| gene-LOC5573257 | alpha-tocopherol transfer protein | 2.03 | 0.04 | 0.38 | 1.17 | 1.14 | 0.23 | 0.36 | 0.08 |
| gene-LOC5568781 | facilitated trehalose transporter Tret1 | 2.02 | 0.03 | 1.06 | 1.89 | 0.83 | 0.31 | 0.32 | 0.28 |
| gene-LOC5563663 | serine protease easter | 2.02 | 0.01 | 2.00 | 5.13 | 2.77 | 0.58 | 0.71 | 0.92 |
| gene-LOC5566990 | AAEL005733-PA; LOW QUALITY PROTEIN: myosin heavy chain, muscle | 2.02 | 0.00 | 33.66 | 44.59 | 29.37 | 8.06 | 8.64 | 9.44 |
| gene-LOC5567446 | gelsolin | 2.02 | 0.00 | 20.03 | 31.67 | 13.45 | 2.82 | 7.97 | 5.20 |
| gene-LOC5564456 | dual specificity protein phosphatase 18 | 2.02 | 0.00 | 1.30 | 1.95 | 1.55 | 0.48 | 0.31 | 0.37 |
| gene-LOC5569474 | uncharacterized protein LOC5569474 | 2.02 | 0.00 | 3.19 | 8.85 | 3.19 | 1.09 | 0.80 | 1.84 |
| gene-LOC5568136 | serine protease easter | 2.01 | 0.03 | 1.71 | 5.04 | 2.09 | 0.81 | 0.92 | 0.53 |
| gene-LOC5564658 | uncharacterized protein LOC5564658 | 2.00 | 0.00 | 19.16 | 32.06 | 16.79 | 5.95 | 5.59 | 5.16 |

| | | | | | | | | | |
|---|---|---|---|---|---|---|---|---|---|
| gene-LOC5568623 | uncharacterized protein LOC5568623 | 2.00 | 0.04 | 1.32 | 1.61 | 1.51 | 0.39 | 0.35 | 0.35 |
| gene-LOC11067970 | serine protease 7-like isoform | 2.00 | 0.00 | 5.30 | 10.81 | 4.70 | 1.04 | 2.36 | 1.75 |
| gene-LOC5579630 | arrestin domain-containing protein 17 | 2.00 | 0.04 | 0.74 | 1.24 | 1.08 | 0.23 | 0.24 | 0.28 |
| gene-LOC5568166 | Krueppel-like factor 3 | 1.99 | 0.00 | 18.44 | 25.17 | 15.46 | 4.00 | 5.29 | 5.38 |
| gene-LOC23687754 | flocculation protein FLO11 | 1.99 | 0.00 | 11.05 | 25.45 | 9.95 | 2.34 | 3.64 | 5.61 |
| gene-LOC5569630 | uncharacterized protein LOC5569630 | 1.98 | 0.00 | 9.52 | 18.90 | 12.88 | 2.74 | 4.72 | 2.76 |
| gene-LOC5576245 | tubulin beta-3 chain | 1.98 | 0.00 | 1.50 | 2.47 | 0.97 | 0.30 | 0.58 | 0.35 |
| gene-LOC5576475 | uncharacterized protein LOC5576475 | 1.98 | 0.00 | 2.91 | 5.96 | 4.46 | 1.15 | 0.88 | 1.30 |
| gene-LOC11067617 | uncharacterized protein LOC110676173 | 1.97 | 0.02 | 3.89 | 4.67 | 2.61 | 1.35 | 0.83 | 0.58 |
| gene-LOC5572116 | Niemann-Pick type protein homolog 1B | 1.97 | 0.01 | 0.80 | 1.94 | 1.72 | 0.17 | 0.23 | 0.72 |
| gene-LOC5567033 | glucose dehydrogenase | 1.97 | 0.00 | 1.81 | 2.08 | 1.00 | 0.51 | 0.42 | 0.34 |
| gene-LOC5572540 | uncharacterized protein LOC5572540 | 1.97 | 0.03 | 0.84 | 0.71 | 1.17 | 0.31 | 0.17 | 0.20 |
| gene-LOC5575071 | serine protease snake isoform X1/X2 | 1.96 | 0.01 | 2.12 | 3.22 | 3.42 | 0.63 | 0.90 | 1.05 |
| gene-LOC5576417 | uncharacterized protein LOC5576417 | 1.96 | 0.00 | 1.33 | 0.93 | 0.49 | 0.16 | 0.31 | 0.18 |
| gene-LOC5566267 | troponin C | 1.95 | 0.00 | 23.21 | 60.10 | 29.22 | 10.12 | 10.30 | 8.11 |
| gene-LOC5570220 | arrestin domain-containing protein | 1.95 | 0.01 | 1.98 | 4.96 | 2.35 | 0.41 | 1.16 | 0.80 |
| gene-LOC5565336 | probable cytochrome P450 4ac1; AAEL014019-PB | 1.94 | 0.00 | 3.27 | 6.60 | 3.20 | 1.39 | 1.43 | 1.04 |
| novel2139 | - | 1.93 | 0.00 | 2.12 | 2.06 | 2.10 | 0.31 | 0.65 | 0.68 |
| gene-LOC5566493 | protein mesh isoform X1-X3 | 1.92 | 0.00 | 15.95 | 20.78 | 16.13 | 3.44 | 5.20 | 5.14 |
| gene-LOC11068124 | 39S ribosomal protein L40, mitochondrial-like | 1.91 | 0.02 | 5.39 | 6.31 | 10.36 | 0.63 | 3.16 | 1.89 |
| novel368 | - | 1.91 | 0.01 | 0.94 | 1.13 | 7.08 | 0.45 | 0.20 | 0.63 |
| gene-LOC5574512 | inactive hydroxysteroid dehydrogenase-like protein 1 | 1.91 | 0.01 | 2.21 | 4.19 | 2.75 | 0.87 | 0.99 | 0.95 |
| gene-LOC5570223 | arrestin domain-containing protein | 1.90 | 0.02 | 1.85 | 1.61 | 2.23 | 0.58 | 0.23 | 0.65 |

| | | | | | | | | | |
|---|---|---|---|---|---|---|---|---|---|
| gene-LOC5578303 | pyruvate dehydrogenase E1 component subunit alpha, mitochondrial | 1.90 | 0.00 | 10.48 | 19.50 | 11.91 | 2.67 | 4.79 | 3.62 |
| gene-LOC5571588 | beta-1,3-glucan-binding protein | 1.89 | 0.00 | 8.46 | 7.97 | 8.95 | 1.38 | 2.99 | 2.35 |
| gene-LOC5571279 | peptidyl-alpha-hydroxyglycine alpha-amidating lyase 1 | 1.89 | 0.00 | 2.10 | 3.15 | 2.50 | 0.30 | 0.99 | 0.84 |
| novel407 | - | 1.89 | 0.02 | 0.35 | 0.55 | 0.26 | 0.10 | 0.14 | 0.09 |
| gene-LOC5566270 | 3 beta-hydroxysteroid dehydrogenase/Delta 5-->4-isomerase; AAEL000733-PB | 1.88 | 0.04 | 1.90 | 1.57 | 1.42 | 0.71 | 0.30 | 0.24 |
| gene-LOC5575489 | uncharacterized protein LOC5575489 | 1.88 | 0.01 | 0.50 | 0.70 | 0.56 | 0.07 | 0.20 | 0.20 |
| gene-LOC11067908[illegible] | neurofilament light polypeptide-like | 1.88 | 0.00 | 18.16 | 29.52 | 13.61 | 7.45 | 6.19 | 2.46 |
| gene-LOC5575341 | general odorant-binding protein | 1.87 | 0.04 | 5.96 | 10.76 | 4.75 | 3.63 | 1.39 | 0.64 |
| gene-LOC5575399 | venom allergen 5 | 1.87 | 0.01 | 5.19 | 8.65 | 3.53 | 0.92 | 2.36 | 1.38 |
| gene-LOC5571151 | sarcoplasmic calcium-binding protein 1 | 1.87 | 0.00 | 38.84 | 70.72 | 52.55 | 13.97 | 17.22 | 10.86 |
| gene-LOC5575338 | leucine-rich repeat neuronal protein 1 | 1.87 | 0.00 | 2.63 | 5.46 | 3.75 | 0.89 | 1.42 | 0.88 |
| gene-LOC5571916 | putative helicase MOV-10 | 1.86 | 0.00 | 1.80 | 1.72 | 2.99 | 0.70 | 0.69 | 0.28 |
| gene-LOC5574991 | glutamate receptor ionotropic, kainate 2 | 1.86 | 0.00 | 1.42 | 3.08 | 1.84 | 0.35 | 0.57 | 0.81 |
| gene-LOC5569810 | uncharacterized protein LOC5569810 isoform X1/X2 | 1.86 | 0.00 | 37.84 | 45.55 | 19.36 | 9.39 | 13.18 | 7.67 |
| gene-LOC5571053 | serine protease SP24D | 1.85 | 0.00 | 25.75 | 56.72 | 38.03 | 6.93 | 7.32 | 18.54 |
| gene-LOC5565448 | protein flightless-1 homolog | 1.85 | 0.01 | 2.18 | 2.11 | 2.20 | 0.59 | 0.88 | 0.30 |
| gene-LOC5571443 | uncharacterized protein LOC5571443 | 1.85 | 0.00 | 105.50 | 172.39 | 92.24 | 41.59 | 36.26 | 22.88 |
| gene-LOC5572428 | AAEL001794-PA/PB, partial; CD109 antigen | 1.85 | 0.00 | 8.73 | 4.32 | 4.00 | 0.68 | 0.94 | 0.56 |
| gene-LOC5576664 | sodium-coupled monocarboxylate transporter 1 | 1.85 | 0.02 | 1.13 | 1.20 | 0.74 | 0.23 | 0.37 | 0.39 |
| gene-LOC5578871 | uncharacterized protein LOC5578871 | 1.84 | 0.03 | 0.61 | 1.72 | 1.71 | 0.57 | 0.32 | 0.21 |
| gene-LOC11067717[illegible] | tektin-3-like isoform X1-X3 | 1.84 | 0.02 | 1.31 | 3.39 | 1.48 | 0.71 | 0.37 | 0.60 |

| | | | | | | | | | |
|---|---|---|---|---|---|---|---|---|---|
| gene-LOC5577194 | hypothetical protein ZHAS_00000797; AAEL014278-PA | 1.84 | 0.01 | 1.76 | 4.55 | 1.59 | 0.78 | 0.82 | 0.49 |
| gene-LOC5578897 | aminopeptidase N | 1.84 | 0.01 | 1.11 | 2.06 | 1.58 | 0.22 | 0.67 | 0.42 |
| gene-LOC5569637 | leucine-rich repeat-containing G-protein coupled receptor 4 | 1.83 | 0.00 | 7.21 | 12.69 | 6.73 | 2.63 | 3.20 | 1.52 |
| gene-LOC11067826 | putative protein TPRXL | 1.83 | 0.03 | 4.64 | 7.49 | 7.71 | 0.66 | 2.88 | 1.92 |
| gene-LOC5566572 | zinc transporter ZIP2 | 1.82 | 0.03 | 3.64 | 2.78 | 2.46 | 0.62 | 0.57 | 1.27 |
| gene-LOC5564497 | uncharacterized protein LOC5564497 | 1.82 | 0.00 | 18.51 | 22.59 | 14.03 | 4.73 | 7.11 | 3.34 |
| gene-LOC5579172 | polyubiquitin | 1.82 | 0.00 | 239.71 | 299.13 | 278.02 | 67.16 | 102.94 | 77.36 |
| gene-LOC5577358 | major facilitator superfamily domain-containing protein 6 | 1.82 | 0.00 | 0.89 | 0.84 | 0.54 | 0.17 | 0.29 | 0.18 |
| gene-LOC5570300 | uncharacterized protein LOC5570300 | 1.81 | 0.04 | 1.39 | 1.74 | 1.18 | 0.56 | 0.09 | 0.44 |
| gene-LOC5568783 | facilitated trehalose transporter Tret1 | 1.81 | 0.02 | 1.47 | 3.86 | 2.08 | 0.32 | 0.73 | 1.04 |
| gene-LOC5572891 | phosphoglycolate phosphatase 1B, chloroplastic | 1.81 | 0.00 | 4.23 | 7.31 | 6.21 | 1.36 | 2.27 | 1.33 |
| gene-LOC5578236 | AAEL013574-PB/PA; apolipoprotein D | 1.80 | 0.00 | 18.08 | 32.76 | 15.77 | 4.52 | 7.32 | 7.74 |
| gene-LOC5567907 | uncharacterized protein LOC5567907 | 1.80 | 0.01 | 2.71 | 6.66 | 3.67 | 1.18 | 0.96 | 1.34 |
| gene-LOC5571384 | uncharacterized protein LOC5571384 isoform X1; dentin sialophosphoprotein isoform X2 | 1.80 | 0.04 | 0.83 | 1.07 | 0.90 | 0.46 | 0.10 | 0.22 |
| gene-LOC5579486 | chymotrypsin-2 | 1.80 | 0.02 | 2.46 | 3.67 | 3.59 | 1.33 | 0.73 | 0.65 |
| gene-LOC5575669 | UDP-glucuronosyltransferase 2B37 | 1.79 | 0.01 | 2.11 | 1.88 | 2.52 | 0.49 | 1.12 | 0.33 |
| gene-LOC5573728 | low-density lipoprotein receptor-related protein 4 isoform X1/X2 | 1.79 | 0.00 | 0.66 | 0.89 | 0.43 | 0.21 | 0.22 | 0.13 |
| gene-LOC5573170 | ejaculatory bulb-specific protein 3 isoform X1/X2 | 1.78 | 0.00 | 66.90 | 109.57 | 53.62 | 17.97 | 20.98 | 25.16 |
| gene-LOC5579954 | mite group 2 allergen Gly d 2.01 | 1.78 | 0.02 | 8.94 | 21.04 | 15.55 | 2.86 | 2.48 | 7.59 |
| gene-LOC5565788 | facilitated trehalose transporter Tret1 | 1.77 | 0.00 | 5.39 | 9.79 | 8.70 | 2.37 | 2.40 | 2.13 |

| | | | | | | | | | |
|---|---|---|---|---|---|---|---|---|---|
| gene-LOC5576084 | 2-amino-3-ketobutyrate coenzyme A ligase, mitochondrial | 1.77 | 0.00 | 4.54 | 9.88 | 4.65 | 1.19 | 2.06 | 2.29 |
| gene-LOC5578664 | AAEL003626-PA; sodium-dependent nutrient amino acid | 1.76 | 0.00 | 4.96 | 7.07 | 4.20 | 2.01 | 1.34 | 1.35 |
| novel429 | - | 1.76 | 0.01 | 20.20 | 35.44 | 35.39 | 4.73 | 16.27 | 3.54 |
| gene-LOC5575658 | venom allergen 5.02 | 1.76 | 0.00 | 16.52 | 31.63 | 19.91 | 7.48 | 6.88 | 5.28 |
| gene-LOC5568175 | uncharacterized protein LOC5568175 | 1.76 | 0.00 | 3.62 | 6.40 | 4.29 | 0.87 | 1.60 | 1.71 |
| gene-LOC5568369 | synaptic vesicle glycoprotein 2B | 1.75 | 0.00 | 6.49 | 7.31 | 5.58 | 1.54 | 2.02 | 2.10 |
| gene-LOC5579043 | uncharacterized protein LOC119766220;uncharacterized protein LOC5579043 | 1.75 | 0.00 | 24.51 | 28.19 | 20.04 | 6.79 | 6.81 | 7.29 |
| gene-LOC5574272 | voltage-dependent calcium channel subunit alpha-2/delta-3 isoform X1-X3 | 1.75 | 0.02 | 0.50 | 0.72 | 0.42 | 0.14 | 0.12 | 0.22 |
| gene-LOC23687443 | AAEL017023-PA, partial; CD109 antigen | 1.75 | 0.01 | 1.09 | 1.63 | 0.65 | 0.37 | 0.34 | 0.21 |
| gene-LOC11067561 | uncharacterized protein LOC110675611 | 1.74 | 0.00 | 16.54 | 29.00 | 21.99 | 5.57 | 6.45 | 7.84 |
| gene-LOC5571382 | probable cytochrome P450 6a14 | 1.74 | 0.00 | 44.43 | 68.51 | 31.33 | 12.39 | 17.30 | 12.92 |
| gene-LOC5565843 | multidrug resistance-associated protein 1 | 1.73 | 0.04 | 0.35 | 0.43 | 0.35 | 0.09 | 0.14 | 0.11 |
| gene-LOC5570772 | actin-binding Rho-activating protein isoform X1-X3 | 1.73 | 0.01 | 1.77 | 2.89 | 1.39 | 1.00 | 0.47 | 0.59 |
| novel2571 | AAEL003593-PA | 1.73 | 0.02 | 4.30 | 3.36 | 1.88 | 1.03 | 0.62 | 0.68 |
| gene-LOC5574054 | ADP,ATP carrier protein 2 | 1.73 | 0.00 | 8.76 | 21.60 | 13.30 | 4.50 | 4.12 | 4.30 |
| gene-LOC5565890 | excitatory amino acid transporter isoform X1 | 1.72 | 0.00 | 2.57 | 4.26 | 2.26 | 0.62 | 0.89 | 1.28 |
| gene-LOC5569194 | protein sister of odd and bowel | 1.71 | 0.01 | 1.47 | 1.50 | 1.13 | 0.31 | 0.32 | 0.61 |
| gene-LOC5566732 | trypsin epsilon | 1.71 | 0.00 | 4.21 | 10.37 | 6.18 | 1.84 | 2.62 | 1.77 |
| gene-LOC11068113 | hypothetical protein RP20_CCG017660; protein-cysteine N-palmitoyltransferase Rasp isoform X1/X2 | 1.71 | 0.01 | 6.94 | 8.20 | 5.55 | 1.81 | 1.32 | 3.93 |

| | | | | | | | | | |
|---|---|---|---|---|---|---|---|---|---|
| gene-LOC5570528 | hydroxysteroid dehydrogenase-like protein 2 | 1.70 | 0.02 | 4.84 | 6.40 | 5.33 | 0.62 | 2.45 | 1.92 |
| gene-LOC5569903 | uncharacterized protein LOC5569903 | 1.70 | 0.00 | 308.23 | 580.74 | 305.90 | 141.85 | 130.91 | 83.79 |
| gene-LOC5572631 | carboxypeptidase B | 1.69 | 0.00 | 3.44 | 6.87 | 4.95 | 1.30 | 1.44 | 1.91 |
| gene-LOC5573987 | uncharacterized protein LOC5573987 isoform X1-X11 | 1.68 | 0.00 | 7.39 | 13.52 | 9.54 | 2.12 | 3.83 | 2.14 |
| gene-LOC5572437 | chondroitin sulfate proteoglycan 4 | 1.68 | 0.03 | 0.47 | 0.85 | 0.25 | 0.19 | 0.12 | 0.17 |
| gene-LOC5576799 | uncharacterized protein LOC6032053; aminopeptidase N | 1.68 | 0.00 | 2.44 | 3.24 | 2.44 | 0.31 | 0.74 | 0.51 |
| gene-LOC5575706 | L-lactate dehydrogenase | 1.68 | 0.00 | 2.68 | 4.70 | 3.95 | 0.90 | 1.04 | 1.66 |
| gene-LOC5573843 | uncharacterized protein LOC5573843 | 1.67 | 0.00 | 1.55 | 2.20 | 1.07 | 0.43 | 0.73 | 0.33 |
| gene-LOC5569805 | protein takeout isoform X1/X2 | 1.67 | 0.01 | 3.90 | 7.94 | 4.58 | 1.47 | 2.32 | 1.27 |
| gene-LOC5572213 | poly(U)-specific endoribonuclease homolog | 1.67 | 0.00 | 13.42 | 22.37 | 12.73 | 4.04 | 5.51 | 5.50 |
| gene-LOC5568744 | pyridoxal phosphate phosphatase isoform X1/X2 | 1.67 | 0.03 | 3.73 | 1.82 | 3.26 | 0.82 | 0.77 | 1.09 |
| gene-LOC5579205 | allantoicase | 1.67 | 0.03 | 3.51 | 3.22 | 2.08 | 0.42 | 1.14 | 1.18 |
| gene-LOC11067629[illegible] | fibroin heavy chain | 1.67 | 0.01 | 0.98 | 0.80 | 0.71 | 0.14 | 0.32 | 0.25 |
| gene-LOC5574953 | uncharacterized protein LOC5574953 | 1.67 | 0.00 | 15.89 | 21.62 | 14.08 | 4.36 | 6.08 | 5.61 |
| novel9 | - | 1.66 | 0.02 | 0.47 | 0.70 | 0.34 | 0.25 | 0.16 | 0.07 |
| gene-LOC5574120 | protein G12 | 1.66 | 0.03 | 5.90 | 13.74 | 8.82 | 1.09 | 3.94 | 3.80 |
| novel5021 | putative salivary secreted peptide | 1.66 | 0.00 | 10.44 | 35.03 | 14.77 | 5.65 | 7.91 | 5.12 |
| gene-LOC5578728 | uncharacterized protein LOC5578728 | 1.66 | 0.00 | 1.87 | 4.77 | 2.54 | 0.61 | 1.14 | 0.96 |
| gene-LOC5570040 | Vago protein; uncharacterized protein LOC5570040 | 1.66 | 0.00 | 34.43 | 75.37 | 42.34 | 14.57 | 20.19 | 11.10 |
| gene-LOC5578011 | phospholipid phosphatase 5 | 1.65 | 0.00 | 4.26 | 5.89 | 5.55 | 0.94 | 1.49 | 2.49 |
| gene-LOC5579903 | regucalcin isoform X1/X2 | 1.65 | 0.00 | 9.08 | 13.81 | 9.24 | 3.05 | 4.08 | 3.03 |
| gene-LOC11067615[illegible] | loricrin-like | 1.65 | 0.01 | 7.37 | 9.31 | 5.99 | 1.41 | 1.82 | 3.89 |

| | | | | | | | | | |
|---|---|---|---|---|---|---|---|---|---|
| gene-LOC5577938 | AAEL002552-PA, partial; hypothetical protein RP20_CCG025541; uncharacterized protein | 1.65 | 0.03 | 0.47 | 0.54 | 0.49 | 0.20 | 0.10 | 0.23 |
| gene-LOC5571541 | cytochrome P450 6d3 | 1.64 | 0.02 | 1.77 | 3.65 | 2.12 | 0.50 | 1.00 | 0.87 |
| gene-LOC5579195 | AAEL004272-PA; CUGBP Elav-like family member 4 isoform X1-X6 | 1.64 | 0.04 | 1.45 | 5.91 | 4.16 | 1.05 | 0.81 | 1.51 |
| gene-LOC5573436 | facilitated trehalose transporter Tret1 | 1.64 | 0.00 | 3.80 | 5.27 | 2.96 | 1.13 | 1.04 | 1.63 |
| gene-LOC5570911 | acylphosphatase-2 | 1.64 | 0.04 | 2.44 | 7.79 | 3.24 | 1.02 | 1.59 | 1.64 |
| gene-LOC11067777[illegible] | uncharacterized protein LOC110677773 | 1.64 | 0.01 | 3.71 | 7.67 | 2.63 | 1.54 | 1.27 | 1.62 |
| gene-LOC5568636 | AAEL006990-PA; putative phosphatidate phosphatase isoform X1; uncharacterized protein T28D9.3 isoform X2 | 1.63 | 0.00 | 10.42 | 11.01 | 13.02 | 6.31 | 2.86 | 0.77 |
| gene-LOC5577066 | AAEL003104-PA; RING finger protein nhl-1 isoform X1/X2 | 1.63 | 0.00 | 4.80 | 7.48 | 4.52 | 1.48 | 2.00 | 1.88 |
| gene-LOC5571839 | zwei Ig domain protein zig-8 | 1.62 | 0.03 | 17.42 | 13.89 | 11.65 | 2.90 | 7.25 | 3.54 |
| gene-LOC5567918 | sphingomyelin phosphodiesterase | 1.62 | 0.00 | 7.01 | 6.28 | 5.17 | 1.36 | 2.38 | 2.19 |
| gene-LOC5567092 | vitellogenin-1 | 1.61 | 0.00 | 10.00 | 13.84 | 7.62 | 3.93 | 3.29 | 2.84 |
| novel5361 | hypothetical protein RP20_CCG008133 | 1.61 | 0.01 | 2.68 | 3.62 | 2.81 | 0.45 | 1.54 | 0.95 |
| gene-LOC5566677 | nose resistant to fluoxetine protein 6 isoform X1/X2 | 1.61 | 0.00 | 14.71 | 13.18 | 14.39 | 5.18 | 4.89 | 4.35 |
| gene-LOC5571183 | cytochrome c oxidase subunit 6B1 | 1.61 | 0.00 | 33.81 | 52.27 | 33.59 | 8.73 | 15.30 | 14.30 |
| gene-LOC5565660 | AAEL004912-PA; uncharacterized protein LOC5565660 | 1.61 | 0.02 | 5.16 | 3.83 | 2.52 | 0.96 | 1.33 | 1.99 |
| gene-LOC5567333 | uncharacterized protein LOC5567333 | 1.60 | 0.00 | 0.78 | 1.14 | 0.64 | 0.27 | 0.30 | 0.26 |
| gene-LOC5565670 | sterile alpha and TIR motif-containing protein 1 isoform X1-X6 | 1.60 | 0.00 | 8.68 | 10.92 | 7.59 | 3.01 | 3.10 | 2.72 |
| gene-LOC5568890 | proton-coupled amino acid transporter-like protein pathetic isoform X1/X2 | 1.60 | 0.00 | 6.12 | 10.31 | 7.02 | 1.70 | 3.56 | 2.38 |

| | | | | | | | | | |
|---|---|---|---|---|---|---|---|---|---|
| gene-LOC11067426[illegible] | uncharacterized protein LOC110674263 | 1.59 | 0.00 | 4.31 | 6.69 | 2.69 | 1.34 | 1.71 | 1.43 |
| gene-LOC5564459 | sialin | 1.59 | 0.00 | 6.37 | 7.82 | 5.38 | 1.77 | 2.14 | 2.52 |
| gene-LOC5572220 | uncharacterized protein LOC5572220 | 1.58 | 0.04 | 6.00 | 8.31 | 4.61 | 0.81 | 3.05 | 2.35 |
| gene-LOC5566824 | PDZ and LIM domain protein Zasp isoform X6; AAEL013438-PA | 1.58 | 0.00 | 14.93 | 21.12 | 15.55 | 5.51 | 7.74 | 5.22 |
| gene-LOC5579856 | synaptic vesicle glycoprotein 2C | 1.57 | 0.00 | 48.02 | 109.38 | 53.59 | 19.43 | 20.13 | 28.57 |
| gene-LOC5575825 | aminopeptidase N isoform X1 | 1.57 | 0.00 | 1.13 | 2.55 | 2.27 | 0.56 | 0.90 | 0.61 |
| gene-LOC5566982 | AAEL005741-PA; forkhead box protein E3 isoform X1-X4 | 1.56 | 0.00 | 2.94 | 4.32 | 2.67 | 1.18 | 1.31 | 0.87 |
| gene-LOC11068157[illegible] | uncharacterized protein LOC110681571 | 1.56 | 0.00 | 1.77 | 2.22 | 1.59 | 0.89 | 0.64 | 0.94 |
| gene-LOC5569294 | uncharacterized protein LOC5569294 isoform X1-X4 | 1.55 | 0.01 | 0.92 | 1.35 | 0.91 | 0.32 | 0.42 | 0.30 |
| gene-LOC5576118 | uncharacterized protein KIAA1683 homolog | 1.55 | 0.05 | 3.75 | 5.37 | 1.63 | 0.73 | 1.15 | 1.74 |
| gene-LOC5569608 | uncharacterized protein LOC5569608 isoform X1/X2 | 1.55 | 0.01 | 5.80 | 9.99 | 7.30 | 2.08 | 1.90 | 3.83 |
| gene-LOC5576503 | myosin heavy chain, striated | 1.55 | 0.00 | 2.79 | 5.51 | 3.97 | 1.32 | 1.06 | 1.81 |
| gene-LOC5571480 | solute carrier organic anion transporter family member 2A1 | 1.55 | 0.01 | 1.25 | 2.82 | 1.67 | 0.58 | 0.75 | 0.60 |
| gene-LOC5574136 | alpha-tocopherol transfer protein-like | 1.55 | 0.01 | 3.84 | 4.74 | 2.90 | 1.48 | 1.59 | 0.91 |
| novel2317 | uncharacterized protein Dwil_GK26988, partial | 1.55 | 0.04 | 1.34 | 1.57 | 1.16 | 0.47 | 0.38 | 0.53 |
| gene-LOC5574092 | uncharacterized protein LOC5574092 | 1.54 | 0.00 | 8.82 | 15.19 | 11.18 | 2.43 | 5.07 | 4.38 |
| novel2253 | - | 1.54 | 0.00 | 19.51 | 13.04 | 14.37 | 5.70 | 4.49 | 6.14 |
| gene-LOC11067561[illegible] | uncharacterized protein LOC110675610 | 1.54 | 0.00 | 60.23 | 114.38 | 90.77 | 29.99 | 32.99 | 25.62 |
| gene-LOC5574604 | troponin T, skeletal muscle-like | 1.54 | 0.00 | 14.27 | 31.37 | 29.15 | 8.61 | 7.78 | 8.09 |
| gene-LOC5579926 | cytochrome P450 9e2 | 1.54 | 0.00 | 3.72 | 6.39 | 4.56 | 1.21 | 2.10 | 1.66 |
| gene-LOC23687721 | elongation factor 1-alpha | 1.53 | 0.00 | 22.71 | 35.30 | 23.55 | 8.31 | 12.82 | 6.65 |
| gene-LOC5571788 | angiotensin-converting enzyme | 1.53 | 0.00 | 3.18 | 4.30 | 4.78 | 0.93 | 1.30 | 1.97 |

| | | | | | | | | | |
|---|---|---|---|---|---|---|---|---|---|
| gene-LOC5570135 | keratin, type I cytoskeletal 9 | 1.52 | 0.02 | 5.25 | 5.53 | 3.00 | 1.95 | 1.34 | 1.36 |
| gene-LOC5575534 | uncharacterized protein LOC5575534 | 1.52 | 0.02 | 0.31 | 2.30 | 0.82 | 0.17 | 0.16 | 0.21 |
| gene-LOC5574015 | AAEL010850-PB/PD/PE/PG; troponin I isoform X1-X6 | 1.52 | 0.00 | 41.67 | 87.99 | 65.72 | 22.93 | 24.72 | 20.44 |
| gene-LOC5575342 | general odorant-binding protein | 1.52 | 0.00 | 14.21 | 20.93 | 15.58 | 6.86 | 5.75 | 4.44 |
| gene-LOC5566333 | LOW QUALITY PROTEIN: titin | 1.52 | 0.00 | 0.89 | 0.65 | 0.43 | 0.24 | 0.23 | 0.21 |
| gene-LOC5565084 | uncharacterized protein LOC5565084 | 1.52 | 0.00 | 4.87 | 8.11 | 6.45 | 2.54 | 1.70 | 2.47 |
| gene-LOC5563689 | pacifastin-like protease inhibitor cvp4 | 1.51 | 0.00 | 9.28 | 22.11 | 11.44 | 2.64 | 5.41 | 6.82 |
| gene-LOC5570781 | endothelin-converting enzyme 2 | 1.51 | 0.01 | 1.65 | 1.91 | 1.10 | 0.65 | 0.50 | 0.49 |
| gene-LOC5578104 | regulator of microtubule dynamics protein 1 | 1.51 | 0.01 | 4.28 | 7.20 | 6.10 | 1.02 | 2.59 | 2.50 |
| gene-LOC5577717 | protein lethal(2)essential for life | 1.51 | 0.00 | 13.47 | 30.20 | 16.80 | 4.92 | 9.51 | 6.35 |
| gene-LOC5571127 | uncharacterized protein LOC5571127 isoform X1-X3 | 1.51 | 0.00 | 4.32 | 8.67 | 8.39 | 1.87 | 2.98 | 2.50 |
| gene-LOC11067403[illegible] | centrosomal and chromosomal factor-like | 1.51 | 0.00 | 1.31 | 1.70 | 0.96 | 0.43 | 0.37 | 0.58 |
| gene-LOC5577270 | guanine nucleotide-binding protein G(f) subunit alpha | 1.50 | 0.00 | 3.80 | 6.16 | 5.40 | 1.97 | 2.25 | 1.37 |
| gene-LOC5576131 | L-asparaginase | 1.50 | 0.02 | 1.41 | 2.65 | 1.42 | 0.60 | 0.79 | 0.55 |
| gene-LOC5569780 | retinoid-inducible serine carboxypeptidase | 1.50 | 0.00 | 21.30 | 20.03 | 17.38 | 4.15 | 7.13 | 9.10 |
| gene-LOC5566301 | galectin-4 | 1.50 | 0.00 | 16.85 | 30.34 | 15.28 | 5.96 | 6.31 | 9.42 |
| novel5382 | unnamed protein product, partial | 1.50 | 0.03 | 0.43 | 0.48 | 0.26 | 0.10 | 0.12 | 0.15 |
| gene-LOC5574516 | phosphate carrier protein, mitochondrial | 1.50 | 0.00 | 14.71 | 25.32 | 18.90 | 6.76 | 5.78 | 7.96 |
| gene-LOC5573776 | gamma-glutamyl hydrolase isoform X1-X3 | 1.49 | 0.00 | 10.10 | 13.17 | 7.71 | 3.33 | 2.98 | 4.72 |
| gene-LOC5576180 | general odorant-binding protein 72 | 1.49 | 0.00 | 18.16 | 18.92 | 15.30 | 7.14 | 4.73 | 6.03 |
| gene-LOC5563800 | uncharacterized protein LOC5563800 | 1.49 | 0.00 | 1339.33 | 1789.48 | 920.61 | 361.85 | 551.52 | 497.31 |
| gene-LOC5575085 | transferrin | 1.49 | 0.00 | 13.17 | 16.78 | 13.70 | 5.16 | 4.92 | 5.22 |
| gene-LOC11068119[illegible] | solute carrier family 46 member 3 | 1.49 | 0.00 | 4.63 | 5.28 | 2.66 | 1.34 | 1.96 | 1.64 |

| | | | | | | | | | |
|---|---|---|---|---|---|---|---|---|---|
| gene-LOC5566573 | ABC transporter G family member 20 isoform X1-X3 | 1.49 | 0.02 | 1.39 | 2.19 | 1.04 | 0.46 | 0.52 | 0.71 |
| gene-LOC5578692 | serine protease easter | 1.49 | 0.00 | 9.40 | 18.26 | 8.30 | 2.98 | 4.95 | 4.70 |
| gene-LOC5565841 | multidrug resistance-associated protein 1 isoform X1/X2 | 1.48 | 0.00 | 2.66 | 2.72 | 2.42 | 0.53 | 0.90 | 1.34 |
| gene-LOC5571433 | LOW QUALITY PROTEIN: cholinesterase | 1.48 | 0.00 | 1.34 | 1.76 | 1.58 | 0.48 | 0.80 | 0.37 |
| gene-LOC5572129 | transient receptor potential cation channel protein painless isoform | 1.48 | 0.01 | 1.00 | 1.46 | 1.09 | 0.36 | 0.54 | 0.36 |
| gene-LOC5577084 | solute carrier family 22 member 8 | 1.48 | 0.00 | 3.91 | 6.85 | 4.86 | 0.91 | 2.36 | 2.27 |
| gene-LOC5580019 | uncharacterized protein LOC5580019 | 1.48 | 0.00 | 26.16 | 43.98 | 35.61 | 11.36 | 12.30 | 13.74 |
| gene-LOC5576452 | protein javelin isoform X1/X2; AAEL012536-PA | 1.47 | 0.00 | 3.40 | 5.20 | 2.81 | 0.88 | 1.41 | 1.39 |
| gene-LOC5576787 | uncharacterized protein LOC5576787 | 1.47 | 0.04 | 1.70 | 2.39 | 2.04 | 1.03 | 0.47 | 0.66 |
| gene-LOC5577771 | peptide transporter family 1 | 1.47 | 0.00 | 5.37 | 5.37 | 4.55 | 1.24 | 2.01 | 2.12 |
| gene-LOC5566760 | uncharacterized protein LOC5566760 | 1.47 | 0.00 | 4.35 | 4.79 | 3.66 | 0.79 | 2.02 | 1.78 |
| gene-LOC5571786 | solute carrier family 46 member 3 | 1.46 | 0.03 | 1.51 | 1.58 | 1.13 | 0.48 | 0.45 | 0.57 |
| gene-LOC5566457 | probable salivary secreted peptide | 1.46 | 0.00 | 91.74 | 81.49 | 109.57 | 31.09 | 30.23 | 37.97 |
| gene-LOC5568365 | AAEL001091-PA; NADP-dependent malic enzyme | 1.45 | 0.00 | 5.47 | 8.02 | 4.65 | 2.09 | 2.10 | 2.41 |
| gene-LOC5566352 | neurexin-4 isoform X1-X4 | 1.45 | 0.00 | 1.89 | 2.35 | 2.05 | 0.52 | 0.80 | 0.96 |
| gene-LOC5575671 | xanthine dehydrogenase | 1.45 | 0.00 | 2.02 | 3.68 | 2.30 | 0.93 | 1.09 | 0.86 |
| gene-LOC5569807 | AAEL001287-PB; uncharacterized protein LOC5569807 | 1.45 | 0.00 | 8.92 | 15.35 | 6.96 | 2.15 | 2.63 | 4.29 |
| gene-LOC5565804 | aquaporin | 1.45 | 0.00 | 20.80 | 38.56 | 19.85 | 7.26 | 9.48 | 11.72 |
| gene-LOC5563672 | AAEL000563-PA; C-type lectin | 1.45 | 0.00 | 3.12 | 3.25 | 2.63 | 1.01 | 1.39 | 1.05 |
| gene-LOC5579417 | transferrin | 1.45 | 0.00 | 16.67 | 11.72 | 6.30 | 3.86 | 3.61 | 5.11 |
| gene-LOC5572108 | uncharacterized protein LOC5572108 isoform X1-X4 | 1.45 | 0.02 | 2.49 | 4.35 | 1.89 | 0.92 | 1.59 | 0.73 |
| gene-LOC11067958[illegible] | caldesmon-like | 1.45 | 0.00 | 5.32 | 8.64 | 6.50 | 2.62 | 2.99 | 2.28 |
| gene-LOC5569794 | patched domain-containing protein | 1.44 | 0.03 | 0.58 | 0.47 | 0.38 | 0.19 | 0.19 | 0.14 |
| gene-LOC5576416 | venom carboxylesterase-6 | 1.44 | 0.04 | 1.40 | 3.89 | 2.47 | 0.63 | 1.31 | 0.87 |

| | | | | | | | | | |
|---|---|---|---|---|---|---|---|---|---|
| novel306 | - | 1.44 | 0.02 | 0.44 | 0.67 | 0.28 | 0.25 | 0.21 | 0.10 |
| gene-LOC5570111 | trypsin alpha-3 | 1.44 | 0.00 | 9.69 | 6.47 | 9.25 | 2.18 | 3.65 | 3.34 |
| gene-LOC5579061 | guanine nucleotide-binding protein subunit beta-1 | 1.43 | 0.02 | 0.91 | 1.17 | 0.77 | 0.24 | 0.56 | 0.25 |
| gene-LOC5572286 | uncharacterized protein LOC5572286 | 1.43 | 0.04 | 4.50 | 6.32 | 6.18 | 2.22 | 2.55 | 1.32 |
| gene-LOC5574458 | uncharacterized protein LOC5574458 | 1.43 | 0.00 | 74.57 | 117.36 | 83.17 | 27.95 | 34.57 | 37.42 |
| gene-LOC5571828 | chymotrypsin-2 | 1.43 | 0.03 | 11.17 | 17.92 | 22.76 | 3.17 | 3.98 | 11.81 |
| gene-LOC5570163 | long-chain fatty acid transport protein 4 | 1.43 | 0.00 | 4.83 | 8.61 | 5.72 | 1.44 | 3.21 | 2.40 |
| gene-LOC5571779 | lachesin | 1.43 | 0.00 | 3.70 | 5.79 | 3.04 | 1.69 | 1.38 | 1.52 |
| gene-LOC5573047 | facilitated trehalose transporter Tret1 | 1.42 | 0.00 | 4.93 | 9.92 | 3.77 | 2.15 | 2.66 | 2.06 |
| gene-LOC5572507 | trypsin-1 | 1.42 | 0.03 | 3.55 | 9.71 | 6.76 | 2.03 | 3.14 | 2.16 |
| gene-LOC5566684 | nucleosome assembly protein 1-like 4 | 1.42 | 0.00 | 12.48 | 27.47 | 17.04 | 7.14 | 8.05 | 6.32 |
| gene-LOC5576995 | chitinase-3-like protein 1 | 1.42 | 0.00 | 25.19 | 33.29 | 25.38 | 9.58 | 9.66 | 11.90 |
| gene-LOC11067413 | dynein heavy chain 10, axonemal | 1.41 | 0.01 | 15.19 | 27.66 | 15.54 | 0.38 | 0.39 | 4.01 |
| gene-LOC5576800 | aminopeptidase N | 1.41 | 0.00 | 7.04 | 12.61 | 7.39 | 2.14 | 2.74 | 4.94 |
| gene-LOC11068129 | uncharacterized protein LOC110681295 | 1.41 | 0.01 | 2.70 | 2.97 | 2.35 | 0.99 | 1.34 | 0.49 |
| gene-LOC5564903 | AAEL014650-PA; serine proteinase stubble isoform X3; mucin-5AC isoform X1/X2 | 1.41 | 0.00 | 4.94 | 5.73 | 3.45 | 2.41 | 2.31 | 1.20 |
| gene-LOC5573437 | facilitated trehalose transporter Tret1 | 1.40 | 0.00 | 24.38 | 28.86 | 26.74 | 8.24 | 9.47 | 12.13 |
| gene-LOC5579827 | AAEL005518-PA; uncharacterized protein LOC110681442; uncharacterized protein LOC5579827 | 1.40 | 0.01 | 6.68 | 7.76 | 11.63 | 7.03 | 2.87 | 3.66 |
| gene-LOC5568118 | uncharacterized protein LOC5568118 | 1.40 | 0.00 | 2.93 | 3.33 | 2.54 | 0.78 | 1.62 | 1.20 |
| gene-LOC5580119 | uncharacterized protein LOC5580119 isoform X1/X3; | 1.39 | 0.00 | 7.66 | 16.25 | 11.44 | 3.62 | 3.68 | 6.52 |

| | | | | | | | | | |
|---|---|---|---|---|---|---|---|---|---|
| gene-LOC5572936 | probable cytochrome P450 6a14 | 1.39 | 0.03 | 2.30 | 4.32 | 2.03 | 1.15 | 0.95 | 1.15 |
| gene-LOC5567410 | shootin-1 | 1.39 | 0.00 | 1.92 | 3.51 | 2.90 | 1.13 | 0.96 | 1.17 |
| gene-LOC5563829 | zinc transporter ZIP14 | 1.38 | 0.01 | 2.66 | 3.12 | 1.92 | 0.73 | 0.66 | 1.55 |
| gene-LOC5573074 | venom allergen 5 isoform X1/X2 | 1.38 | 0.01 | 5.22 | 10.18 | 4.50 | 2.32 | 2.76 | 2.45 |
| gene-LOC5569731 | spondin-1 | 1.37 | 0.00 | 7.58 | 10.52 | 8.32 | 2.94 | 4.08 | 3.08 |
| gene-LOC5575014 | AAEL011565-PA; hypothetical protein RP20_CCG009379; ankyrin-2 isoform X4/X5/X10; | 1.37 | 0.04 | 1.39 | 3.02 | 1.61 | 0.44 | 1.06 | 0.43 |
| gene-LOC5568002 | dual specificity protein phosphatase 3 isoform X3-X5; hypothetical protein | 1.37 | 0.01 | 3.01 | 5.68 | 3.85 | 2.05 | 1.73 | 1.06 |
| gene-LOC5570210 | aminopeptidase N isoform X1/X2 | 1.37 | 0.01 | 1.57 | 2.05 | 1.81 | 0.51 | 0.76 | 0.81 |
| gene-LOC5563757 | uncharacterized protein LOC5563757 | 1.37 | 0.01 | 1.74 | 2.28 | 1.11 | 0.68 | 0.66 | 0.63 |
| gene-LOC5566047 | DNA-binding protein D-ETS-4 isoform X1-X3 | 1.37 | 0.00 | 1.86 | 1.94 | 1.55 | 0.56 | 0.82 | 0.71 |
| gene-LOC5571997 | uncharacterized protein LOC5571997 | 1.37 | 0.00 | 27.93 | 49.19 | 40.87 | 14.47 | 15.78 | 14.71 |
| gene-LOC5568444 | AAEL006866-PA; muscle LIM protein 1 isoform X2; muscle LIM protein Mlp84B isoform X1 | 1.36 | 0.00 | 72.13 | 138.85 | 87.69 | 39.83 | 47.19 | 35.43 |
| novel2418 | - | 1.36 | 0.04 | 0.47 | 0.70 | 0.32 | 0.19 | 0.24 | 0.15 |
| gene-LOC5571215 | proton-coupled amino acid transporter-like protein CG1139 | 1.36 | 0.00 | 4.40 | 3.94 | 3.40 | 1.48 | 1.77 | 1.69 |
| gene-LOC5573594 | AAEL002127-PA; UPF0769 protein CG18675 | 1.36 | 0.04 | 0.66 | 2.00 | 1.73 | 0.17 | 0.25 | 0.35 |
| gene-LOC5573632 | uncharacterized protein LOC5573632 | 1.36 | 0.00 | 12.87 | 12.99 | 15.31 | 3.97 | 5.79 | 5.76 |
| gene-LOC5569224 | AAEL007493-PA; uncharacterized protein LOC5569224 | 1.35 | 0.01 | 7.85 | 8.07 | 7.13 | 2.46 | 3.21 | 3.07 |
| gene-LOC23687481 | AAEL017061-PA | 1.35 | 0.01 | 3.69 | 7.71 | 5.11 | 1.31 | 2.84 | 2.23 |
| gene-LOC11067929( | protein PRRC2B-like | 1.35 | 0.01 | 8.59 | 7.69 | 5.61 | 4.24 | 1.90 | 4.41 |
| gene-LOC5570863 | ATP-binding cassette sub-family G member 4 | 1.35 | 0.05 | 1.28 | 1.93 | 0.94 | 0.34 | 0.46 | 0.82 |
| gene-LOC5577490 | protein aubergine | 1.35 | 0.00 | 2.26 | 3.77 | 3.19 | 1.16 | 1.51 | 0.92 |

| | | | | | | | | | |
|---|---|---|---|---|---|---|---|---|---|
| novel339 | unnamed protein product | 1.34 | 0.01 | 0.73 | 1.26 | 0.60 | 0.39 | 0.33 | 0.28 |
| gene-LOC5578738 | glia-derived nexin | 1.34 | 0.00 | 4.06 | 7.18 | 4.43 | 3.84 | 2.35 | 5.63 |
| gene-LOC5578114 | L-threonine 3-dehydrogenase, mitochondrial | 1.34 | 0.00 | 8.11 | 20.84 | 11.40 | 4.99 | 10.17 | 5.02 |
| gene-LOC5565181 | actin | 1.33 | 0.04 | 4.36 | 3.20 | 2.82 | 0.93 | 1.63 | 1.49 |
| gene-LOC5567606 | uncharacterized protein LOC5567606 | 1.33 | 0.05 | 15.30 | 11.21 | 9.92 | 1.13 | 8.33 | 4.92 |
| gene-LOC5576419 | calcium-binding protein E63-1 isoform X1-X7; AAEL012513-PA | 1.33 | 0.00 | 6.23 | 7.62 | 5.10 | 3.33 | 2.61 | 2.39 |
| gene-LOC5566991 | flocculation protein FLO11 isoform X1/X2 | 1.33 | 0.01 | 1.33 | 2.03 | 1.45 | 0.57 | 0.92 | 0.48 |
| gene-LOC5570852 | ATP-binding cassette sub-family G member 1 | 1.33 | 0.00 | 2.51 | 3.67 | 3.25 | 1.14 | 1.30 | 1.26 |
| gene-LOC5571799 | AAEL009323-PA; putative carbonic anhydrase 3 | 1.33 | 0.00 | 8.44 | 11.16 | 7.38 | 2.67 | 3.44 | 4.40 |
| gene-LOC5574012 | serine-rich adhesin for platelets isoform X1; ubiquitin carboxyl-terminal hydrolase 2 isoform X2-X4 | 1.33 | 0.00 | 1.56 | 2.48 | 1.52 | 8.28 | 0.49 | 0.78 |
| gene-LOC5573855 | zinc carboxypeptidase A 1 | 1.33 | 0.00 | 19.27 | 41.87 | 32.32 | 9.43 | 13.08 | 14.11 |
| gene-LOC5577555 | uncharacterized protein LOC5577555 | 1.32 | 0.02 | 2.58 | 5.34 | 5.50 | 1.30 | 1.50 | 2.48 |
| gene-LOC5571136 | carbonic anhydrase 2 | 1.32 | 0.00 | 3.60 | 5.15 | 5.40 | 1.57 | 1.52 | 2.46 |
| gene-LOC5570819 | CD109 antigen isoform X1-X12 | 1.32 | 0.00 | 7.75 | 11.58 | 6.40 | 3.71 | 3.52 | 3.18 |
| gene-LOC5563772 | proline-rich extensin-like protein EPR1 isoform X4/X5; nucleoporin nup124 isoform X1/X2 | 1.32 | 0.00 | 3.07 | 3.93 | 2.79 | 1.11 | 1.39 | 1.44 |
| gene-LOC5576364 | uncharacterized protein LOC5576364 | 1.32 | 0.03 | 2.25 | 2.55 | 1.36 | 0.68 | 1.19 | 0.57 |
| gene-LOC5566917 | receptor-type tyrosine-protein phosphatase-like N isoform X1-X3 | 1.31 | 0.00 | 7.90 | 10.56 | 6.68 | 3.54 | 4.41 | 2.32 |
| gene-LOC5569932 | uncharacterized protein LOC5569932 | 1.30 | 0.00 | 74.14 | 62.90 | 9.63 | 21.55 | 7.14 | 34.02 |

| | | | | | | | | | |
|---|---|---|---|---|---|---|---|---|---|
| gene-LOC5566151 | hypothetical protein RP20_CCG024675; PDZ and LIM domain protein 7 isoform X1/X3/X4; bromodomain-containing protein 4 isoform X2 | 1.30 | 0.00 | 15.64 | 26.53 | 19.44 | 10.93 | 11.39 | 5.16 |
| gene-LOC5570108 | 40S ribosomal protein SA | 1.30 | 0.00 | 953.06 | 1310.78 | 1186.24 | 357.17 | 353.48 | 624.45 |
| gene-LOC5567097 | uridine phosphorylase 1 isoform X1/X2 | 1.30 | 0.00 | 5.56 | 6.45 | 3.32 | 1.79 | 2.39 | 1.92 |
| gene-LOC5574703 | uncharacterized protein LOC5574703 isoform X1/X2 | 1.30 | 0.00 | 5.38 | 6.78 | 3.85 | 1.56 | 2.90 | 1.86 |
| gene-LOC5571787 | angiotensin-converting enzyme | 1.30 | 0.00 | 3.93 | 3.90 | 3.48 | 1.47 | 1.76 | 2.73 |
| gene-LOC5566650 | multidrug resistance-associated protein 9 | 1.30 | 0.05 | 1.08 | 1.15 | 0.64 | 0.34 | 0.53 | 0.26 |
| gene-LOC5574204 | leucine-rich repeat neuronal protein 3 | 1.30 | 0.00 | 14.90 | 19.12 | 17.44 | 6.27 | 7.05 | 6.94 |
| gene-LOC5569644 | uncharacterized protein LOC5569644 | 1.30 | 0.01 | 3.23 | 3.57 | 3.52 | 0.84 | 1.70 | 1.49 |
| gene-LOC5568893 | AAEL007208-PA; protein anachronism | 1.30 | 0.01 | 2.24 | 5.23 | 3.96 | 1.01 | 2.33 | 1.27 |
| gene-LOC5573421 | protein cycle isoform X3/X4; aryl hydrocarbon receptor nuclear translocator-like protein 1 isoform X1/X2; uncharacterized protein LOC26530279, partial | 1.30 | 0.00 | 6.08 | 7.95 | 6.32 | 4.56 | 4.15 | 3.86 |
| gene-LOC5575209 | acyl-CoA synthetase family member 2, mitochondrial isoform | 1.30 | 0.02 | 1.83 | 3.95 | 2.60 | 1.02 | 1.03 | 1.32 |
| gene-LOC5568779 | facilitated trehalose transporter Tret1-2 homolog isoform X1/X2 | 1.29 | 0.01 | 3.55 | 4.53 | 3.68 | 1.11 | 1.45 | 2.16 |
| gene-LOC5571544 | probable cytochrome P450 6d4 | 1.29 | 0.00 | 19.63 | 23.47 | 20.46 | 6.17 | 8.96 | 10.53 |
| gene-LOC5569681 | trypsin 3A1 isoform X1/X2 | 1.29 | 0.00 | 11138.95 | 15106.60 | 13651.55 | 3925.01 | 4864.91 | 7215.26 |
| gene-LOC5578054 | retinol dehydrogenase 13 | 1.29 | 0.04 | 3.53 | 5.03 | 2.92 | 2.02 | 1.03 | 1.57 |
| gene-LOC5576518 | probable phosphoserine aminotransferase | 1.28 | 0.04 | 3.51 | 11.77 | 6.13 | 2.24 | 2.42 | 4.01 |
| gene-LOC5576676 | ATP-binding cassette sub-family A member 3 | 1.28 | 0.00 | 1.92 | 2.01 | 1.81 | 0.73 | 0.86 | 0.74 |

| | | | | | | | | | |
|---|---|---|---|---|---|---|---|---|---|
| gene-LOC11067686[illegible] | collagen alpha-1(IV) chain | 1.28 | 0.00 | 1.97 | 3.08 | 1.62 | 1.00 | 0.90 | 0.81 |
| gene-LOC5566780 | membrane alanyl aminopeptidase | 1.28 | 0.00 | 5.39 | 7.30 | 5.35 | 2.02 | 3.16 | 2.25 |
| gene-LOC5569145 | basic-leucine zipper transcription factor A isoform X5; putative mediator of RNA polymerase II transcription subunit 26 isoform X4; trithorax group protein osa isoform X6; uncharacterized protein LOC5569145; AAEL007407-PB; AGAP005807-PC; AAEL007408-PA; AGAP005807-PD | 1.28 | 0.00 | 18.97 | 34.80 | 23.88 | 10.97 | 12.67 | 9.50 |
| gene-LOC5572019 | protein stoned-B | 1.27 | 0.00 | 2.14 | 1.94 | 1.27 | 0.68 | 0.85 | 0.67 |
| gene-LOC5575133 | uncharacterized protein LOC5575133 | 1.27 | 0.01 | 4.45 | 5.89 | 4.89 | 1.26 | 1.41 | 2.83 |
| gene-LOC5572950 | WD repeat-containing protein 87 isoform X1/X2; DNA ligase 1 isoform X3 | 1.27 | 0.02 | 1.36 | 2.79 | 2.28 | 0.78 | 1.09 | 0.77 |
| gene-LOC5573760 | mini-chromosome maintenance complex-binding protein; lysozyme | 1.27 | 0.02 | 6.91 | 9.32 | 6.94 | 3.07 | 3.60 | 1.54 |
| gene-LOC5579332 | general odorant-binding protein 67 | 1.27 | 0.04 | 8.22 | 11.59 | 6.38 | 2.83 | 3.32 | 4.47 |
| gene-LOC5566109 | uncharacterized protein LOC5566109 | 1.27 | 0.00 | 484.61 | 691.28 | 332.13 | 172.96 | 254.36 | 173.04 |
| gene-LOC5579975 | peptidyl-alpha-hydroxyglycine alpha-amidating lyase 2 | 1.27 | 0.00 | 8.70 | 12.86 | 9.27 | 4.38 | 4.58 | 3.66 |
| gene-LOC11067415[illegible] | heat shock protein 70 A1 | 1.26 | 0.01 | 5.86 | 17.10 | 16.45 | 3.63 | 7.88 | 4.50 |
| gene-LOC5572744 | uncharacterized protein LOC5572744 | 1.26 | 0.00 | 3.28 | 2.34 | 2.99 | 0.83 | 1.53 | 1.21 |
| gene-LOC5566335 | titin; uncharacterized protein LOC109419679 | 1.26 | 0.00 | 3.73 | 6.85 | 4.53 | 1.82 | 2.20 | 1.68 |
| novel2629 | AAEL009670-PA | 1.26 | 0.02 | 51.74 | 97.66 | 84.38 | 43.70 | 34.44 | 14.32 |
| gene-LOC5568576 | serine-enriched protein isoform X1/X2 | 1.26 | 0.04 | 0.62 | 0.62 | 0.59 | 0.24 | 0.28 | 0.23 |
| novel114 | - | 1.26 | 0.03 | 0.96 | 1.05 | 0.58 | 0.31 | 0.33 | 0.43 |
| gene-LOC5578380 | SPARC | 1.26 | 0.01 | 7.51 | 14.67 | 8.75 | 2.45 | 4.82 | 5.50 |

| | | | | | | | | | |
|---|---|---|---|---|---|---|---|---|---|
| gene-LOC5573256 | uncharacterized protein LOC5573256 | 1.25 | 0.01 | 10.37 | 21.00 | 12.96 | 4.85 | 7.00 | 6.35 |
| gene-LOC5577601 | larval cuticle protein LCP-30 | 1.25 | 0.03 | 12.19 | 29.36 | 9.50 | 6.47 | 8.73 | 5.78 |
| gene-LOC5576203 | uncharacterized protein LOC5576203 | 1.25 | 0.01 | 6.00 | 6.83 | 6.96 | 1.40 | 2.95 | 3.85 |
| gene-LOC5576327 | proteoglycan 4 | 1.25 | 0.01 | 3.42 | 4.27 | 3.59 | 1.04 | 2.19 | 1.41 |
| novel2199 | AAEL005098-PA | 1.25 | 0.00 | 14.70 | 16.00 | 15.72 | 6.56 | 8.37 | 4.07 |
| gene-LOC5578455 | uncharacterized protein LOC5578455 | 1.25 | 0.03 | 9.48 | 18.61 | 13.09 | 2.91 | 5.30 | 8.87 |
| gene-LOC5578107 | RNA-binding protein 24-A | 1.24 | 0.00 | 1.54 | 1.91 | 1.44 | 0.80 | 0.78 | 0.53 |
| gene-LOC5572372 | mite group 2 allergen Lep d 2 | 1.24 | 0.00 | 160.90 | 211.56 | 166.48 | 63.44 | 63.66 | 95.48 |
| gene-LOC5575522 | 1-acyl-sn-glycerol-3-phosphate acyltransferase alpha | 1.24 | 0.01 | 4.02 | 2.78 | 2.35 | 1.26 | 1.61 | 1.39 |
| gene-LOC5572546 | low-density lipoprotein receptor-related protein 2 | 1.24 | 0.00 | 2.22 | 2.34 | 1.30 | 0.71 | 0.77 | 0.98 |
| gene-LOC11067423 | uncharacterized protein LOC110674237 | 1.24 | 0.00 | 392.88 | 888.21 | 577.99 | 198.43 | 232.80 | 338.80 |
| gene-LOC5578089 | maltase A3 | 1.24 | 0.00 | 8.54 | 14.26 | 10.92 | 3.43 | 5.64 | 5.01 |
| gene-LOC5564567 | seminal metalloprotease 1; AAEL014516-PA | 1.24 | 0.01 | 507.93 | 585.50 | 609.70 | 163.65 | 186.33 | 355.48 |
| gene-LOC5566344 | carbonic anhydrase 1 | 1.23 | 0.00 | 9.00 | 8.18 | 6.67 | 3.01 | 4.34 | 3.08 |
| gene-LOC5579807 | uncharacterized protein LOC5579807 | 1.23 | 0.00 | 15.16 | 15.87 | 13.09 | 5.38 | 8.92 | 4.09 |
| gene-LOC5576849 | cytochrome b561 | 1.23 | 0.01 | 5.45 | 7.96 | 6.66 | 2.28 | 3.33 | 3.60 |
| gene-LOC5564099 | uncharacterized protein LOC5564099 | 1.23 | 0.00 | 3.26 | 5.04 | 3.02 | 1.07 | 1.62 | 1.82 |
| gene-LOC5580168 | twitchin | 1.23 | 0.00 | 4.46 | 5.59 | 3.74 | 1.90 | 2.02 | 1.89 |
| gene-LOC5572634 | carboxypeptidase B | 1.23 | 0.00 | 811.91 | 1121.87 | 1118.36 | 291.30 | 380.46 | 609.68 |
| gene-LOC5563773 | uncharacterized protein LOC5563773 | 1.23 | 0.02 | 8.19 | 14.12 | 11.54 | 2.98 | 6.38 | 4.78 |
| gene-LOC5564404 | uncharacterized protein LOC5564404 | 1.23 | 0.00 | 11.32 | 12.08 | 8.74 | 2.68 | 4.41 | 6.68 |
| gene-LOC5564135 | hemicentin-1; uncharacterized protein LOC5564135 | 1.23 | 0.00 | 3.33 | 4.64 | 2.57 | 2.17 | 1.83 | 1.56 |

| | | | | | | | | | |
|---|---|---|---|---|---|---|---|---|---|
| gene-LOC5571113 | AAEL001509-PB; uncharacterized protein LOC5571113 | 1.23 | 0.00 | 3.88 | 5.59 | 3.49 | 2.07 | 1.83 | 1.53 |
| gene-LOC11067686[illegible] | hypothetical protein RP20_CCG020089 | 1.22 | 0.00 | 3.35 | 4.35 | 2.55 | 1.38 | 1.62 | 1.32 |
| gene-LOC5578143 | uncharacterized protein LOC5578143 | 1.22 | 0.01 | 6.24 | 6.13 | 3.21 | 2.30 | 2.83 | 1.83 |
| gene-LOC5567620 | glycogen-binding subunit 76A | 1.22 | 0.02 | 1.46 | 1.89 | 1.14 | 0.65 | 0.76 | 0.49 |
| gene-LOC5573214 | homeobox protein six1b | 1.22 | 0.04 | 2.66 | 6.23 | 4.17 | 1.63 | 1.52 | 2.36 |
| gene-LOC5574161 | paramyosin | 1.22 | 0.00 | 26.20 | 43.21 | 27.97 | 13.18 | 17.88 | 14.37 |
| gene-LOC5570825 | zinc carboxypeptidase | 1.21 | 0.00 | 7.68 | 10.26 | 7.43 | 2.86 | 3.67 | 4.07 |
| gene-LOC5567053 | general odorant-binding protein | 1.21 | 0.03 | 12.74 | 31.71 | 26.67 | 6.00 | 9.92 | 14.09 |
| gene-LOC11067967[illegible] | myosin heavy chain, clone 203-like | 1.21 | 0.00 | 9.58 | 15.62 | 9.08 | 5.98 | 5.05 | 3.54 |
| gene-LOC5567084 | membrane alanyl aminopeptidase | 1.21 | 0.00 | 6.04 | 5.15 | 4.31 | 1.76 | 2.55 | 2.32 |
| gene-LOC5578882 | uncharacterized protein LOC5578882 | 1.20 | 0.00 | 994.18 | 1018.45 | 867.60 | 288.82 | 423.08 | 497.57 |
| novel93 | uncharacterized protein LOC109405315 | 1.20 | 0.02 | 1.05 | 1.27 | 1.01 | 0.31 | 0.74 | 0.38 |
| gene-LOC11067398[illegible] | uncharacterized protein LOC110673985 | 1.20 | 0.04 | 5.09 | 3.20 | 3.08 | 1.41 | 0.90 | 2.50 |
| gene-LOC5575305 | protein snakeskin | 1.20 | 0.00 | 28.15 | 39.18 | 31.95 | 11.59 | 15.40 | 15.64 |
| gene-LOC5566701 | uncharacterized protein LOC5566701 | 1.20 | 0.00 | 37.46 | 61.99 | 30.01 | 17.80 | 27.36 | 14.76 |
| gene-LOC5570211 | aminopeptidase N | 1.20 | 0.00 | 9.19 | 10.95 | 9.43 | 3.47 | 4.11 | 5.15 |
| gene-LOC5566977 | chaoptin | 1.20 | 0.01 | 2.71 | 4.11 | 3.55 | 1.13 | 1.54 | 1.79 |
| gene-LOC5565699 | beta-hexosaminidase subunit beta | 1.19 | 0.01 | 3.21 | 4.11 | 3.13 | 1.24 | 1.48 | 1.89 |
| gene-LOC5578105 | regulator of microtubule dynamics protein 1 | 1.19 | 0.04 | 6.72 | 4.94 | 5.71 | 1.57 | 2.35 | 3.55 |
| gene-LOC5572559 | endothelin-converting enzyme homolog | 1.19 | 0.00 | 5.94 | 8.43 | 7.43 | 2.73 | 3.46 | 3.03 |
| gene-LOC5574368 | dopamine N-acetyltransferase | 1.19 | 0.00 | 8.03 | 11.23 | 7.93 | 4.38 | 4.87 | 3.38 |
| gene-LOC5576979 | trichohyalin; uncharacterized protein LOC5576979 | 1.19 | 0.00 | 4.64 | 8.16 | 6.06 | 2.71 | 3.40 | 2.41 |
| gene-LOC11067937[illegible] | uncharacterized protein LOC110679374 | 1.19 | 0.00 | 13.46 | 25.82 | 20.82 | 8.67 | 11.31 | 5.99 |
| gene-LOC5573469 | transcription factor cwo isoform X1 | 1.18 | 0.00 | 4.51 | 5.37 | 3.88 | 1.63 | 2.28 | 2.10 |

| | | | | | | | | | |
|---|---|---|---|---|---|---|---|---|---|
| gene-LOC5569143 | verprolin isoform X1; proteoglycan 4 isoform X2 | 1.18 | 0.04 | 2.13 | 4.37 | 3.54 | 0.93 | 2.15 | 1.26 |
| gene-LOC5576966 | trypsin-1 | 1.18 | 0.01 | 6407.02 | 10218.74 | 8631.38 | 2545.47 | 3350.59 | 5014.04 |
| gene-LOC5572968 | 40S ribosomal protein S2 | 1.18 | 0.01 | 1768.09 | 2545.91 | 2271.47 | 715.32 | 810.99 | 1378.02 |
| gene-LOC5565955 | tubulin beta chain | 1.18 | 0.01 | 6.67 | 7.39 | 4.74 | 1.88 | 3.69 | 2.90 |
| gene-LOC5567164 | protein TANC2 | 1.17 | 0.04 | 0.59 | 1.26 | 1.08 | 1.11 | 0.82 | 0.52 |
| gene-LOC5568698 | beta-1,3-glucan-binding protein | 1.17 | 0.01 | 19.57 | 54.83 | 24.67 | 14.59 | 17.92 | 10.59 |
| gene-LOC5578924 | pyrokinin-1 receptor | 1.17 | 0.00 | 47.44 | 49.09 | 72.24 | 16.41 | 33.22 | 14.00 |
| gene-LOC5575863 | cationic amino acid transporter 3 with eGFP tag | 1.17 | 0.00 | 4.00 | 4.76 | 4.58 | 1.76 | 2.22 | 1.88 |
| gene-LOC5576996 | chitinase-3-like protein 1 | 1.17 | 0.00 | 13.07 | 13.95 | 12.47 | 3.61 | 7.59 | 6.13 |
| gene-LOC5570299 | NADPH-dependent 1-acyldihydroxyacetone phosphate reductase | 1.16 | 0.01 | 4.60 | 6.68 | 5.07 | 1.93 | 2.22 | 3.08 |
| gene-LOC5566877 | probable G-protein coupled receptor Mth-like 14 | 1.16 | 0.00 | 2.79 | 4.29 | 2.76 | 1.31 | 1.43 | 1.62 |
| gene-LOC5567014 | lysosomal alpha-mannosidase | 1.16 | 0.03 | 4.03 | 8.39 | 5.42 | 1.23 | 3.26 | 3.45 |
| gene-LOC5573751 | uncharacterized protein LOC5573751 | 1.15 | 0.00 | 68.29 | 98.23 | 66.81 | 30.82 | 39.13 | 33.08 |
| gene-LOC5566777 | dorsal-ventral patterning protein | 1.15 | 0.00 | 1.40 | 2.17 | 1.36 | 0.59 | 0.87 | 0.73 |
| gene-LOC5573386 | neuroendocrine protein 7B2 | 1.15 | 0.00 | 9.36 | 15.03 | 8.51 | 4.52 | 6.71 | 3.36 |
| gene-LOC5565118 | alpha-aminoadipic semialdehyde synthase, mitochondrial | 1.15 | 0.00 | 6.45 | 9.20 | 6.94 | 2.23 | 3.56 | 4.25 |
| gene-LOC5570858 | ATP-binding cassette sub-family G member 4 | 1.15 | 0.02 | 2.76 | 5.11 | 2.43 | 1.37 | 1.79 | 1.42 |
| gene-LOC5569635 | uncharacterized protein LOC5569635 | 1.15 | 0.01 | 37.74 | 71.39 | 44.40 | 31.49 | 23.50 | 12.02 |
| gene-LOC5571319 | protein bowel | 1.15 | 0.03 | 0.83 | 0.71 | 0.68 | 0.32 | 0.39 | 0.28 |
| gene-LOC23687646 | lysosomal alpha-glucosidase | 1.15 | 0.00 | 6.09 | 7.64 | 6.46 | 2.37 | 2.69 | 3.89 |
| gene-LOC5570107 | protein aubergine | 1.14 | 0.00 | 10.78 | 20.15 | 18.39 | 6.57 | 8.43 | 6.98 |
| gene-LOC5575611 | protein hairy | 1.14 | 0.00 | 8.83 | 10.40 | 5.00 | 3.40 | 4.26 | 3.19 |
| gene-LOC5579248 | putative leucine-rich repeat-containing protein DDB_G0290503 | 1.14 | 0.00 | 6.38 | 12.53 | 6.37 | 2.26 | 5.55 | 3.46 |
| gene-LOC5570824 | zinc carboxypeptidase | 1.14 | 0.02 | 4.51 | 9.69 | 5.86 | 2.72 | 3.29 | 2.94 |
| gene-LOC5568027 | chondroitin sulfate synthase 1 | 1.13 | 0.02 | 2.15 | 3.04 | 1.88 | 1.12 | 0.79 | 1.37 |

| | | | | | | | | | |
|---|---|---|---|---|---|---|---|---|---|
| novel5368 | - | 1.13 | 0.02 | 1.64 | 3.08 | 1.77 | 0.90 | 1.30 | 0.72 |
| gene-LOC5575798 | pyruvate dehydrogenase phosphatase regulatory subunit, mitochondrial; AAEL012077-PA, partial | 1.13 | 0.01 | 2.47 | 3.29 | 2.77 | 1.47 | 1.26 | 1.19 |
| gene-LOC5568331 | major egg antigen; heat shock protein beta-1 | 1.13 | 0.00 | 12.92 | 19.73 | 10.42 | 6.10 | 7.97 | 5.32 |
| gene-LOC5576948 | LOW QUALITY PROTEIN: uncharacterized protein LOC5576948 | 1.13 | 0.01 | 1.39 | 1.65 | 1.07 | 0.59 | 0.83 | 0.42 |
| gene-LOC5565474 | probable chitinase 10 | 1.13 | 0.00 | 468.69 | 577.45 | 587.28 | 170.56 | 235.32 | 329.58 |
| gene-LOC5574702 | protocadherin Fat 3 | 1.12 | 0.02 | 3.64 | 3.49 | 2.85 | 1.17 | 2.24 | 1.11 |
| gene-LOC5573853 | serine protease inhibitor 28Dc | 1.12 | 0.01 | 9.44 | 14.17 | 5.67 | 4.98 | 3.67 | 4.95 |
| gene-LOC5567476 | tetraspanin-2A | 1.12 | 0.01 | 10.81 | 14.39 | 10.41 | 3.38 | 5.75 | 7.06 |
| gene-LOC5577025 | methyltransferase-like protein 6 | 1.11 | 0.01 | 14.93 | 23.05 | 13.34 | 4.87 | 8.68 | 9.87 |
| gene-LOC23687865 | protein nubbin; alpha-protein kinase 1; AAEL017445-PA | 1.11 | 0.03 | 0.79 | 0.92 | 0.70 | 0.35 | 0.45 | 0.31 |
| gene-LOC5573117 | probable cytochrome P450 12a4, mitochondrial | 1.11 | 0.04 | 7.20 | 12.78 | 6.62 | 3.23 | 6.26 | 2.84 |
| gene-LOC5575755 | BAI1-associated protein 3 | 1.11 | 0.00 | 1.43 | 1.43 | 0.99 | 0.53 | 0.59 | 0.67 |
| gene-LOC11067929[illegible] | heat shock 70 kDa protein cognate | 1.11 | 0.01 | 5.24 | 11.79 | 6.13 | 2.82 | 4.20 | 3.58 |
| gene-LOC5564720 | protein yellow | 1.11 | 0.01 | 3.48 | 6.42 | 6.63 | 2.05 | 2.58 | 2.93 |
| gene-LOC5575504 | uncharacterized protein LOC5575504 | 1.10 | 0.00 | 13.81 | 17.13 | 10.84 | 4.95 | 6.99 | 6.03 |
| gene-LOC5573200 | uncharacterized protein LOC5573200 | 1.10 | 0.00 | 19.05 | 24.73 | 21.24 | 7.66 | 10.58 | 13.25 |
| gene-LOC5573381 | mitogen-activated protein kinase kinase kinase 4 | 1.10 | 0.01 | 1.27 | 1.78 | 1.49 | 0.81 | 0.66 | 0.63 |
| gene-LOC5568363 | serine protease easter | 1.10 | 0.04 | 5.71 | 7.88 | 3.35 | 1.71 | 3.19 | 2.94 |
| gene-LOC5576274 | titin; smoothelin-like protein 1; AAEL012429-PA, partial | 1.09 | 0.00 | 3.97 | 5.40 | 4.58 | 3.36 | 5.32 | 2.14 |
| gene-LOC5572532 | protein I'm not dead yet | 1.09 | 0.00 | 9.53 | 13.64 | 10.74 | 5.59 | 5.83 | 4.57 |
| gene-LOC11067544[illegible] | biofilm and cell wall regulator 1-like | 1.09 | 0.00 | 189.15 | 339.77 | 271.22 | 107.85 | 120.26 | 128.33 |
| gene-LOC5565450 | maltase A3 | 1.08 | 0.02 | 2.77 | 2.81 | 3.43 | 1.26 | 1.56 | 1.35 |

| | | | | | | | | | |
|---|---|---|---|---|---|---|---|---|---|
| gene-LOC5563777 | vascular endothelial growth factor receptor 1 | 1.08 | 0.00 | 4.42 | 5.66 | 4.23 | 2.25 | 2.31 | 2.50 |
| gene-LOC5575535 | myosin-IB | 1.08 | 0.00 | 22.50 | 23.27 | 19.16 | 7.33 | 13.40 | 10.24 |
| gene-LOC5564097 | RNA polymerase II degradation factor 1 | 1.08 | 0.00 | 32.19 | 40.24 | 23.68 | 14.38 | 20.22 | 10.34 |
| gene-LOC5573038 | dihydropyrimidine dehydrogenase | 1.08 | 0.00 | 4.23 | 5.55 | 5.03 | 2.26 | 2.46 | 2.21 |
| gene-LOC5575831 | aminopeptidase N | 1.07 | 0.00 | 5.46 | 6.46 | 5.07 | 2.06 | 3.57 | 2.34 |
| gene-LOC5566220 | uncharacterized protein LOC5566220 | 1.07 | 0.00 | 76.97 | 98.72 | 70.51 | 26.62 | 37.45 | 50.86 |
| gene-LOC5579075 | ETS-like protein pointed; AAEL003845-PA, partial | 1.07 | 0.03 | 2.18 | 3.37 | 2.44 | 0.79 | 0.68 | 1.49 |
| gene-LOC5568362 | melanization protease 1 | 1.07 | 0.02 | 7.16 | 8.57 | 7.46 | 2.94 | 4.64 | 3.24 |
| gene-LOC11067415 | heat shock protein 70 A1 | 1.07 | 0.03 | 5.18 | 16.74 | 14.67 | 4.64 | 6.67 | 5.98 |
| gene-LOC5579888 | mucin-6 | 1.06 | 0.02 | 1.03 | 1.13 | 0.67 | 0.38 | 0.53 | 0.43 |
| gene-LOC5568577 | putative polypeptide N-acetylgalactosaminyltransferase 9 | 1.06 | 0.02 | 3.33 | 3.56 | 2.81 | 1.83 | 1.12 | 1.61 |
| gene-LOC5573858 | zinc carboxypeptidase A 1 | 1.06 | 0.00 | 37.53 | 67.78 | 48.79 | 14.93 | 24.01 | 33.72 |
| gene-LOC5578590 | uncharacterized protein LOC5578590 | 1.06 | 0.00 | 92.71 | 110.40 | 82.55 | 34.92 | 47.55 | 51.45 |
| gene-LOC11067456 | uncharacterized protein LOC110674569 | 1.06 | 0.00 | 10.45 | 11.85 | 8.54 | 3.49 | 5.30 | 5.76 |
| gene-LOC5576801 | aminopeptidase N | 1.06 | 0.00 | 15.03 | 18.46 | 14.63 | 5.27 | 7.69 | 9.77 |
| gene-LOC5565901 | synaptotagmin 1; hypothetical protein RP20_CCG027412 | 1.06 | 0.00 | 2.70 | 3.73 | 2.91 | 1.45 | 1.69 | 1.37 |
| gene-LOC5564736 | filamin-A; filamin-B | 1.06 | 0.00 | 1.78 | 2.48 | 1.45 | 0.71 | 1.05 | 0.75 |
| novel4956 | glycoprotein | 1.06 | 0.01 | 1.19 | 1.73 | 1.01 | 0.46 | 0.95 | 0.45 |
| gene-LOC5573336 | alkyldihydroxyacetonephosphate synthase | 1.06 | 0.01 | 3.10 | 3.38 | 3.11 | 1.28 | 1.33 | 1.95 |
| gene-LOC5572533 | uncharacterized protein LOC5572533 | 1.06 | 0.00 | 11.56 | 24.60 | 21.80 | 8.45 | 11.18 | 9.37 |
| gene-LOC5580173 | myosin regulatory light chain 2 | 1.05 | 0.00 | 100.55 | 165.61 | 120.77 | 54.80 | 74.43 | 53.61 |
| gene-LOC5565220 | peroxisomal (S)-2-hydroxy-acid oxidase GLO3 | 1.05 | 0.04 | 4.17 | 6.75 | 5.57 | 2.43 | 1.91 | 3.47 |

| | | | | | | | | | |
|---|---|---|---|---|---|---|---|---|---|
| gene-LOC11067650[illegible] | ATP-dependent DNA helicase pfh1-like; uncharacterized protein LOC110676505 | 1.05 | 0.00 | 7.10 | 8.87 | 7.23 | 3.45 | 4.07 | 4.27 |
| gene-LOC5578139 | tissue inhibitor of metalloproteases | 1.05 | 0.03 | 4.86 | 7.78 | 6.55 | 3.44 | 3.16 | 2.10 |
| gene-LOC5564409 | pheromone-processing carboxypeptidase KEX1; uncharacterized protein LOC5564409 isoform X1 | 1.05 | 0.02 | 4.52 | 8.20 | 5.31 | 2.04 | 2.81 | 3.84 |
| novel110 | AAEL007844-PA | 1.05 | 0.04 | 0.46 | 0.57 | 0.46 | 0.20 | 0.26 | 0.25 |
| gene-LOC5564544 | epidermal growth factor receptor | 1.05 | 0.01 | 1.50 | 2.19 | 1.22 | 0.91 | 0.81 | 0.62 |
| gene-LOC11068151[illegible] | diacylglycerol kinase theta | 1.05 | 0.01 | 1.14 | 1.07 | 0.77 | 0.41 | 0.55 | 0.44 |
| gene-LOC5564563 | protein sprouty | 1.05 | 0.02 | 0.73 | 0.95 | 0.59 | 0.51 | 0.32 | 0.31 |
| gene-LOC5569408 | protein toll | 1.05 | 0.04 | 2.61 | 3.48 | 3.21 | 2.37 | 1.24 | 0.82 |
| gene-LOC5571712 | serine protease SP24D | 1.04 | 0.00 | 168.77 | 139.31 | 137.30 | 63.54 | 79.50 | 68.57 |
| gene-LOC5567636 | talin-2 | 1.04 | 0.00 | 3.64 | 3.98 | 3.28 | 1.87 | 2.22 | 1.30 |
| gene-LOC5567811 | division abnormally delayed protein | 1.04 | 0.01 | 2.81 | 2.42 | 2.03 | 1.50 | 0.88 | 0.77 |
| gene-LOC5577765 | uncharacterized protein LOC5577765 | 1.04 | 0.01 | 7.53 | 12.40 | 9.50 | 2.66 | 4.28 | 7.22 |
| gene-LOC5564892 | organic cation transporter protein | 1.04 | 0.03 | 7.18 | 4.52 | 5.79 | 6.29 | 9.10 | 2.83 |
| gene-LOC5564333 | protein amalgam; igLON family member 5 isoform X3 | 1.04 | 0.00 | 9.51 | 10.66 | 5.36 | 3.84 | 4.32 | 4.27 |
| gene-LOC5577230 | Ig-like and fibronectin type-III domain-containing protein 1 | 1.04 | 0.04 | 0.74 | 1.16 | 0.87 | 0.42 | 0.56 | 0.35 |
| gene-LOC5564580 | tetratricopeptide repeat protein 39B | 1.03 | 0.01 | 3.55 | 3.93 | 2.72 | 2.08 | 1.30 | 1.46 |
| gene-LOC5571803 | carbonic anhydrase | 1.03 | 0.00 | 72.09 | 83.76 | 60.93 | 21.53 | 33.50 | 49.22 |
| gene-LOC5566549 | uncharacterized protein LOC5566549 | 1.03 | 0.03 | 1.24 | 1.65 | 1.14 | 0.75 | 0.68 | 0.54 |
| gene-LOC5573481 | uncharacterized protein LOC5573481 | 1.03 | 0.00 | 3.82 | 4.83 | 3.01 | 1.82 | 2.00 | 1.82 |
| gene-LOC5578270 | calsyntenin-1 | 1.03 | 0.01 | 1.13 | 1.63 | 1.18 | 0.67 | 0.72 | 0.52 |
| gene-LOC5566484 | protein CREG1 | 1.03 | 0.00 | 93.19 | 133.23 | 101.98 | 35.66 | 56.76 | 66.32 |
| gene-LOC5576917 | uncharacterized protein LOC5576917 | 1.03 | 0.00 | 15.08 | 24.87 | 21.66 | 8.80 | 9.80 | 10.90 |
| gene-LOC5579338 | integrin alpha-PS1 | 1.02 | 0.01 | 4.01 | 3.95 | 2.55 | 1.66 | 2.25 | 1.10 |
| gene-LOC5571582 | protein CREBRF homolog | 1.02 | 0.00 | 3.30 | 4.35 | 3.03 | 1.65 | 2.14 | 1.38 |

| | | | | | | | | | |
|---|---|---|---|---|---|---|---|---|---|
| gene-LOC5577377 | tryptophan 2,3-dioxygenase | 1.02 | 0.00 | 10.84 | 20.91 | 15.21 | 6.24 | 8.73 | 7.80 |
| gene-LOC5580182 | prion-like-(Q/N-rich) domain-bearing protein 25 | 1.02 | 0.01 | 2.72 | 3.34 | 3.61 | 1.90 | 1.46 | 1.37 |
| gene-LOC5577252 | excitatory amino acid transporter 1 | 1.02 | 0.02 | 4.34 | 6.17 | 3.77 | 2.14 | 2.19 | 2.66 |
| gene-LOC5571275 | neurogenic locus notch homolog protein 1 | 1.01 | 0.00 | 26.97 | 27.36 | 29.09 | 12.37 | 11.80 | 18.45 |
| gene-LOC5568588 | phosphoserine phosphatase | 1.01 | 0.05 | 8.69 | 11.23 | 11.51 | 2.84 | 4.66 | 8.04 |
| gene-LOC5578307 | trypsin alpha-3 | 1.01 | 0.00 | 128.08 | 208.53 | 133.06 | 52.78 | 81.20 | 94.36 |
| gene-LOC5571093 | uncharacterized protein LOC5571093 | 1.01 | 0.00 | 23.70 | 27.02 | 24.19 | 10.66 | 11.68 | 14.13 |
| gene-LOC23687722 | farnesol dehydrogenase | 1.01 | 0.00 | 23.90 | 38.99 | 23.66 | 13.72 | 13.83 | 14.59 |
| gene-LOC5566251 | venom dipeptidyl peptidase 4 isoform X1 | 1.00 | 0.00 | 11.73 | 16.03 | 13.75 | 6.43 | 7.21 | 7.05 |
| gene-LOC5564732 | venom allergen 5 | 1.00 | 0.03 | 4.06 | 8.22 | 4.74 | 2.12 | 2.90 | 2.10 |
| gene-LOC5569925 | short-chain dehydrogenase/reductase family 16C member 6; AAEL008016-PA | 1.00 | 0.00 | 25.87 | 33.49 | 23.07 | 9.42 | 15.92 | 15.39 |
| gene-LOC5577749 | EKC/KEOPS complex subunit | -10.85 | 0.04 | 0.00 | 0.00 | 0.00 | 47.08 | 26.33 | 0.00 |
| gene-LOC11068033[illegible] | uncharacterized protein LOC110680336 | -7.43 | 0.00 | 0.00 | 0.00 | 0.00 | 3.72 | 2.74 | 0.34 |
| gene-LOC11067978[illegible] | histone H2B | -6.94 | 0.02 | 0.35 | 0.31 | 3.66 | 267.61 | 236.42 | 0.00 |
| novel7731 | - | -6.45 | 0.02 | 0.00 | 0.00 | 0.00 | 2.60 | 3.28 | 0.00 |
| gene-LOC11068088[illegible] | aminopeptidase N | -4.96 | 0.00 | 0.00 | 0.00 | 0.17 | 0.89 | 2.12 | 2.23 |
| gene-LOC11068130[illegible] | tubby-related protein 4 | -4.82 | 0.00 | 0.00 | 0.04 | 0.00 | 0.67 | 0.19 | 0.34 |
| gene-LOC5576382 | sugar transporter ERD6-like 6 | -3.87 | 0.02 | 0.00 | 0.00 | 0.36 | 2.50 | 1.61 | 0.54 |
| novel5094 | uncharacterized protein LOC5572259 | -3.33 | 0.03 | 0.00 | 1.47 | 0.00 | 4.16 | 6.50 | 4.38 |
| gene-LOC5568895 | fatty acyl-CoA reductase wat | -3.00 | 0.02 | 0.09 | 0.00 | 0.38 | 1.50 | 1.65 | 0.53 |
| gene-LOC11067604[illegible] | uncharacterized protein LOC110676044 | -2.86 | 0.05 | 0.37 | 0.06 | 1.71 | 0.28 | 8.78 | 5.91 |
| gene-LOC5571499 | uncharacterized protein LOC5571499 | -2.65 | 0.00 | 0.78 | 1.31 | 0.71 | 1.03 | 6.81 | 9.64 |
| gene-LOC5565217 | protein Fe65 homolog | -2.17 | 0.01 | 2.76 | 0.85 | 1.61 | 7.31 | 5.12 | 25.05 |
| novel2658 | - | -1.98 | 0.04 | 10.41 | 21.52 | 58.60 | 96.47 | 103.05 | 141.60 |

| | | | | | | | | | |
|---|---|---|---|---|---|---|---|---|---|
| gene-LOC5576721 | COX assembly mitochondrial protein 2 homolog | -1.76 | 0.01 | 5.66 | 7.85 | 10.04 | 41.35 | 38.56 | 5.35 |
| novel592 | conserved hypothetical protein | -1.75 | 0.02 | 1.76 | 1.17 | 0.93 | 3.45 | 6.56 | 2.23 |
| novel2422 | uncharacterized protein LOC109412483 isoform X1 | -1.65 | 0.05 | 1.32 | 1.87 | 0.17 | 3.33 | 3.83 | 3.20 |
| novel2345 | - | -1.65 | 0.00 | 1.35 | 2.25 | 1.58 | 3.33 | 3.89 | 8.83 |
| novel5425 | - | -1.63 | 0.01 | 10.24 | 17.46 | 20.29 | 64.76 | 54.28 | 20.53 |
| gene-LOC11068095( | nose resistant to fluoxetine protein | -1.53 | 0.04 | 1.44 | 2.74 | 1.79 | 5.57 | 9.24 | 1.91 |
| novel5537 | - | -1.46 | 0.02 | 4.94 | 6.11 | 8.86 | 16.10 | 19.46 | 15.50 |
| novel145 | - | -1.45 | 0.00 | 278.45 | 461.84 | 367.96 | 1244.37 | 706.32 | 790.72 |
| gene-LOC5571232 | uncharacterized protein LOC5571232; AAEL008922-PB | -1.43 | 0.00 | 6.71 | 7.87 | 11.54 | 14.97 | 29.54 | 32.60 |
| gene-LOC11067918: | uncharacterized protein LOC110679183 | -1.42 | 0.05 | 1.19 | 1.70 | 0.76 | 2.44 | 5.71 | 1.60 |
| gene-LOC5579971 | protein AF-9 | -1.42 | 0.00 | 6.10 | 8.63 | 10.41 | 20.34 | 20.96 | 24.40 |
| novel5307 | - | -1.37 | 0.00 | 3.69 | 4.99 | 4.96 | 9.66 | 12.92 | 11.89 |
| novel416 | AAEL001348-PA | -1.35 | 0.01 | 3.86 | 2.71 | 3.43 | 13.35 | 6.10 | 5.38 |
| gene-LOC11068092 | pikachurin; AAEL013602-PA, | -1.32 | 0.02 | 3.23 | 2.45 | 2.31 | 7.77 | 7.91 | 3.77 |
| gene-LOC5573276 | uncharacterized protein LOC5573276 | -1.31 | 0.05 | 3.08 | 6.61 | 2.87 | 11.39 | 15.49 | 3.63 |
| gene-LOC5572901 | protein cornichon homolog 4 | -1.27 | 0.00 | 6.18 | 4.86 | 8.12 | 16.84 | 12.13 | 13.82 |
| novel8110 | TBC1 domain family member 13 isoform X2 | -1.27 | 0.04 | 4.60 | 6.90 | 9.41 | 21.42 | 15.27 | 11.53 |
| novel5457 | - | -1.24 | 0.02 | 11.03 | 3.12 | 7.91 | 21.23 | 16.68 | 14.94 |
| novel8189 | - | -1.23 | 0.01 | 10.10 | 8.78 | 10.43 | 27.32 | 18.92 | 12.14 |
| gene-LOC11067865 | teneurin-a | -1.20 | 0.00 | 2.08 | 3.46 | 2.81 | 11.94 | 7.29 | 4.93 |
| gene-LOC5579522 | protein Wnt-5 | -1.20 | 0.00 | 2.26 | 1.22 | 2.15 | 5.44 | 4.05 | 2.86 |
| novel5083 | - | -1.16 | 0.00 | 24.82 | 27.55 | 27.46 | 67.93 | 42.17 | 37.50 |
| gene-LOC5564154 | uncharacterized protein LOC5564154 | -1.14 | 0.00 | 2.12 | 2.80 | 2.26 | 4.46 | 5.19 | 6.04 |
| gene-LOC5569370 | ras-GEF domain-containing family member 1B; uncharacterized protein LOC115267902 | -1.13 | 0.02 | 1.75 | 2.29 | 1.13 | 4.32 | 4.40 | 2.26 |
| gene-LOC11067629 | nucleoside diphosphate kinase-like | -1.12 | 0.03 | 30.17 | 13.23 | 10.98 | 36.93 | 29.19 | 48.44 |

| | | | | | | | | | |
|---|---|---|---|---|---|---|---|---|---|
| gene-LOC11067600 | uncharacterized protein LOC110676008 | -1.07 | 0.01 | 4.18 | 4.20 | 5.26 | 5.56 | 11.80 | 10.84 |
| gene-LOC5572866 | COMM domain-containing protein 3 | -1.06 | 0.03 | 4.61 | 5.63 | 6.21 | 9.87 | 14.26 | 11.47 |
| gene-LOC11067818 | uncharacterized protein K02A2.6-like; uncharacterized protein LOC110678183 | -1.05 | 0.01 | 13.42 | 8.03 | 9.11 | 15.90 | 22.19 | 31.58 |
| gene-LOC5568988 | 2-oxoisovalerate dehydrogenase subunit beta, mitochondrial-like; hypothetical protein RP20_CCG014130; AAEL007302-PA; O-acyltransferase like protein isoform X1 | -1.04 | 0.01 | 5.05 | 3.49 | 4.42 | 8.33 | 10.62 | 4.85 |
| gene-LOC11067936 | uncharacterized protein LOC110679361 | -1.04 | 0.00 | 7.23 | 4.07 | 6.61 | 13.13 | 14.05 | 8.93 |
| gene-LOC5566889 | protein-cysteine N-palmitoyltransferase Rasp | -1.02 | 0.01 | 5.73 | 4.15 | 5.39 | 12.79 | 11.12 | 6.30 |
| gene-LOC5574086 | collagen alpha-1(XV) chain; collagen alpha-1(XVIII) chain | -1.01 | 0.01 | 4.24 | 2.22 | 4.55 | 10.97 | 7.99 | 6.76 |

**Table S3. Differentially expressed genes that consistently had differential expression in TIBOV-infected mosquitoes at 2 and 7 dpi.**

| Gene_name | NR annotation | TIBOV-2d/MOCK-2d Log2(FoldChange) | TIBOV-7d/MOCK-7d Log2(FoldChange) |
|---|---|---|---|
| gene-LOC5570856 | stress-activated protein kinase JNK | -2.87 | 6.10 |
| gene-LOC5576909 | uncharacterized protein LOC5576909 | -2.87 | 5.67 |
| gene-LOC5571242 | hornerin | -2.47 | 5.49 |
| gene-LOC5573896 | uricase | -3.25 | 5.24 |
| gene-LOC5576981 | venom allergen 5; AAEL003053-PF | -1.03 | 5.00 |
| gene-LOC5575351 | tryptase | -2.13 | 4.42 |
| gene-LOC23687564 | glycine-rich protein 5 | -2.59 | 4.39 |
| gene-LOC5570845 | kallikrein 1-related peptidase b3 | -1.01 | 4.19 |
| gene-LOC5575349 | tryptase | -1.66 | 3.86 |
| gene-LOC5578510 | trypsin 5G1 | -2.25 | 3.84 |
| gene-LOC5570039 | uncharacterized protein LOC109400458 isoform X1/X2 | -1.28 | 3.81 |
| gene-LOC5578210 | uncharacterized protein LOC5578210 | -1.51 | 3.80 |
| gene-LOC5566826 | adenosine deaminase 2 | -1.09 | 3.78 |
| gene-LOC5570482 | leucine-rich repeat transmembrane neuronal protein 3 | -1.40 | 3.77 |
| gene-LOC5570483 | acidic leucine-rich nuclear phosphoprotein 32 family member A | -1.24 | 3.74 |
| gene-LOC5569107 | uncharacterized protein LOC5569107 | -2.63 | 3.73 |
| gene-LOC110674832 | uncharacterized protein LOC110674832 | -1.21 | 3.66 |
| gene-LOC5565434 | leucine-rich repeat-containing protein 15 | -1.75 | 3.61 |
| gene-LOC5575814 | uncharacterized protein LOC5575814 | -2.33 | 3.60 |
| gene-LOC110674731 | serine protease snake-like isoform | -1.56 | 3.50 |
| gene-LOC110677017 | uncharacterized protein LOC110677017 | -1.02 | 3.50 |
| gene-LOC5575552 | arginase, hepatic | -1.57 | 3.50 |
| gene-LOC5563550 | serine protease easter | -1.54 | 3.39 |
| gene-LOC5579410 | serine protease easter | -2.69 | 3.34 |
| gene-LOC5565301 | protein BTG1 | -1.50 | 2.99 |
| gene-LOC5571025 | dynein assembly factor 1, axonemal homolog | -1.20 | 2.92 |
| gene-LOC5579042 | uncharacterized protein LOC5579042 | -1.35 | 2.91 |
| gene-LOC5564510 | cytochrome b5-related protein | -1.20 | 2.87 |
| gene-LOC5578506 | trypsin 5G1-like | -1.52 | 2.83 |
| gene-LOC5563617 | serine protease easter | -2.25 | 2.83 |
| gene-LOC5574523 | cholesterol 7-desaturase | -1.15 | 2.81 |
| gene-LOC5570479 | ıbrane protein FLRT3 isoform X2; leucine-rich repeat transmembrane r | -1.63 | 2.79 |
| gene-LOC5578266 | transcription factor AP-1 isoform X1/X2 | -1.15 | 2.73 |
| gene-LOC5574170 | tryptase isoform X1/X2 | -1.69 | 2.71 |

| | | | |
|---|---|---|---|
| gene-LOC5569606 | uncharacterized protein LOC5569606 | -1.83 | 2.71 |
| gene-LOC5573649 | uncharacterized protein LOC5573649 | -3.48 | 2.67 |
| gene-LOC5564764 | cytochrome P450 9e2 | -1.60 | 2.61 |
| gene-LOC5570847 | 37 kDa salivary gland allergen Aed a 2-like | -1.38 | 2.58 |
| gene-LOC5570229 | fatty acid synthase | -1.30 | 2.57 |
| gene-LOC110679583 | myb-like protein V | -1.35 | 2.56 |
| gene-LOC5567194 | surface antigen CRP170 | -1.61 | 2.52 |
| gene-LOC5571641 | laccase-2 | -1.70 | 2.48 |
| gene-LOC5565763 | uncharacterized protein LOC5565763 | -1.41 | 2.42 |
| gene-LOC5577716 | protein lethal(2)essential for life | -1.03 | 2.39 |
| gene-LOC110675609 | uncharacterized protein LOC110675609 | -1.54 | 2.35 |
| gene-LOC5571297 | 2-hydroxyacylsphingosine 1-beta-galactosyltransferase | -1.52 | 2.29 |
| gene-LOC5575325 | transmembrane protease serine 2 | -2.12 | 2.29 |
| gene-LOC5573927 | fatty acid synthase | -1.06 | 2.28 |
| gene-LOC5567003 | serine protease snake isoform X1/X2 | -1.19 | 2.27 |
| gene-LOC5572848 | putative uncharacterized protein DDB_G0277255 | -1.45 | 2.26 |
| gene-LOC5569163 | cysteine dioxygenase type 1 | -1.58 | 2.21 |
| gene-LOC5571325 | tyrosine aminotransferase | -1.86 | 2.19 |
| gene-LOC5571667 | uncharacterized protein LOC5571667 isoform X1/X2 | -1.29 | 2.17 |
| gene-LOC5567327 | disintegrin and metalloproteinase domain-containing protein 12 | -1.06 | 2.17 |
| gene-LOC5576459 | uncharacterized protein LOC5576459 | -2.06 | 2.15 |
| gene-LOC5574469 | esterase B1 | -1.42 | 2.10 |
| gene-LOC5578672 | sodium-dependent nutrient amino acid transporter 1 isoform X1/X2 | -2.05 | 2.09 |
| gene-LOC5575393 | venom allergen 5 | -1.44 | 2.06 |
| gene-LOC5567584 | general odorant-binding protein 83a | -1.16 | 2.06 |
| gene-LOC5564054 | UNC93-like protein | -1.55 | 2.06 |
| gene-LOC5563663 | serine protease easter | -1.86 | 2.02 |
| gene-LOC5568623 | uncharacterized protein LOC5568623 | -3.76 | 2.00 |
| gene-LOC110679707 | serine protease 7-like isoform X1/X2 | -1.23 | 2.00 |
| gene-LOC23687754 | flocculation protein FLO11 | -1.62 | 1.99 |
| gene-LOC5576245 | tubulin beta-3 chain | -1.15 | 1.98 |
| gene-LOC5576475 | uncharacterized protein LOC5576475 | -1.03 | 1.98 |
| gene-LOC110676173 | uncharacterized protein LOC110676173 | -1.80 | 1.97 |
| gene-LOC5572116 | Niemann-Pick type protein homolog 1B | -1.64 | 1.97 |
| gene-LOC5567033 | glucose dehydrogenase | -1.50 | 1.97 |
| gene-LOC5576417 | uncharacterized protein LOC5576417 | -1.34 | 1.96 |
| novel2139 | - | -1.05 | 1.93 |
| gene-LOC5571279 | peptidyl-alpha-hydroxyglycine alpha-amidating lyase 1 | -1.19 | 1.89 |

| | | | |
|---|---|---|---|
| gene-LOC5575341 | general odorant-binding protein 56d | -1.08 | 1.87 |
| gene-LOC5575399 | venom allergen 5 | -1.53 | 1.87 |
| gene-LOC5575338 | leucine-rich repeat neuronal protein 1 | -1.22 | 1.87 |
| gene-LOC5571916 | putative helicase MOV-10 | -1.40 | 1.86 |
| gene-LOC5571053 | serine protease SP24D | -1.82 | 1.85 |
| gene-LOC5572428 | AAEL001794-PA/PB, partial; CD109 antigen | -3.64 | 1.85 |
| gene-LOC5578871 | uncharacterized protein LOC5578871 | -2.10 | 1.84 |
| gene-LOC5569637 | leucine-rich repeat-containing G-protein coupled receptor 4 | -2.16 | 1.83 |
| gene-LOC5564497 | uncharacterized protein LOC5564497 | -1.38 | 1.82 |
| gene-LOC5568783 | facilitated trehalose transporter Tret1 | -1.21 | 1.81 |
| gene-LOC5576084 | 2-amino-3-ketobutyrate coenzyme A ligase, mitochondrial | -1.18 | 1.77 |
| gene-LOC5578664 | AAEL003626-PA; sodium-dependent nutrient amino acid transporter 1 | -1.62 | 1.76 |
| gene-LOC5568175 | uncharacterized protein LOC5568175 | -1.49 | 1.76 |
| gene-LOC23687443 | AAEL017023-PA, partial; CD109 antigen | -3.04 | 1.75 |
| novel2571 | AAEL003593-PA | -1.66 | 1.73 |
| gene-LOC5573843 | uncharacterized protein LOC5573843 | -1.37 | 1.67 |
| gene-LOC5568744 | pyridoxal phosphate phosphatase isoform X1/X2 | -1.12 | 1.67 |
| novel5021 | putative salivary secreted peptide | -1.08 | 1.66 |
| gene-LOC5578728 | uncharacterized protein LOC5578728 | -1.13 | 1.66 |
| gene-LOC5570040 | Vago protein; uncharacterized protein LOC5570040 | -1.17 | 1.66 |
| gene-LOC5570911 | acylphosphatase-2 | -1.45 | 1.64 |
| gene-LOC5569608 | uncharacterized protein LOC5569608 isoform X1/X2 | -1.39 | 1.55 |
| gene-LOC5574136 | alpha-tocopherol transfer protein-like | -1.12 | 1.55 |
| gene-LOC110675610 | uncharacterized protein LOC110675610 | -1.31 | 1.54 |
| gene-LOC5575342 | general odorant-binding protein 56d | -1.39 | 1.52 |
| gene-LOC5563689 | pacifastin-like protease inhibitor cvp4 | -1.32 | 1.51 |
| gene-LOC5571127 | uncharacterized protein LOC5571127 isoform X1-X3 | -1.43 | 1.51 |
| gene-LOC5577270 | guanine nucleotide-binding protein G(f) subunit alpha | -1.49 | 1.50 |
| gene-LOC5573776 | gamma-glutamyl hydrolase isoform X1-X3 | -1.29 | 1.49 |
| gene-LOC5576180 | general odorant-binding protein 72 | -1.25 | 1.49 |
| gene-LOC5578692 | serine protease easter | -1.12 | 1.49 |
| gene-LOC5571433 | LOW QUALITY PROTEIN: cholinesterase | -1.06 | 1.48 |
| gene-LOC5572129 | transient receptor potential cation channel protein painless isoform X1 | -2.01 | 1.48 |
| gene-LOC5572108 | uncharacterized protein LOC5572108 isoform X1-X4 | -2.40 | 1.45 |
| gene-LOC5571779 | lachesin | -1.03 | 1.43 |
| gene-LOC5567410 | shootin-1 | -1.26 | 1.39 |
| gene-LOC5573074 | venom allergen 5 isoform X1/X2 | -1.32 | 1.38 |
| novel2418 | - | -1.19 | 1.36 |

| | | | |
|---|---|---|---|
| gene-LOC5578738 | glia-derived nexin | -1.43 | 1.34 |
| gene-LOC5573855 | zinc carboxypeptidase A 1 | -1.52 | 1.33 |
| gene-LOC5570819 | CD109 antigen isoform X1-X12 | -1.51 | 1.32 |
| gene-LOC5567097 | uridine phosphorylase 1 isoform X1/X2 | -1.04 | 1.30 |
| gene-LOC5568779 | facilitated trehalose transporter Tret1-2 homolog isoform X1/X2 | -1.33 | 1.29 |
| gene-LOC5573760 | mini-chromosome maintenance complex-binding protein; lysozyme | -1.15 | 1.27 |
| gene-LOC110674150 | heat shock protein 70 A1 | -1.73 | 1.26 |
| gene-LOC5577601 | larval cuticle protein LCP-30 | -1.62 | 1.25 |
| novel2199 | AAEL005098-PA | -1.42 | 1.25 |
| gene-LOC5578455 | uncharacterized protein LOC5578455 | -1.78 | 1.25 |
| gene-LOC5565699 | beta-hexosaminidase subunit beta isoform X1/X2 | -1.54 | 1.19 |
| gene-LOC5578105 | regulator of microtubule dynamics protein 1 | -1.37 | 1.19 |
| gene-LOC5566877 | probable G-protein coupled receptor Mth-like 14 | -1.04 | 1.16 |
| gene-LOC5573853 | serine protease inhibitor 28Dc | -1.19 | 1.12 |
| gene-LOC5568362 | melanization protease 1 | -1.12 | 1.07 |
| gene-LOC110674151 | heat shock protein 70 A1-like | -1.69 | 1.07 |
| gene-LOC5579888 | mucin-6 isoform X1/X2 | -1.20 | 1.06 |
| novel4956 | glycoprotein | -1.01 | 1.06 |
| gene-LOC5564892 | organic cation transporter protein isoform X1/X2 | -1.27 | 1.04 |
| novel5083 | - | 1.40 | -1.16 |
| gene-LOC5576721 | COX assembly mitochondrial protein 2 homolog | -1.25 | -1.76 |

**Table S4. Enriched GO categories linked to DEGs of TIBOV-infected mosquitoes at 2 dpi were listed when Qvalue ≤ 0.0001.**

| ID | Descrption | Classification | Qvalue | Total number of genes | Number of upregulated genes | Number of downregulated genes |
|---|---|---|---|---|---|---|
| GO:0006952 | defense response | Biological Process | 0.00 | 37 | 0 | 8 |
| GO:0045087 | innate immune response | Biological Process | 0.00 | 28 | 0 | 6 |
| GO:0006955 | immune response | Biological Process | 0.00 | 31 | 0 | 6 |
| GO:0002376 | immune system process | Biological Process | 0.00 | 34 | 0 | 6 |
| GO:0009607 | response to biotic stimulus | Biological Process | 0.00 | 14 | 0 | 4 |
| GO:0009617 | response to bacterium | Biological Process | 0.00 | 14 | 0 | 4 |
| GO:0042742 | defense response to bacterium | Biological Process | 0.00 | 14 | 0 | 4 |
| GO:0043207 | response to external biotic stimulus | Biological Process | 0.00 | 14 | 0 | 4 |
| GO:0051707 | response to other organism | Biological Process | 0.00 | 14 | 0 | 4 |
| GO:0098542 | defense response to other organism | Biological Process | 0.00 | 14 | 0 | 4 |
| GO:0005576 | extracellular region | Cellular Component | 0.00 | 409 | 1 | 43 |
| GO:0005615 | extracellular space | Cellular Component | 0.00 | 53 | 0 | 8 |
| GO:0044421 | extracellular region part | Cellular Component | 0.00 | 57 | 0 | 8 |
| GO:0004252 | serine-type endopeptidase activity | Molecular Function | 0.00 | 349 | 0 | 40 |
| GO:0008236 | serine-type peptidase activity | Molecular Function | 0.00 | 373 | 0 | 41 |
| GO:0017171 | serine hydrolase activity | Molecular Function | 0.00 | 373 | 0 | 41 |
| GO:0004175 | endopeptidase activity | Molecular Function | 0.00 | 474 | 0 | 43 |
| GO:0008233 | peptidase activity | Molecular Function | 0.00 | 687 | 0 | 48 |
| GO:0070011 | peptidase activity, acting on L-amino acid peptides | Molecular Function | 0.00 | 678 | 0 | 47 |
| GO:0016787 | hydrolase activity | Molecular Function | 0.00 | 1811 | 0 | 79 |
| GO:0003824 | catalytic activity | Molecular Function | 0.00 | 3828 | 0 | 110 |
| GO:0030414 | peptidase inhibitor activity | Molecular Function | 0.00 | 59 | 0 | 8 |
| GO:0061134 | peptidase regulator activity | Molecular Function | 0.00 | 61 | 0 | 8 |

**Table S5. Enriched GO categories linked to DEGs of TIBOV-infected mosquitoes at 7 dpi were listed when Qvalue ≤ 0.0001.**

| ID | Descrption | Classification | Qvalue | Total number of genes | Number of upregulated genes | Number of downregulated genes |
|---|---|---|---|---|---|---|
| GO:0009314 | response to radiation | Biological Process | 0.00 | 13 | 6 | 0 |
| GO:0009416 | response to light stimulus | Biological Process | 0.00 | 13 | 6 | 0 |
| GO:1901565 | organonitrogen compound catabolic process | Biological Process | 0.00 | 69 | 12 | 0 |
| GO:0044712 | single-organism catabolic process | Biological Process | 0.00 | 151 | 17 | 1 |
| GO:1901605 | alpha-amino acid metabolic process | Biological Process | 0.00 | 72 | 12 | 0 |
| GO:0005576 | extracellular region | Cellular Component | 0.00 | 409 | 55 | 1 |
| GO:0031224 | intrinsic component of membrane | Cellular Component | 0.00 | 3053 | 169 | 9 |
| GO:0016021 | integral component of membrane | Cellular Component | 0.00 | 3036 | 168 | 9 |
| GO:0044421 | extracellular region part | Cellular Component | 0.00 | 57 | 16 | 0 |
| GO:0016020 | membrane | Cellular Component | 0.00 | 3411 | 182 | 9 |
| GO:0044425 | membrane part | Cellular Component | 0.00 | 3175 | 171 | 9 |
| GO:0005615 | extracellular space | Cellular Component | 0.00 | 53 | 14 | 0 |
| GO:0016787 | hydrolase activity | Molecular Function | 0.00 | 1811 | 147 | 2 |
| GO:0070011 | peptidase activity, acting on L-amino acid peptides | Molecular Function | 0.00 | 678 | 74 | 2 |
| GO:0008233 | peptidase activity | Molecular Function | 0.00 | 687 | 74 | 2 |
| GO:0003824 | catalytic activity | Molecular Function | 0.00 | 3828 | 237 | 7 |
| GO:0008236 | serine-type peptidase activity | Molecular Function | 0.00 | 373 | 46 | 1 |
| GO:0017171 | serine hydrolase activity | Molecular Function | 0.00 | 373 | 46 | 1 |
| GO:0004252 | serine-type endopeptidase activity | Molecular Function | 0.00 | 349 | 43 | 1 |
| GO:0004175 | endopeptidase activity | Molecular Function | 0.00 | 474 | 49 | 1 |
| GO:0008238 | exopeptidase activity | Molecular Function | 0.00 | 108 | 18 | 1 |
| GO:0004866 | endopeptidase inhibitor activity | Molecular Function | 0.00 | 55 | 12 | 0 |
| GO:0061135 | endopeptidase regulator activity | Molecular Function | 0.00 | 55 | 12 | 0 |
| GO:0008237 | metallopeptidase activity | Molecular Function | 0.00 | 181 | 22 | 1 |
| GO:0030414 | peptidase inhibitor activity | Molecular Function | 0.00 | 59 | 12 | 0 |
| GO:0061134 | peptidase regulator activity | Molecular Function | 0.00 | 61 | 12 | 0 |
| GO:0016798 | hydrolase activity, acting on glycosyl bonds | Molecular Function | 0.00 | 105 | 16 | 0 |

**Table S6 The correlation between TIBOV-induced cytopathic effect in BHK-21 cells and CT values of virus RNA by qRT-PCR.**

| TIBOV titer (PFU/mL) | Virus dose used to inoculate plate (PFU) | CPE | Days post infection that CPE appearance | Ct values |
|---|---|---|---|---|
| $3.7\times10^{6}$ | $3.7\times10^{5}$ | Yes | 2 | 13.71 |
| $3.7\times10^{5}$ | $3.7\times10^{4}$ | Yes | 3 | 17.04 |
| $3.7\times10^{4}$ | $3.7\times10^{3}$ | Yes | 4 | 20.4 |
| $3.7\times10^{3}$ | $3.7\times10^{2}$ | Yes | 4 | 23.86 |
| $3.7\times10^{2}$ | $3.7\times10^{1}$ | Yes | 4 | 27.23 |
| $3.7\times10^{1}$ | $3.7\times10^{0.5}$ | Yes | 5 | 30.62 |
| $3.7\times10^{0.5}$ | $3.7\times10^{0}$ | Yes | 5 | 31.29 |
| $3.7\times10^{0}$ | $3.7\times10^{-0.5}$ | Yes | 6 | 34.16 |
| $3.7\times10^{-0.5}$ | $3.7\times10^{-1}$ | Yes | 7 | 35.1 |
| $3.7\times10^{-1}$ | $3.7\times10^{-2}$ | No | - | 37.13 |
